# Supplementary material for: Effects of interorganisational information technology networks on patient safety: a realist synthesis
Source: BMJ Open. 2020 Oct 10;10(10):e036608. doi: 10.1136/bmjopen-2019-036608 (PMC7552839; doi:10.1136/bmjopen-2019-036608)
Supplement: Author's manuscript [file bmjopen-2019-036608.draft_revisions.pdf]

BMJ Open is committed to open peer review. As part of this commitment we make the peer review history of every article we publish publicly available.

When an article is published we post the peer reviewers' comments and the authors' responses online. We also post the versions of the paper that were used during peer review. These are the versions that the peer review comments apply to.

The versions of the paper that follow are the versions that were submitted during the peer review process. They are not the versions of record or the final published versions. They should not be cited or distributed as the published version of this manuscript.

BMJ Open is an open access journal and the full, final, typeset and author-corrected version of record of the manuscript is available on our site with no access controls, subscription charges or pay-per-view fees (http://bmjopen.bmj.com).

If you have any questions on BMJ Open's open peer review process please email info.bmjopen@bmj.com

BMJ Open

The effects of inter-organisational information technology networks on patient safety: a realist synthesis

Journal:	*BMJ Open*
Manuscript ID	bmjopen-2019-036608
Article Type:	Original research
Date Submitted by the Author:	07-Jan-2020
Complete List of Authors:	Keen, Justin; University of Leeds, Leeds Institute of Health Sciences<br>Abdulwahid, Maysam; University of Leeds<br>King, Natalie; University of Leeds, Leeds Institute of Health Sciences<br>Wright, Judy; University of Leeds, Leeds Institute of Health Sciences<br>Randell, Rebecca; University of Bradford, Faculty of Health Studies<br>Gardner, Peter; University of Leeds Institute of Psychological Sciences<br>Waring, Justin ; University of Birmingham<br>Longo, Roberta; University of Leeds, Leeds Institute of Health Sciences<br>Nikolova, Silviya; Leeds Institute of Health Sciences<br>Sloan, Claire; University of Leeds, Leeds Institute of Health Sciences<br>Greenhalgh, Joanne; University of Leeds, Sociology and Social Policy
Keywords:	Information technology < BIOTECHNOLOGY & BIOINFORMATICS, Health policy < HEALTH SERVICES ADMINISTRATION & MANAGEMENT, Quality in health care < HEALTH SERVICES ADMINISTRATION & MANAGEMENT, Risk management < HEALTH SERVICES ADMINISTRATION & MANAGEMENT

SCHOLARONE™
Manuscripts

I, the Submitting Author has the right to grant and does grant on behalf of all authors of the Work (as defined in the below author licence), an exclusive licence and/or a non-exclusive licence for contributions from authors who are: i) UK Crown employees; ii) where BMJ has agreed a CC-BY licence shall apply, and/or iii) in accordance with the terms applicable for US Federal Government officers or employees acting as part of their official duties; on a worldwide, perpetual, irrevocable, royalty-free basis to BMJ Publishing Group Ltd ("BMJ") its licensees and where the relevant Journal is co-owned by BMJ to the co-owners of the Journal, to publish the Work in this journal and any other BMJ products and to exploit all rights, as set out in our licence.

The Submitting Author accepts and understands that any supply made under these terms is made by BMJ to the Submitting Author unless you are acting as an employee on behalf of your employer or a postgraduate student of an affiliated institution which is paying any applicable article publishing charge ("APC") for Open Access articles. Where the Submitting Author wishes to make the Work available on an Open Access basis (and intends to pay the relevant APC), the terms of reuse of such Open Access shall be governed by a Creative Commons licence – details of these licences and which Creative Commons licence will apply to this Work are set out in our licence referred to above.

Other than as permitted in any relevant BMJ Author's Self Archiving Policies, I confirm this Work has not been accepted for publication elsewhere, is not being considered for publication elsewhere and does not duplicate material already published. I confirm all authors consent to publication of this Work and authorise the granting of this licence.

For peer review only

The effects of inter-organisational information technology networks on patient safety: a realist synthesis

Justin Keen
Leeds Institute of Health Sciences
University of Leeds
Worsley Building
Clarendon Way
Leeds LS2 9NL
England

j.keen@leeds.ac.uk
0113 343 0939
(No fax number available)

Maysam Abdulwahid
Leeds Institute of Health Sciences
University of Leeds
Leeds
England

Natalie King
Leeds Institute of Health Sciences
University of Leeds
Leeds
England

Judy Wright
Leeds Institute of Health Sciences
University of Leeds

For peer review only

Leeds
England

Rebecca Randell
School of Healthcare
University of Leeds
Leeds
England

Peter Gardner
School of Psychology
University of Leeds
Leeds
England

Justin Waring
Health Services Management Centre
University of Birmingham
Birmingham
England

Roberta Longo
Leeds Institute of Health Sciences
University of Leeds
Leeds
England

Silviya Nikolova
Leeds Institute of Health Sciences
University of Leeds

Claire Sloan
Leeds Institute of Health Sciences
University of Leeds

For peer review only

For peer review only - http://bmjopen.bmj.com/site/about/guidelines.xhtml

Leeds
England

Joanne Greenhalgh
School of Sociology and Social Policy
University of Leeds
Leeds
England

For peer review only

Key words

information technology, health information exchange, patient safety, systematic review, realist synthesis

Word count

3600

For peer review only - http://bmjopen.bmj.com/site/about/guidelines.xhtml

Abstract

Objective

Health services in many countries are investing in inter-organisational networks, linking patients' records held in different organisations across a city or region. The aim of the systematic review was to establish how, why, and in what circumstances these networks improve patient safety, fail to do so, or increase safety risks, for people living at home.

Design

Realist synthesis, including consultation with stakeholders in nominal groups and semi-structured interviews.

Eligibility criteria

The co-ordination of services for older people living at home, and medicine reconciliation for older patients returning home from hospital.

Information sources

17 sources including Medline, Embase, CINAHL, Cochrane Library, Web of Science, ACM Digital Library and Applied Social Sciences Index and Abstracts (ASSIA).

Outcomes

Changes in patients' clinical risks.

Results

We did not find any detailed accounts of the sequences of events that policy makers and others believe will lead from the deployment of interoperable networks to improved patient safety. We were, though, able to identify a substantial number of theory fragments, and these were used to develop programme theories.

For peer review only

There is good evidence that there are problems with the co-ordination of services in general, and the reconciliation of medication lists in particular, and it indicates that most problems are social and organisational in nature. There is also good evidence that doctors and other professionals find interoperable networks difficult to use. There was limited high quality evidence about safety-related outcomes associated with the deployment of interoperable networks.

Conclusions

Empirical evidence does not currently justify claims about the beneficial effects of interoperable networks on patient safety. There appears to be a mismatch between technology-driven assumptions about the effects of networks and the essentially social nature of co-ordination problems.

Review registration

PROSPERO CRD42017073004

For peer review only

For peer review only - http://bmjopen.bmj.com/site/about/guidelines.xhtml

Strengths and limitations of this study

- This is the first systematic review that seeks to explain the effects of extra-hospital information technology networks on patient safety
- The review investigates the distance between policy aspirations and realities in clinical settings
- We were only able to test a limited set of possible explanations for the effects of networks
- Aspects of quality appraisal rely on research team judgements: other teams might make different judgements
- Breadth of coverage was maximised, and to some extent traded off against the overall quality of included articles

Funding

National Institute for Health Research - Health Services and Delivery Research programme, project 16/53/03

Competing interests

None declared

For peer review only

Background

Many people who live in their own homes, and who have a number of health problems, need support from a range of professionals. There is good evidence that treatment and care is often fragmented, and increases patients' safety risks.[1, 2] Policy makers and opinion leaders have argued that interoperable networks, which give clinicians access to patient records held in health care organisations across cities and regions, can help to overcome the fragmentation.[Institute of Medicine, 2011 #570] They should, therefore, support safer treatment and care. The networks may, among other intended effects, support responses to the World Health Organisation's third Grand Challenge, to improve medication safety.[3]

The availability of interoperable networks has increased substantially in the course of the last decade. The networks reflect the history of the development of separate information technology (IT) systems in different organisations, each with its own data structures and interfaces. Evidence suggests that most are functionally (or foundationally) interoperable: clinicians in one organisation can access patient records on other organisations' servers, but the records themselves are not integrated, and presented to everyone in whatever format has been developed within each organisation.[4] They are often contrasted with more integrated networks, where records systems in different organisations are standardised, and clinicians have increasingly 'seamless' access to patient records. Systematic review evidence about the use of, and effects of, interoperable networks is relatively limited and mixed.[5, 6] The state of affairs can be contrasted with hospital-based systems and telemedicine applications, where the number of published studies is far larger, although many of those reviews emphasise problems with the quality of published evidence.[7, 8]

This article reports on the findings of a systematic review, using the realist synthesis method, of the effects of interoperable networks on patient safety. The method has been developed to investigate the sequences of decisions and actions that follow an intervention, as well as its effects. A range of quantitative and narrative evidence is typically included in a review. The effects of interoperable networks on resource use have been reviewed but not, to date, their effects on patient safety.[6] Further there is evidence, particularly for hospital systems, that information technologies can

For peer review only - http://bmjopen.bmj.com/site/about/guidelines.xhtml

increase as well as ameliorate patients' risks. There is value, then, in understanding how those risks might be managed.[9] The realist synthesis method allowed us to investigate both safety-related outcomes and the sequences of decisions and actions that led to those outcomes. Our searches investigated ways in which individual health professionals and clinical teams accessed and used networks, and influenced their working practices.[10-13]

The realist synthesis included structured database searches of a wide range of databases, supplementary searches and stakeholder consultation. The synthesis is reported according to the Preferred Reporting Items for Systematic Reviews and Meta-Analysis guidelines, and is consistent with RAMESES guidelines.[14, 15] A protocol was developed and submitted to the PROSPERO registry of systematic reviews prior to commencing the review (CRD42017073004).

Design

The realist synthesis was undertaken in two discrete stages, theory development and empirical assessment. Theory development involved the development of programme theories, these being representations of the way(s) in which an intervention is intended to work.[16-18] Structured database and complementary searches were undertaken to identify published theories, or fragments of theories, i.e. theories which covered a part of the sequences of decisions and actions that lead from the intervention to a safety-related outcome. Stakeholder consultation is usual in realist syntheses, and in this study nominal group consultation was used to refine the initial programme theories, and to help to identify appropriate populations and settings for assessment. Assessment was undertaken through structured database and complementary searches, designed to identify empirical evidence to establish whether programme theories worked in the ways that were intended - or did not work, or worked in some other way.

Theory development

Five theory development searches were undertaken, including structured subject searches, a review of England and USA government policies and official reports,

For peer review only - http://bmjopen.bmj.com/site/about/guidelines.xhtml

named author searches (for Professors David Bates and Robert Wachter), systematic review and a Web of Science 'usage count' (articles with a high level of interest) searches (see Figure 1). We were looking for statements that set out authors' reasoning about the effects of interoperable networks, which might be found in a wide range of texts, including editorials and interviews as well as journal articles and book chapters. They could be described using different terms, so we used very broad inclusion criteria and read substantial numbers of full texts. Passages where theories and fragments were described were copied into Word files, or hyperlinks created to long passages. The passages were synthesised into initial programme theories.[19]

Figure 1 about here

The initial theories, in the form of diagrams and supporting explanatory text, were presented to two nominal groups, of national policy makers and of information technology leads responsible for interoperable networks in two regions of England, and to a patient and public involvement (PPI) panel. The nominal group and PPI panel members commented on the face validity of the programme theories, suggested ways in which they might be refined, and indicated their priorities for evidence searches. Their comments fed into the identification and development of a main programme theory, and to inform the design of detailed evidence searches.

Patient and public involvement

The PPI panel met three times in the course of the review and contributed in the following ways:

- Their comments helped us to set priorities for the populations and settings used in the searches;
- They contributed to the interpretation of the initial findings of the evidence searches;
- They commented on our overall interpretation of our findings at the end of the review.

For peer review only - http://bmjopen.bmj.com/site/about/guidelines.xhtml

This being a literature review, patients were not recruited to this study. Summaries and full reports of the review have been sent to the nominal and PPI groups.

Evidence searches: data sources and searches

Searches were designed to interrogate the programme theory for two different functions of interoperable networks – the co-ordination of services for older people living in their own homes who were in receipt of two or more services from different organisations and, more specifically, the co-ordination of services for older people living in their own homes who had been prescribed medication from two or more organisations. (We focused on medicine reconciliation involving two or more lists of medications, and lists that were reconciled with a patient assessment undertaken by a clinician.) We undertook three searches for each function, focusing on the nature and extent of co-ordination problems (that interoperable networks might in principle help to address), on users' experiences of using interoperable networks, and on outcomes (defined as changes in patients' risks of harm).

The designs of the searches for each function were similar, allowing us to compare and contrast findings. All searches included the Medline database bundle 'Ovid MEDLINE® and Epub Ahead of Print, In-Process & Other Non-Indexed Citations and Daily 1946-present'.[15] (Details of additional databases used in each search, and PRISMA flow diagrams are provided in separate files.) Search terms and synonyms were identified by the project team on the basis of a programme theory selected for assessment (see Figure 2). All of the searches were performed and peer-reviewed by information specialists (NK, JW). Structured search strategies were developing using free text words, synonyms and subject index terms organised into search concepts. Further complementary searches, including forward and backward citation searches, were also undertaken.

Figure 2 about here

For peer review only - http://bmjopen.bmj.com/site/about/guidelines.xhtml

Study selection

Inclusion and exclusion criteria were designed for each search, with the following inclusion criteria common to all searches:

- Written in the English language;
- Published in 2000 or later.[20]

Articles were assessed for relevance and rigour by three reviewers (MA, JG, JK). Relevance was assessed pragmatically using a 'target', akin to an archery target. Articles that met all inclusion criteria were placed in the 'bullseye'. Those that met only partially met the population (e.g. frail older people) criteria, but met other criteria, were placed in the middle ring. Articles that did not strictly meet either population or intervention criteria, but where it was judged that they might nevertheless shed light on the mechanisms involved, were placed in the outer ring.

Data extraction and quality assessment

For included articles, study identifiers (author, publication year, country), information about the intervention and study methods (methods used, numbers and types of participants), and the evidence itself were recorded in customised spreadsheets. In relation to rigour, critical appraisal skills programme (CASP) quality assessment checklists were used to appraise systematic reviews, narrative and cohort studies.

Synthesis

The empirical evidence was used to review the initial programme theory. One of the distinguishing features of realist synthesis is that it combines evidence and reasoning, seeking to identify a line of reasoning that offers the best explanation for the available evidence.[21] Following Bunn and colleagues we interpreted the evidence in two ways, organising the evidence into a sequence of events and comparing it to the sequence in the initial programme theory, and drawing on a leading theory of IT implementation in doing so.[22, 23]

For peer review only

For peer review only - http://bmjopen.bmj.com/site/about/guidelines.xhtml

Results

Following study selection and assessment 46 studies were included. Many general statements were found which stated that interoperable networks would improve patient safety, but we did not find any detailed accounts that described or explained how they would produce safer diagnosis, treatment and care. A substantial number of theory fragments were, though, identified.[6, 24-27] For example, it was argued that interoperable networks would make information available to clinicians wherever and whenever it was needed, enabling integrated workflow and clinical decision support.[28] There were also statements about challenges and risks, associated with a lack of common data standards, problems with interfaces and concerns about privacy.[19] These and other fragments were combined to create an initial programme theory.

The nominal groups indicated that, rather than fine-grained analyses of programme theories, they would value evidence about effects on patient safety, and quality of treatment and care more generally. They agreed that semantic interoperability was desirable in principle. In practice, though, there was little incentive to provide anything beyond functional – basic - interoperability as many clinicians only used networks 'tactically', when they really needed information. As a result, evidence about functional interoperability would be valuable to them. They also indicated that their priorities for evidence searches, rating the co-ordination of services for frail elderly people living at home highest. The study patient and public involvement panel supported this priority. Therefore, we settled on a single programme theory for assessment, and exploration of two functions of interoperable networks – in co-ordinating care across professional and organisational boundaries, and more specifically in medicine reconciliation (see Figure 2).

Evidence searches: care co-ordination

Five reviews were included on the nature of co-ordination problems in services for older people (Table 1). The hand search of King's Fund reports produced two further reviews. The reviews were conducted in different academic traditions, and used different review methods, but produced broadly similar findings. There was good evidence that co-ordination problems were social and organisational in nature.

For peer review only - http://bmjopen.bmj.com/site/about/guidelines.xhtml

Leadership, organisation cultures and trust influenced the effectiveness of co-ordination. Communication problems were also highlighted. These were typically characterised as a combination of task failures – such as failures to pass key information about a patient to a clinical colleague – and problems with conveying messages to colleagues with different professional backgrounds. Information technologies were rarely mentioned as having a role in either creating or solving problems.

For peer review only

Table 1[2, 29-40]

The nature of co-ordination and of medicine reconciliation problems

Author and year	*Country*	*Method*	*Topic*	*Data type*	*Rigour*	*Relevance*	*Processes*	*Outcomes/errors*
Co-ordination								
Auschra (2018)**[29]**	-	Systematic review	Barriers to integrated care	Qualitative	**1**	1	Range of inter-personal and institutional issues	-
Threapleton et al (2017)**[2]**	-	Scoping review	Barriers & facilitators to co-ordination	Quantitative (E&O) and qualitative	2	1	Organisational and cultural features of co-ordination	-
Allen et al (2017)**[30]**	-	Narrative review	Transition from hospital to home	Qualitative	1	1	Negotiation and navigation of service user independence	-
Kirst et al (2017)**[31]**	-	Realist review	Conditions for effective team integration	Quantitative (E&O) and qualitative	1	1	Service use, patient and provider experience	Patient health status
Hudson et al (2014)[32]	-	Narrative review	Transition from hospital to home	Quantitative (O) and qualitative	2	3	Range of organisational and inter-professional issues	Re-admission rates, user satisfaction with transition
King's Fund (2014)**[33]**	7 countries	Synthesis of case study evidence	Models of integrated care	Qualitative	2	1	Range including flexibility of team working, effective communication, focus on users' needs	Effective integration associated with improved user satisfaction
King's Fund (2013)**[34]**	England	Multi-site case study	Evaluation of care co-ordination programmes	Qualitative	2	1	Range including organisational models, team cultures, engagement	-
Medicine reconciliation								
Godfrey et al (2013)[41]	-	Scoping review	Medication management	Quantitative (E&O) and qualitative	2	1	Include time costs of & responsibility for reconciliation, communication problems	Polypharmacy, potentially inappropriate prescribing
Tommelein et al (2015)[42]	Europe	Systematic prevalence survey	Potentially inappropriate prescribing	Quantitative (O)	2	3	-	Polypharmacy, patient characteristics including advanced age
Hernandez (2017)[43]	USA	Interviews (commu	Co-ordination of services	Qualitative	1	1	Inter-professional co-ordination, communication problems	Polypharmacy, medication errors, adverse events

For peer review only - http://bmjopen.bmj.com/site/about/guidelines.xhtml

		nity nurses)						
Kennelly et al (2015)[44]	USA	Interviews (pharmacists)	Reconciliation post hospital discharge	Qualitative	1	1	Resources, communication, inter-professional relationships	-

For peer review only

Table 2

Co-ordination of services: users' experiences of interoperable networks

Authors/year	*Country*	*Methods*	*Topic*	*Rigour*	*Rel*
Eden (2016)**[35]**	-	Systematic review	HIE barriers and faciliators	1	3
Azarm-Daigle et al (2015) **[36]**	-	Systematic review	Cross-organisational data sharing	1	2
Hoerbst and Schwitzer 2015[37]	-	Systematic review	Critical success factors for clinical information systems in integrated care	1	2
Wu et al (2015)**[38]**	USA	Systematic review	HIE barriers and facilitators	1	3
Nicolaisen and Berg (2015) **[39]**	Norway	Primary qualitative: interviews	Perceptions of messaging system	1	2
McMurray et al (2013)[40]	Canada	Primary qualitative: ethnographic study	Impact of partial interoperability	1	1

For peer review only

We included four reviews and two primary studies from the evidence searches for user experiences of interoperable networks in the adult and older population (rather than older people specifically, Table 2). A forward citation search from a 2013 review of the computer-supported cooperative work literature did not yield any further articles.[13] Most evidence was based on interviews, and therefore on subjective reports of behaviour rather than direct observational evidence of users' experiences. Key details were missing from most reports, including information about interface characteristics and other features of the interoperable networks studied. This said, there was a consistent theme, indicating that interoperable networks were difficult to use, with problems reported in accessing networks, searching for and finding relevant information, and hence perceptions that the time costs of these activities were excessive.

Three articles were included from the searches for evidence about patient outcomes. All three were based on interviews with small samples of interviewees, reporting on experiences with networks with limited functionality. There was a common picture of difficulties encountered in accessing and using them, which led to broadly negative perceptions of their value.[45-47] Five review articles were identified as relevant in a subsequent broader search, for evidence about adult patients in general (Table 3). They included quantitative evidence, of variable quality, about intermediate measures including adverse event and hospital re-admission rates. The results were, broadly, positive in the sense that population rates were reported to reduce, implying the possibility of reductions in patients' risks. However, there were also some negative results, and none of the reviews included any quantitative evidence about the effects of interoperable networks on patient outcomes.

For peer review only

Table 3[26, 45-51]

Co-ordination of services: service and patient outcomes

Authors/year	*Country*	*Methods*	*Topic*	*Rigour*	*Relevance*
King et al (2012)[47]	Scotland	Interviews	Electronic shared assessment tool	1	3
Waterson et al (2012)[46]	England	Interviews, observation of meetings	E-health supported care pathway	2	3
Vimarlund et al (2008)[45]	Sweden	Interviews	Virtual health record tool	3	3
Health Quality Ontario (2013)[48]	-	Systematic review	e-tools, HIE and care co-ordination	1	2
Sadough et al (2018)[49]	-	Systematic review	HIE, quality of care	1	2
Hersh et al (2015)[26]	-	Systematic review	Effectiveness of HIE	1	2
Reis et al (2017)[50]	-	Review of systematic reviews	Cost-benefit of records, HIE, inter-operability	1	2
Menachemi et al (2018)[51]	-	Systematic review	HIE and service changes	1	2

Evidence searches: medicine reconciliation

For the nature of medicine reconciliation problems question, one systematic review, one other review and two observational studies were included (Table 1). The overall quality of empirical evidence was reasonable. It indicated that the challenges were social and organisational in nature, the main one being that responsibility for reconciliation is not clear on the ground, particularly following discharge from hospital to home. As a result, responsibility fell between professionals, principally pharmacists, doctors and nurses. Reconciliation could also be viewed as an administrative task (rather than a safety-promoting one), and was not deemed to be important by some professionals. Communication problems emerged clearly as a theme.

Nine articles were identified that shed light on user experiences of medicine reconciliation. [44, 52-59] These were a mix of scenario-based and field-based observational studies. The search for evidence about patient outcomes did not identify any quantitative evidence about changes in patients' risks. Results about proxy outcomes – changes in reconciliation error rates - were mixed. Some articles

For peer review only - http://bmjopen.bmj.com/site/about/guidelines.xhtml

indicated that use of an interoperable network was associated with a measurable reduction in reconciliation errors. Others reported problems with using systems, resulting in no effects on error rates.

Discussion

We did not find any detailed published accounts that described the ways in which interoperable networks might improve patient safety (or increase patients' risks). That is, we did not find evidence that policy makers and opinion leaders have thought through the logic of their assumptions about interoperable networks. Further, we found insufficient evidence to allow us to establish whether, how or why interoperable networks affect patient safety outcomes. The assumption that clinicians will easily access networks, and find and use relevant patient information, is not supported. Overall, the programme theory is neither supported nor rejected. However, users' negative experiences raise doubts about proponents' beliefs about the effectiveness of interoperable networks.

There were two main limitations to the study. First, we did not test alternative explanations for the effects of interoperable networks on patient safety: it is not possible to rule out plausible explanations that have not been considered by policy makers and others. Second, aspects of the method involved team judgements: other research teams might, for example, adopt different criteria for assessing rigour and relevance. It seems reasonable to hope that their results would be broadly similar to ours, but we cannot be sure of this. These points made, the findings are less positive than findings about the effects of IT systems on patient safety hospital settings, notably in relation to service outcomes associated with medicine reconciliation in hospital settings.[24] They are consistent with reports of poor user experiences with IT systems in other settings.[60, 61]

The theory fragments were drawn from a range of sources, and point to a seemingly widely held technology-driven line of reasoning. It starts with the argument that clinicians need access to information held in remotely held patient records. When they have access, their decision-making will improve and patients' clinical risks will be reduced. Some observers argue that current IT solutions are not very good, but

For peer review only

when technologies improve then improvements in treatment and outcomes will follow.[62, 63] The evidence indicates that these arguments are misplaced. There is good evidence that the co-ordination of services is socially rather than technologically driven: clinicians need to communicate with and understand one another, and at times negotiate with one another, to identify the best course of action for a patient. There will sometimes be genuine differences of opinion, and situations where professionals do not trust one another. In these circumstances leadership and management are important - and inherently difficult.[64, 65] Changing the characteristics of an IT network – when and if the IT solutions improve in some way - will not necessarily, therefore, lead to improvements in working practices or to safer patient care.

As far as we are able to tell, given sometimes limited information about interventions, all of the reported studies were conducted on functional networks, that allowed users to access other organisations' record systems, but were not integrated with one another. Users will have had to interrogate each system separately. As noted above, we only found reports of difficulties. Given the nature of the networks this is not a surprise, but it is not clear that this point has been given serious consideration in policy and other circles.

We conclude that policy makers and other stakeholders, including clinicians and suppliers, should examine the mechanistic assumptions that underpin current thinking. They should consider focusing on the everyday organisational realities of working across professional and organisational boundaries, and ways in which networks might make it easier for busy clinicians to co-ordinate their work with one another. Looking ahead, these findings only deepen the puzzle about the quality of IT solutions available to clinicians: there is a need to understand why this continues to be the case. To this end, we were struck by the absence of detailed accounts of the effects of interoperable networks in policy and other documents: there is therefore value in critically examining the assumptions made by policy makers and other proponents of these networks. In addition, we in common with other authors believe that primary field studies of users' experiences of interoperable networks, to identify solutions more acceptable to clinicians, are needed.[13]

For peer review only - http://bmjopen.bmj.com/site/about/guidelines.xhtml

For peer review only

For peer review only - http://bmjopen.bmj.com/site/about/guidelines.xhtml

Authors' contributions

Peter Gardner, Joanne Greenhalgh, Justin Keen, Roberta Longo, Rebecca Randell, Justin Waring and Judy Wright developed the proposal for the study. Natalie King and Judy Wright designed and undertook structured database searches. Maysam Abdulwahid, Joanne Greenhalgh and Justin Keen undertook screening and data extraction. Peter Gardner and Justin Waring provided specialist input to the design and interpretation of specific searches. All authors contributed to the detailed study design and to the interpretation of overall findings. All authors either drafted, or commented on drafts of, this article.

Acknowledgements

We are grateful to the members of the nominal groups, and the PPI panel, for their contributions to the design and conduct of the review.

Data sharing statement

The majority of material in this article is derived from already-published articles and reports. Beyond this, other methods were qualitative and the data generated is not suitable for sharing. Further information can be obtained from the corresponding author.

Checklist

PRISMA checklist uploaded with this article.

Patient consent form

No patients were recruited to this study.

Supplementary data

Separate files are provided – a PRISMA checklist, one file for each Figure, and file with full details of searches undertaken and PRISMA flow diagrams.

For peer review only

For peer review only

For peer review only - http://bmjopen.bmj.com/site/about/guidelines.xhtml

References

1. Stocker, R., et al., *Care home services at the vanguard: a qualitative study exploring stakeholder views on the development and evaluation of novel, integrated approaches to enhancing healthcare in care homes.* BMJ Open, 2018. **8**(3): p. e017419.
2. Threapleton, D.E., et al., *Integrated care for older populations and its implementation facilitators and barriers: A rapid scoping review.* International Journal for Quality in Health Care, 2017. **29**(3): p. 327-334.
3. World Health Organisation, *Medication without harm*. 2017.
4. Devine, E.B., et al., *Health Information Exchange Use (1990-2015): A Systematic Review.* EGEMS (Wash DC), 2017. **5**(1): p. 27.
5. Bowden, T. and E. Coiera, *The role and benefits of accessing primary care patient records during unscheduled care: a systematic review.* BMC Medical Informatics & Decision Making, 2017. **17**(1): p. 138.
6. Rudin, R.S., et al., *Usage and effect of health information exchange: a systematic review.* Ann Intern Med, 2014. **161**(11): p. 803-11.
7. Black AD, et al., *The impact of ehealth on the quality and safety of healthcare*, in *PLOS MED*. 2011.
8. Brenner, S.K., et al., *Effects of health information technology on patient outcomes: a systematic review.* J Am Med Inform Assoc, 2016. **23**(5): p. 1016-36.
9. Institute of Medicine, *Health IT and patient safety: Building Safer Systems for Better Care*. 2011, Washington (DC): National Academies Press (US).
10. Andreassen, H.K., L.E. Kjekshus, and A. Tjora, *Survival of the project: A case study of ICT innovation in health care.* Social Science & Medicine, 2015. **132**: p. 62-69.
11. Greenhalgh T, W.J., Papoutsi C, Lynch J, Hughes G, A'Court C, Hinder S, Fahy N, Procter R, Shaw S, *Beyond Adoption: A New Framework for Theorizing and Evaluating Nonadoption, Abandonment, and Challenges to the Scale-Up, Spread, and Sustainability of Health and Care Technologies.* JMIR. **19**: p. e367.
12. Williams, R., *Why is it difficult to achieve e-health systems at scale?* Information, Communication & Society, 2016. **19**(4): p. 540-550.
13. Fitzpatrick, G. and G. Ellingsen, *A Review of 25 Years of CSCW Research in Healthcare: Contributions, Challenges and Future Agendas.* Computer Supported Cooperative Work (CSCW), 2013. **22**(4-6): p. 609-665.
14. Geoff Wong, et al., *RAMESES publication standards:meta-narrative reviews.* BMC medicine, 2013. **11**(20): p. 1-15.
15. EUNETHTA, *Guideline: Process of information retrieval for systematic reviews and health technology assessments on clinical effectiveness. Version 1.2.* , E.N.F.H.T. ASSESSMENT, Editor. 2017
, Institute for Quality and Efficiency in Health Care Germany.
16. Pawson, R., *Evidence-based policy*. 2006, London: SAGE Publications.
17. Bunn, F., et al., *Supporting shared decision making for older people with multiple health and social care needs: a realist synthesis.* BMC Geriatrics, 2018. **18**(1): p. 165.

For peer review only - http://bmjopen.bmj.com/site/about/guidelines.xhtml

18. Greenhalgh, T., et al., *Protocol - realist and meta-narrative evidence synthesis: Evolving Standards (RAMESES).* Bmc Medical Research Methodology, 2011. **11**.
19. Shearn, K., et al., *Building Realist Program Theory for Large Complex and Messy Interventions.* International Journal of Qualitative Methods, 2017. **16**(1): p. 1609406917741796.
20. *To Err is Human : Building safer healthcare system* in *Quality of Health Care in America*. 1999, Institue of Medicine USA.
21. Best, A., et al., *Large-System Transformation in Health Care: A Realist Review.* The Milbank Quarterly, 2012. **90**(3): p. 421-456.
22. Bunn, F., et al., *Managing diabetes in people with dementia: a realist review.* Health Technology Assessment (Winchester, England), 2017. **21**(75): p. 1-140.
23. Pollock M, W.R., *Software and Organisations*. 2008, London: Routledge.
24. Marien, S., B. Krug, and A. Spinewine, *Electronic tools to support medication reconciliation: a systematic review.* J Am Med Inform Assoc, 2017. **24**(1): p. 227-240.
25. Bassi, J., F. Lau, and S. Bardal, *Use of information technology in medication reconciliation: a scoping review.* Annals of Pharmacotherapy, 2010. **44**(5): p. 885-97.
26. Hersh, W.R., et al., *Outcomes From Health Information Exchange: Systematic Review and Future Research Needs.* JMIR Med Inform, 2015. **3**(4): p. e39.
27. Zheng, K., et al., *A Survey of the Literature on Unintended Consequences Associated with Health Information Technology: 2014-2015.* Yearbook of Medical Informatics, 2016(1): p. 13-29.
28. Wachter, R.M., *Making IT work : harnessing the power of health information technology to improve care in England : report of the National Advisory Group on Health Information Technology in England*. 2016, London: Department of Health.
29. Auschra, C., *Barriers to the Integration of Care in Inter-Organisational Settings: A Literature Review.* Int J Integr Care, 2018. **18**(1): p. 5.
30. Allen, J., et al., *User experience and care integration in transitional care for older people from hospital to home: A meta-synthesis.* Qualitative Health Research, 2017. **27**(1): p. 24-36.
31. Kirst, M., et al., *What works in implementation of integrated care programs for older adults with complex needs? A realist review.* International Journal for Quality in Health Care, 2017. **29**(5): p. 612-624.
32. Hudson, R., L. Comer, and R. Whichello, *Transitions in a wicked environment.* Journal of Nursing Management, 2014. **22**(2): p. 201-210.
33. Goodwin, N., et al., *Providing integrated care for older people with complex needs Lessons from seven international case studies*. 2014.
34. Goodwin, N., et al., *Co-ordinated care for people with complex chronic conditions.* The Kings Fund, 2013.
35. Eden, K.B., et al., *Barriers and facilitators to exchanging health information: a systematic review.* International journal of medical informatics, 2016. **88**: p. 44-51.
36. Azarm-Daigle, M., C. Kuziemsky, and L. Peyton, *A Review of Cross Organizational Healthcare Data Sharing.* Procedia Computer Science, 2015. **63**: p. 425-32.

For peer review only

For peer review only - http://bmjopen.bmj.com/site/about/guidelines.xhtml

37. Hoerbst, A. and M.L. Schweizer, *A Systematic Investigation on Barriers and Critical Success Factors for Clinical Information Systems (CIS) in Integrated Care Settings.* Yearb Med Inform, 2015. **10**(1): p. 79-89.
38. Wu, H. and E. Larue, *Barriers and Facilitators of Health Information Exchange (HIE) Adoption in the United States*, in *Proceedings of the 2015 48th Hawaii International Conference on System Sciences*. 2015, IEEE Computer Society.
39. Nicolaisen, K. and K. Berg, *Electronic communication across organizational borders in healthcare: an empirical study*. 2015, UiT Norges arktiske universitet.
40. McMurray, R., et al., *Managing controversy through consultation: A qualitative study of communication and trust around MMR vaccination decisions.* British Journal of General Practice, 2004. **54**(504): p. 520-525.
41. Godfrey, C.M., et al., *Homecare safety and medication management with older adults: A scoping review of the quantitative and qualitative evidence.* JBI Database of Systematic Reviews and Implementation Reports, 2013. **11**(7): p. 82-130.
42. Tommelein, E., et al., *Potentially inappropriate prescribing in community-dwelling older people across Europe: a systematic literature review.* European Journal of Clinical Pharmacology, 2015. **71**(12): p. 1415-27.
43. Hernandez, J., *Medication management in the older adult: A narrative exploration.* Journal of the American Association of Nurse Practitioners, 2017. **29**(4): p. 186-194.
44. Kennelty, K.A., et al., *Barriers and facilitators of medication reconciliation processes for recently discharged patients from community pharmacists' perspectives.* Research in Social & Administrative Pharmacy, 2015. **11**(4): p. 517-530.
45. Vimarlund, V., et al., *Organizational effects of information and communication technology (ICT) in elderly homecare: a case study.* Health Informatics Journal, 2008. **14**(3): p. 195-210.
46. Waterson, P., et al., *Using HIT to deliver integrated care for the frail elderly in the UK: current barriers and future challenges.* Work, 2012. **41**: p. 4490-4493.
47. King, G., et al., *Boundaries and e-health implementation in health and social care.* BMC Medical Informatics & Decision Making, 2012. **12**: p. 100.
48. Health Quality Ontario, *Electronic tools for health information exchange: an evidence-based analysis.* Ont Health Technol Assess Ser, 2013. **13**(11): p. 1-76.
49. Sadoughi, F., S. Nasiri, and H. Ahmadi, *The impact of health information exchange on healthcare quality and cost-effectiveness: A systematic literature review.* Computer Methods and Programs in Biomedicine, 2018. **161**: p. 209-32.
50. Reis, Z.S.N., et al., *Is There Evidence of Cost Benefits of Electronic Medical Records, Standards, or Interoperability in Hospital Information Systems? Overview of Systematic Reviews.* JMIR Medical Informatics, 2017. **5**(3): p. e26.
51. Menachemi, N., et al., *The benefits of health information exchange: an updated systematic review.* J Am Med Inform Assoc, 2018.
52. Horsky, J., E.A. Drucker, and H.Z. Ramelson, *Higher accuracy of complex medication reconciliation through improved design of electronic tools.* Journal of the American Medical Informatics Association, 2018. **25**(5): p. 465-475.

For peer review only - http://bmjopen.bmj.com/site/about/guidelines.xhtml

53. Bitan, Y., et al., *The Cognitive Task of Medication Reconciliation - Clinicians' Approaches to the Arrangement of Medical Condition and Medication History Information.* Proceedings of the Human Factors and Ergonomics Society Annual Meeting, 2016. **60**(1): p. 538-540.
54. van Stiphout, F., et al., *Task analysis of information technology-mediated medication management in outpatient care.* British Journal of Clinical Pharmacology, 2015. **80**(3): p. 415-24.
55. Vashitz, G., et al., *How do clinicians reconcile conditions and medications? The cognitive context of medication reconciliation.* Cognition, Technology & Work, 2013. **15**(1): p. 109-116.
56. Vashitz, G., et al., *Making sense of diseases in medication reconciliation.* Cognition, Technology & Work, 2011. **13**(2): p. 151-158.
57. Foged, S., et al., *Nurses' perspectives on how an e-message system supports cross-sectoral communication in relation to medication administration: A qualitative study.* Journal of Clinical Nursing, 2018. **27**(3-4): p. 795-806.
58. Fanizza, F.A., et al., *Integrating a health information exchange into a community pharmacy transitions of care service.* J Am Pharm Assoc (2003), 2018. **58**(4): p. 442-449.
59. Hohmeier, K.C., et al., *Implementation of a health information exchange into community pharmacy workflow.* J Am Pharm Assoc (2003), 2017. **57**(5): p. 608-615.
60. *What It Will Take To Achieve The As-Yet-Unfulfilled Promises Of Health Information Technology.* Health Affairs, 2013. **32**(1): p. 63-68.
61. GOLD, M. and C. McLAUGHLIN, *Assessing HITECH Implementation and Lessons: 5 Years Later.* The Milbank Quarterly, 2016. **94**(3): p. 654-687.
62. Wachter, R., *The digital doctor : hope, hype, and harm at the dawn of medicine's computer age*. 2015, New York: McGraw Hill Education. xv, 1, 330.
63. Gawande, A., *Why doctors hate their computers*, in *New Yorker*. 2018: New York.
64. Mannion, R. and H. Davies, *Understanding organisational culture for healthcare quality improvement.* BMJ, 2018. **363**: p. k4907.
65. Exworthy, M., M. Powell, and J. Glasby, *The governance of integrated health and social care in England since 2010: great expectations not met once again?* Health Policy, 2017. **121**(11): p. 1124-1130.

For peer review only

Figure 1
Theory Development PRISMA diagram

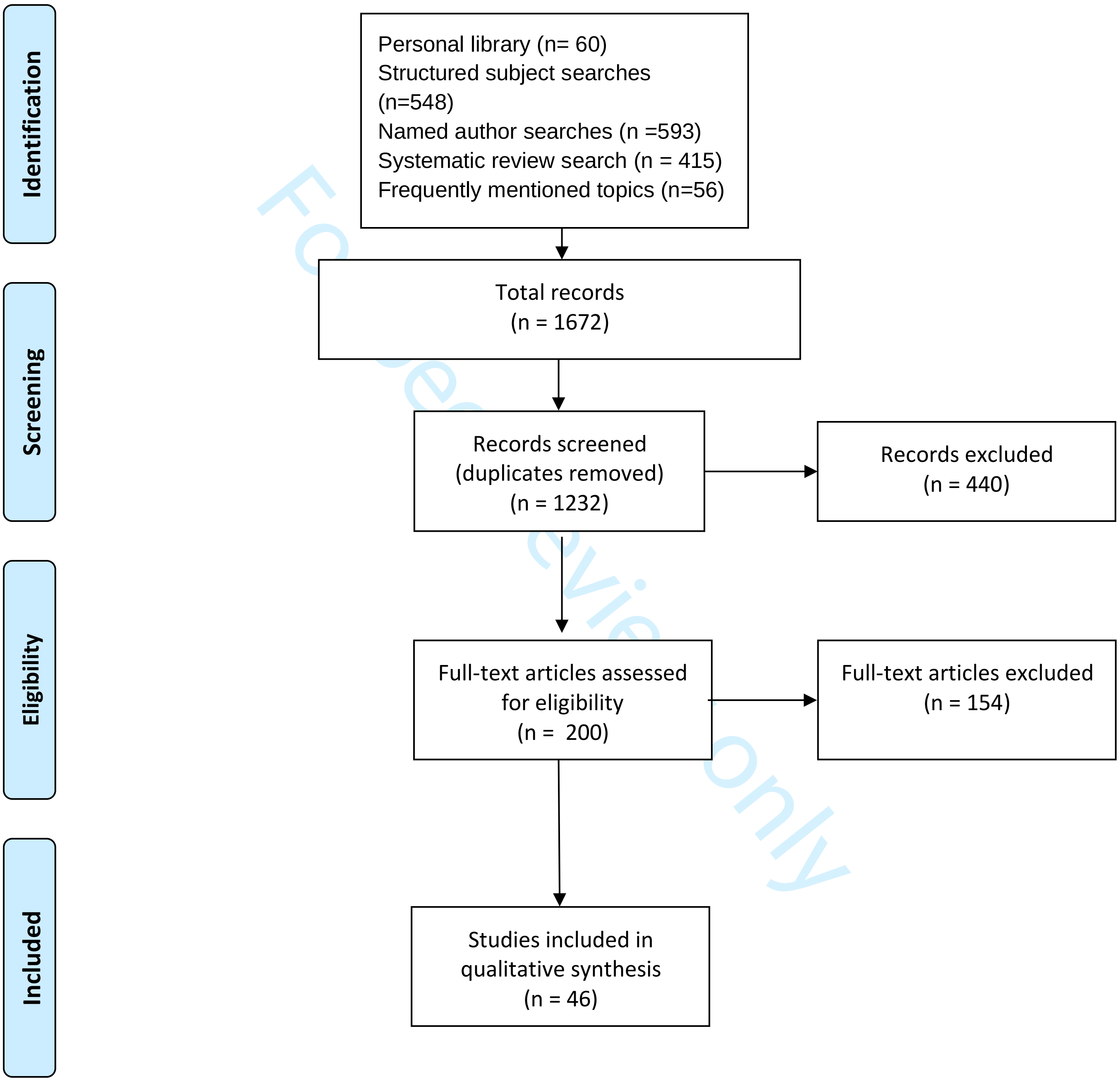

Figure 2: Main programme theory

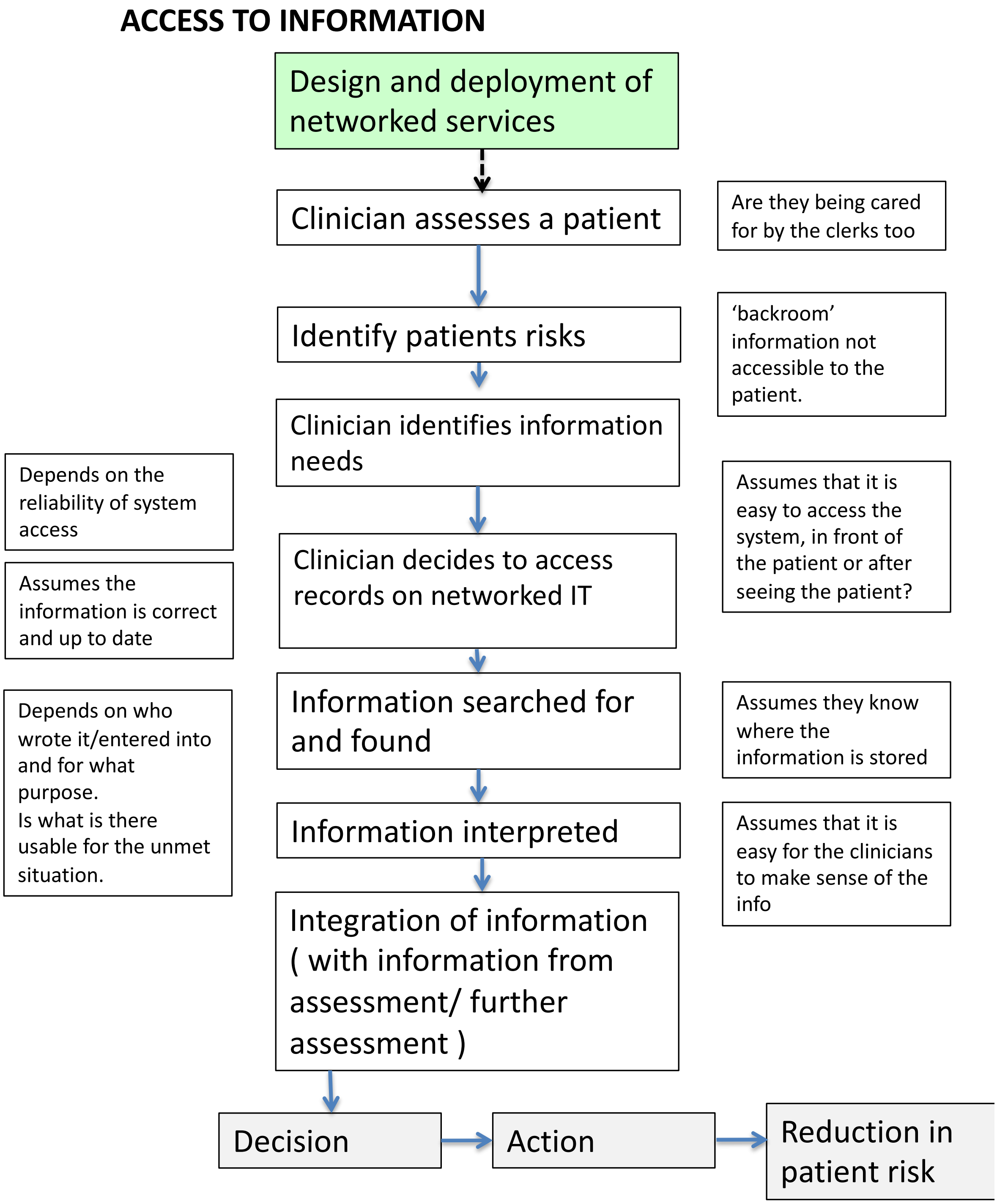

SUPPLEMENTARY FILE – PRISMA FLOW DIAGRAMS AND SEARCH STRATEGIES

Figure A

PRISMA diagram: care co-ordination, nature of the problem

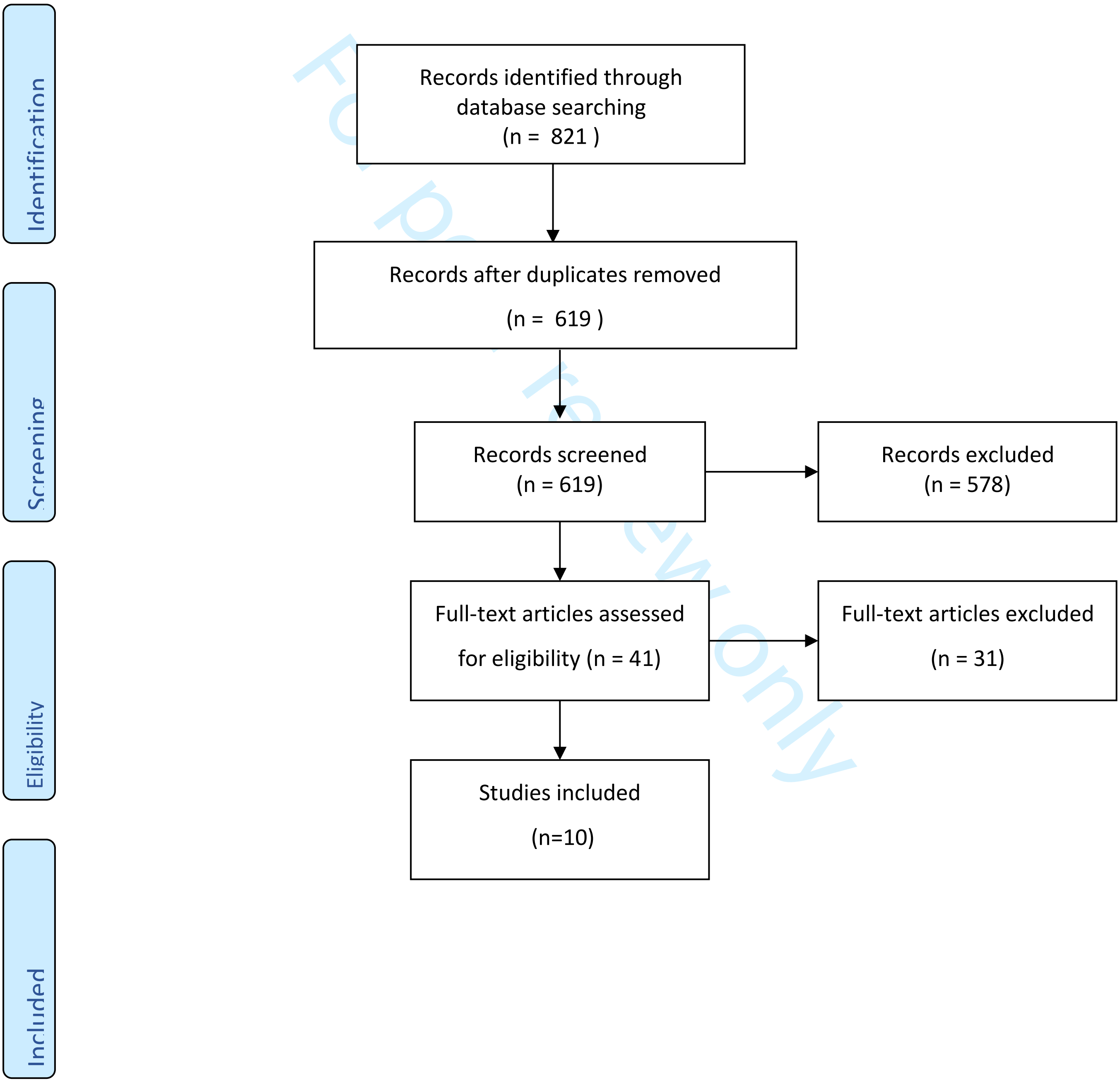

Figure B

PRISMA diagram: care co-ordination, users' experiences

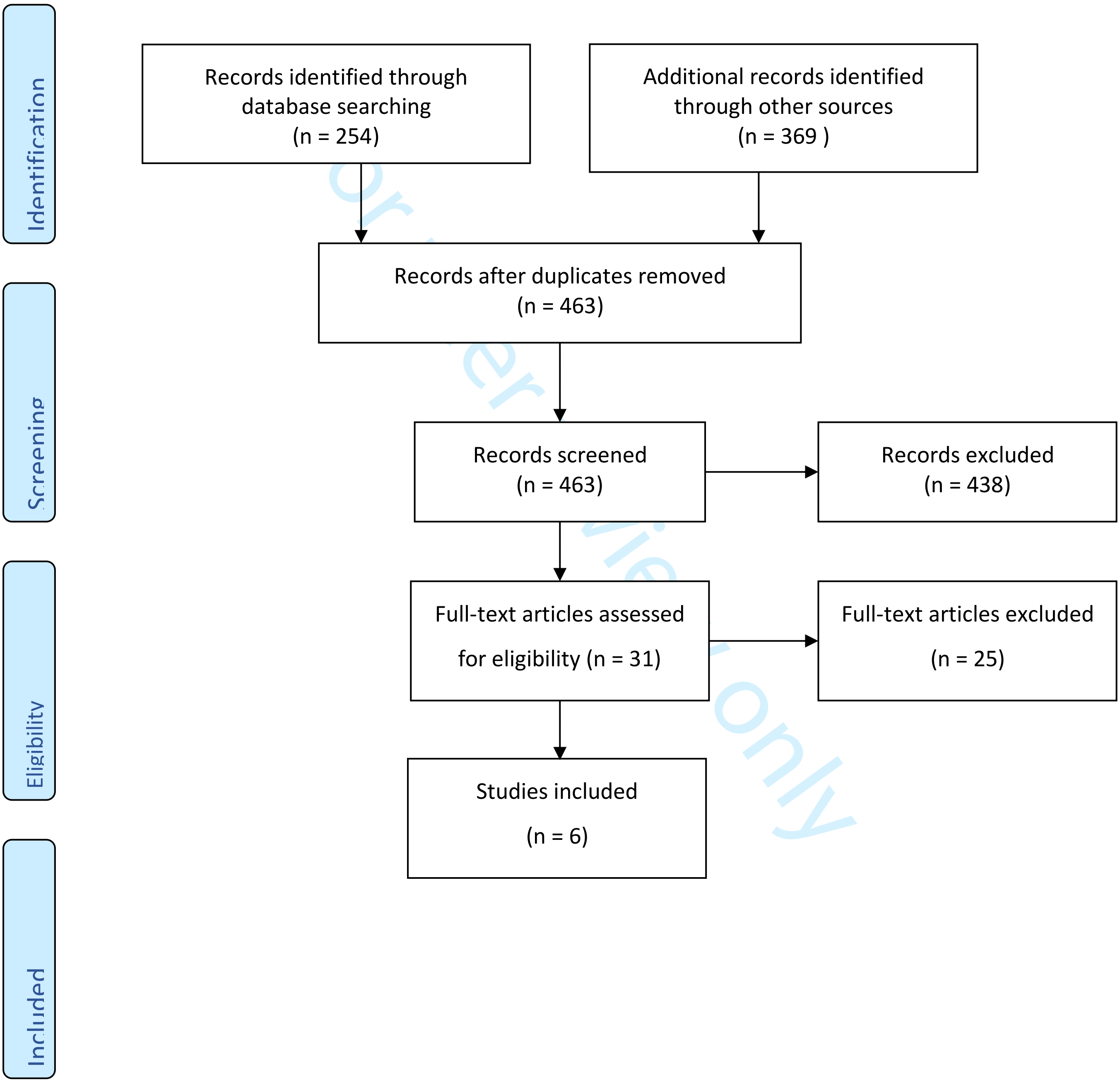

Figure C

PRISMA diagram: care co-ordination, outcomes

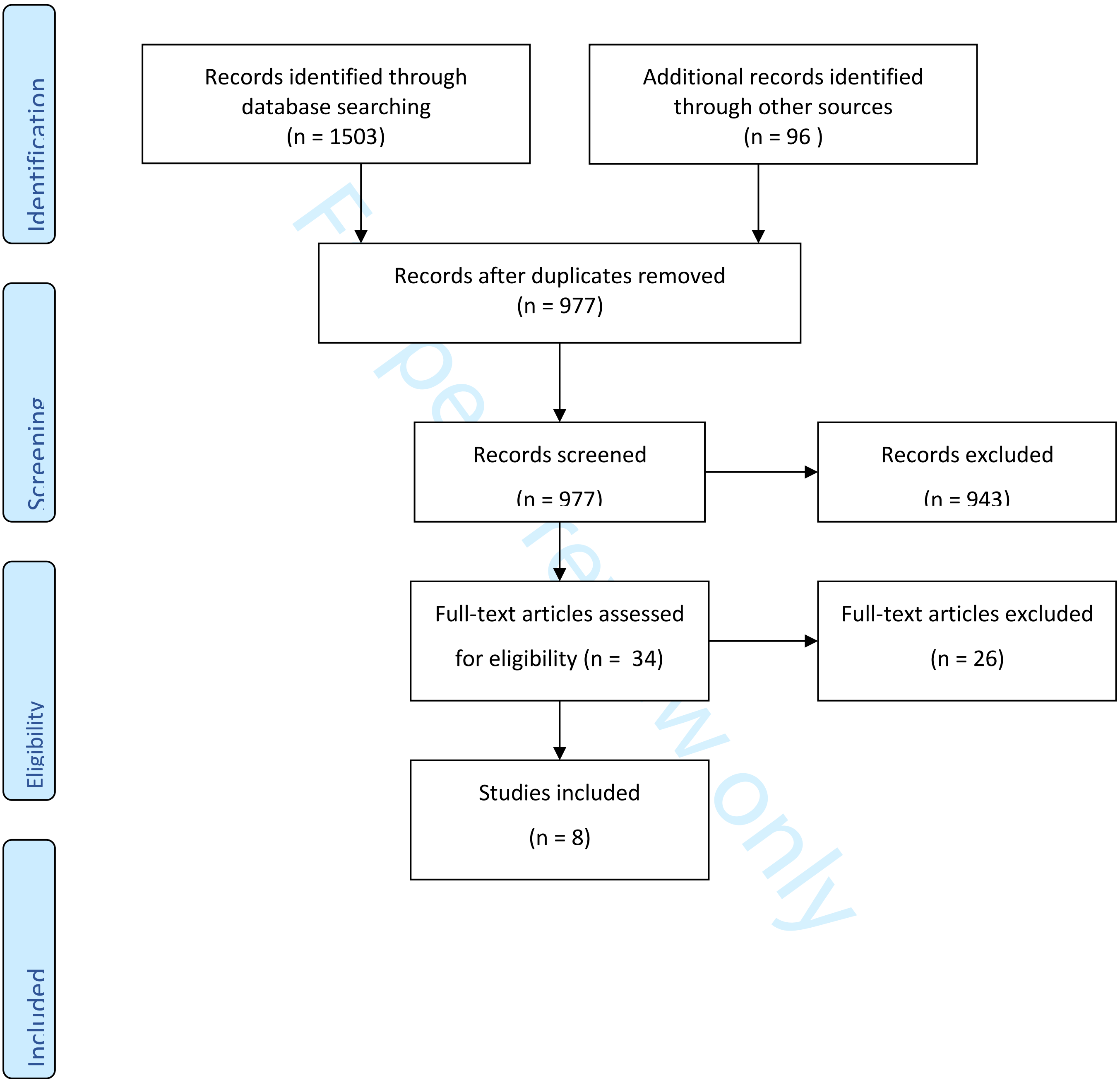

Figure D

PRISMA diagram: medicine reconciliation – nature of the problem

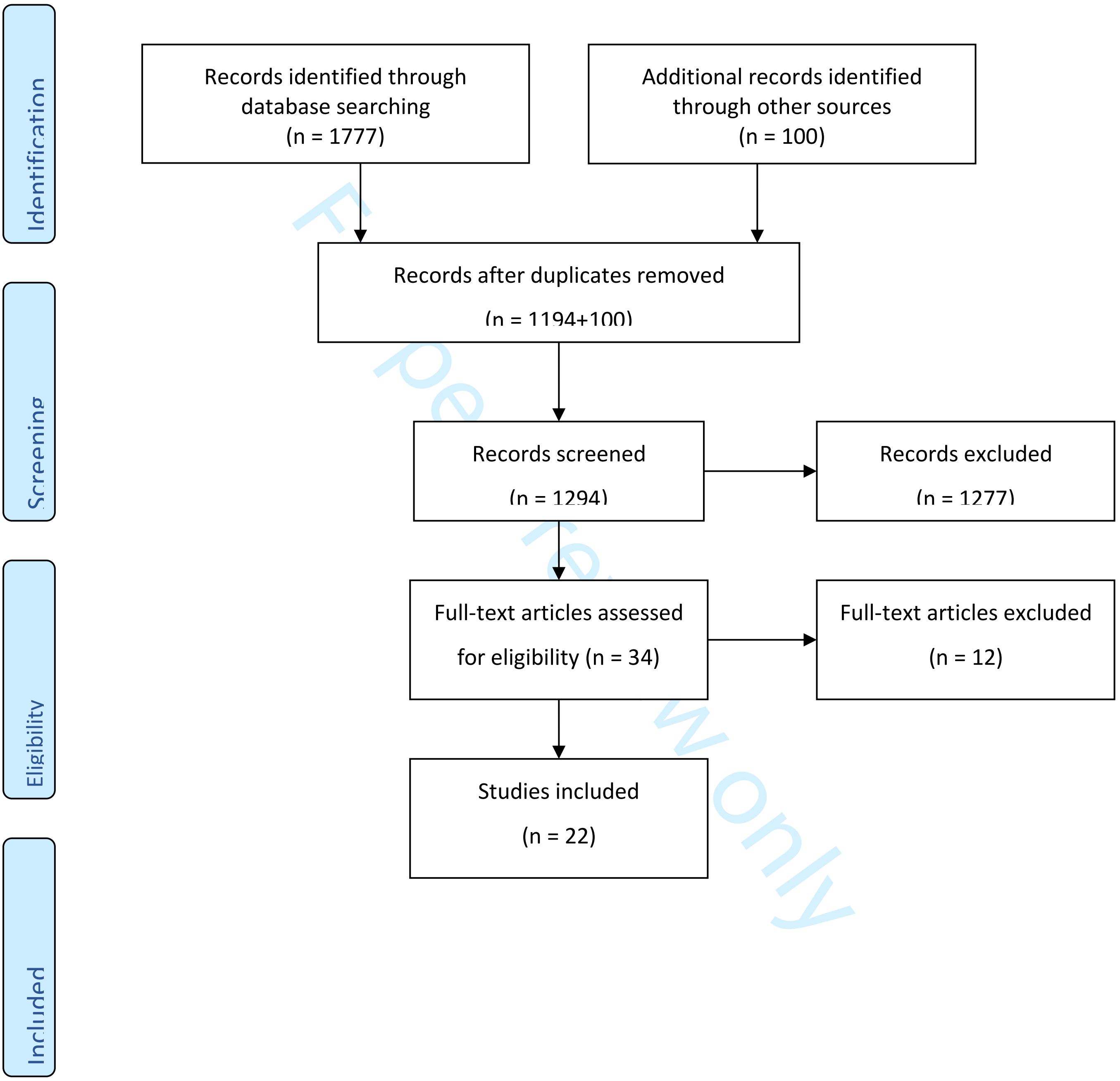

For peer review only - http://bmjopen.bmj.com/site/about/guidelines.xhtml

For peer review only

Figure E

PRISMA diagram: medicine reconciliation – users' experiences

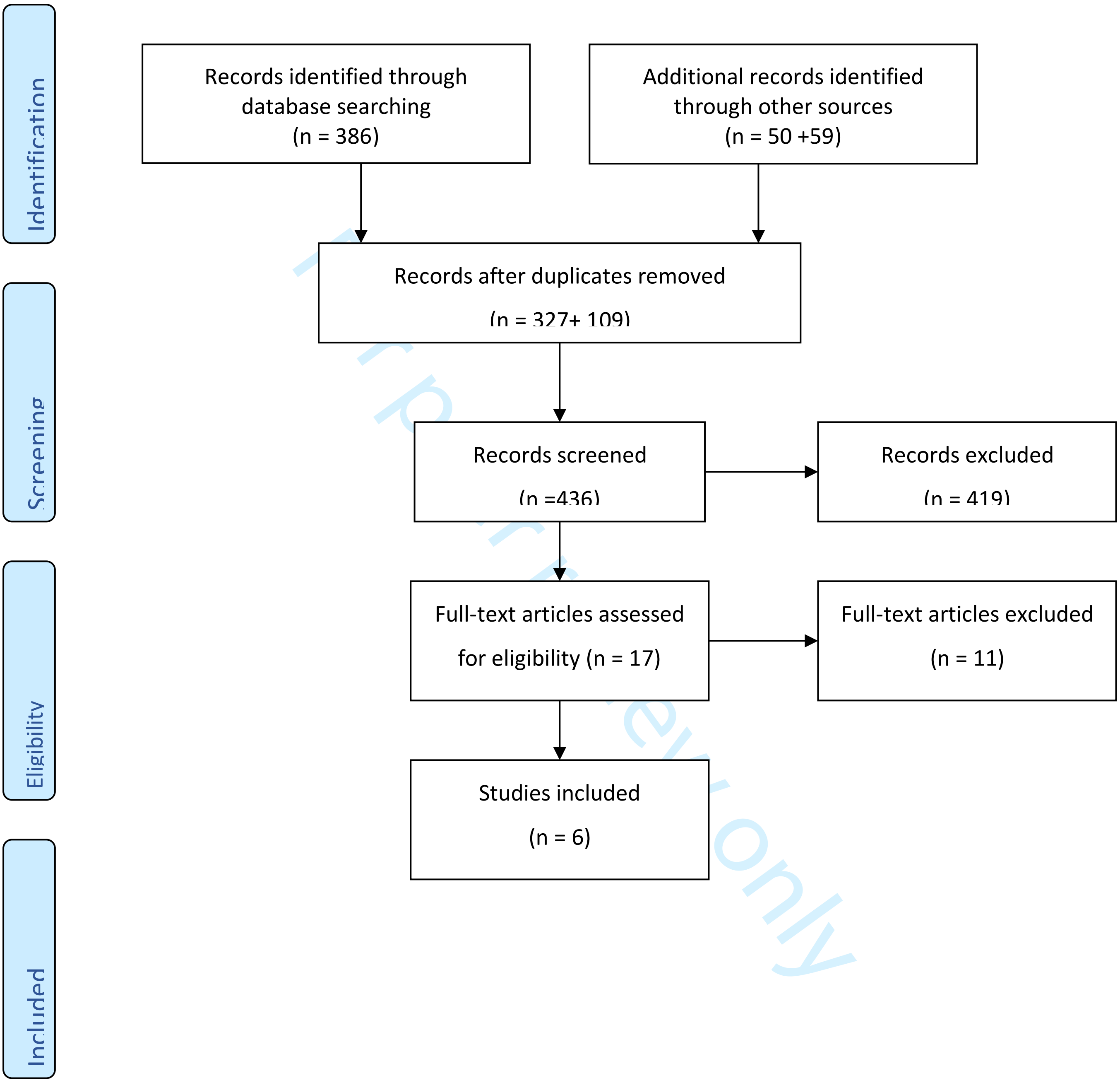

Figure F

PRISMA diagram: medicine reconciliation - outcomes

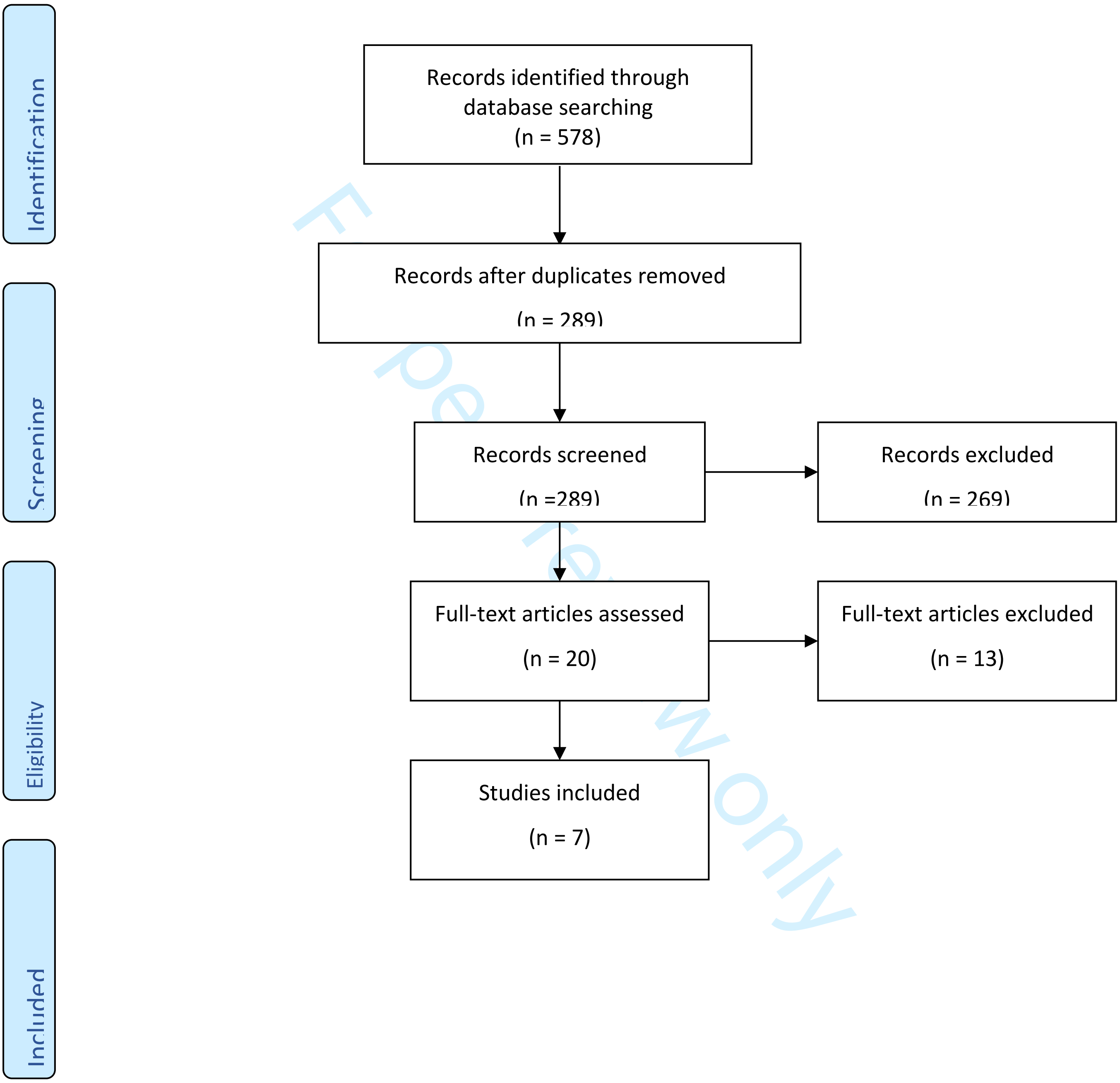

Theory Development Search Strategies

Structured subject searches

Policies, opinion pieces and research reports - Patient safety in networked IT systems search strategies

Search date: 10 /03/2018
Embase (Ovid) 1996 to 2018 Week 10

1 "Comment on".ti. (16738)
2 (letter* adj3 editor*).ti. (19988)
3 opinion*.ti. (12189)
4 (view or views).ti. (37100)
5 (editorial or journal letter or journal note or letter or note or report editorial or report letter or trade journal letter or trade journal note).pt. (1772500)
6 or/1-5 [opinion pieces - hidden theories] (1817733)
7 (policy or policies or guideline* or recommendation* or position).ti. (171688)
8 *practice guideline/ (54860)
9 exp *public policy/ or *policy/ (58445)
10 (theor* or concep* or logic).ti. (110842)
11 ((theor* or concep* or logic) adj (framework* or model* or analy* or evaluat*)).ab. (52231)
12 or/7-11 [Policy, Guideline or overt Theory] (383366)
13 6 or 12 [Theories search] (2155076)
14 *safety/ and (outpatient* or patient*).tw. (31576)
15 exp medical error/ and (outpatient* or patient*).tw. (51726)
16 ((patient or patients or outpatient*) adj2 (safety or harm* or adverse*)).tw. (54275)
17 exp *patient safety/ (12682)
18 *risk management/ (13493)
19 exp *adverse event/ (53911)
20 or/14-19 [patient safety] (196559)
21 ((health information or medical information or clinical information) adj5 (exchang* or shar* or network*)).tw,kw. (2649)
22 (patient information adj5 (exchang* or shar* or network*)).tw,kw. (285)
23 *medical information system/ (6904)
24 21 or 22 or 23 (9433)
25 exp medical record/ (176429)
26 information system/ or exp decision support system/ or exp hospital information system/ or medical information system/ or nursing information system/ or exp computerized provider order entry/ (77872)
27 medical informatics/ or nursing informatics/ (18665)
28 (electronic adj3 record*).tw. (46895)
29 ((health or medical or clinical) adj5 (information or record*)).tw. (300330)
30 computer system/ (20409)
31 or/25-30 [health records & systems] (462934)
32 exp computer network/ (11623)
33 information dissemination/ (17918)
34 (network* or exchange* or data shar*).tw. (603816)
35 (information adj3 (shar* or distribut* or disseminat*)).tw. (16717)
36 (record* adj3 (shar* or distribut* or disseminat*)).tw. (2683)
37 or/32-36 [networked systems] (641855)

For peer review only

38 31 and 37 (30994)
39 38 or 24 [Networked IT or HIEs] (36767)
40 20 and 39 [safety in networked systems] (1353)
41 13 and 40 [Theory & Opinion networked IT safety systems] (94)

Health Management Information Consortium (Ovid) 1983 - present
1 Commentaries/ (18)
2 "Comment on".ti. (29)
3 editorial.ti. (265)
4 letter.ti. (328)
5 opinion*.ti. (508)
6 (view or views).ti. (3538)
7 (letter* adj3 editor*).ti. (1)
8 or/1-7 (4667)
9 (evidence* adj8 (policy or policies)).tw. (1368)
10 policy/ or health policy/ or public policy/ (8823)
11 (policy or policies or guideline* or recommendation* or position).ti. (12107)
12 exp guidelines/ (6653)
13 (theor* or concep* or logic).ti. (2774)
14 ((theor* or concep* or logic) adj (framework* or model* or analy* or evaluat*)).ab. (1825)
15 or/9-14 [Policy, Guideline or overt Theory] (27515)
16 8 or 15 [Theories] (31886)
17 patient safety/ (3678)
18 adverse events/ (718)
19 exp Errors/ or exp Medication errors/ (1165)
20 ((patient or patients or outpatient*) adj2 (safety or harm* or adverse*)).tw. (2830)
21 risk management/ or safety measures/ (2818)
22 or/17-21 [patient safety] (7784)
23 information exchange/ (345)
24 exp Medical information exchange/ (20)
25 ((health information or medical information or clinical information) adj5 (exchang* or shar* or network*)).tw. (105)
26 (patient information adj5 (exchang* or shar* or network*)).tw. (38)
27 or/23-26 (485)
28 exp medical records/ (3144)
29 information technology/ (4796)
30 exp information systems/ (4895)
31 exp informatics/ (308)
32 (electronic adj3 record*).tw. (1319)
33 ((health or medical or clinical) adj5 (information or record*)).tw. (9925)
34 exp computer systems/ (4522)
35 or/28-34 [health records] (21052)
36 exp information transfer/ (1978)
37 exp computer networks/ (1667)
38 (network* or exchange* or data shar*).tw. (7125)
39 (information adj3 (shar* or distribut* or disseminat*)).tw. (1342)
40 (record* adj3 (shar* or distribut* or disseminat*)).tw. (145)
41 or/36-40 [networked] (11264)

For peer review only

42 35 and 41 (3305)
43 42 or 27 [networked IT , HIE] (3541)
44 22 and 43 [safety in networked systems] (67)
45 16 and 44 [Theory & Opinion networked IT safety systems] (3)

Ovid MEDLINE(R) 1946 to February Week 5 2018

1 (policy or policies or guideline* or recommendation* or position).ti. (157471)
2 guideline/ or practice guideline/ (29721)
3 policy/ or public policy/ or exp health policy/ (126430)
4 (theor* or concep* or logic).ti. (140361)
5 ((theor* or concep* or logic) adj (framework* or model* or analy* or evaluat*)).ab. (44188)
6 or/1-5 [Policy, Guideline or overt Theory] (445038)
7 Comment/ (664894)
8 Letter/ (926791)
9 Editorial/ (405400)
10 news/ or newspaper article/ (190273)
11 "Comment on".ti. (16286)
12 (letter* adj3 editor*).ti. (6376)
13 opinion*.ti. (11812)
14 (view or views).ti. (43579)
15 or/7-14 [Discussion papers Hidden Theory] (1710573)
16 6 or 15 [Theory Search] (2103940)
17 "Safety Management"/ and (outpatient* or patient*).tw. (7050)
18 "Medical Errors"/ and (outpatient* or patient*).tw. (7399)
19 patient safety/ (13787)
20 ((patient or patients or outpatient*) adj2 (safety or harm* or adverse*)).tw. (31734)
21 *risk management/ (8572)
22 or/17-21 [Patient Safety] (53654)
23 Health Information Exchange/ (562)
24 ((health information or medical information or clinical information) adj5 (exchang* or shar* or network*)).tw,kw. (2012)
25 (patient information adj5 (exchang* or shar* or network*)).tw,kw. (198)
26 23 or 24 or 25 (2551)
27 exp Medical Records/ (132774)
28 information systems/ or decision support systems, clinical/ or health information systems/ or exp management information systems/ (67432)
29 medical informatics/ or medical informatics applications/ or medical informatics computing/ or nursing informatics/ or public health informatics/ (15531)
30 exp Decision Making, Computer-Assisted/ (124934)
31 (electronic adj3 record*).tw. (20676)
32 ((health or medical or clinical) adj5 (information or record*)).tw. (190180)
33 Computer Systems/ (12550)
34 or/27-33 [health records] (484060)
35 community network/ (6385)
36 computer communication networks/ or local area networks/ (13961)
37 information dissemination/ (14049)
38 (network* or exchange* or data shar*).tw. (492792)
39 (information adj3 (shar* or distribut* or disseminat*)).tw. (12562)

For peer review only

40 (record* adj3 (shar* or distribut* or disseminat*)).tw. (2196)
41 or/35-40 [networking systems] (529805)
42 41 and 34 (28255)
43 42 or 26 [Networked IT systems] (28641)
44 43 and 22 [safety in networked systems] (468)
45 16 and 44 [Theory & Opinion networked IT safety systems] (47)

Ovid MEDLINE(R) Epub Ahead of Print March 08, 2018
(same strategy as Ovid MEDLINE(R) 1946 to February Week 5 2018)
Ovid MEDLINE(R) In-Process & Other Non-Indexed Citations March 08, 2018
(same strategy as Ovid MEDLINE(R) 1946 to February Week 5 2018)

Policies, opinion pieces and research reports – Interoperability and networked IT systems search strategies

Search date: 16-03-18

Embase Classic+Embase (Ovid) 1947 to 2018 March 15
1 "Comment on".ti. (20221)
2 (letter* adj3 editor*).ti. (24062)
3 opinion*.ti. (16905)
4 (view or views).ti. (61981)
5 (editorial or journal letter or journal note or letter or note or report editorial or report letter or trade journal letter or trade journal note).pt. (2268207)
6 or/1-5 [opinion pieces - hidden theories] (2344004)
7 (policy or policies or guideline* or recommendation* or position).ti. (222165)
8 *practice guideline/ (56643)
9 exp *public policy/ or *policy/ (85626)
10 (theor* or concep* or logic).ti. (182117)
11 ((theor* or concep* or logic) adj (framework* or model* or analy* or evaluat*)).ab. (60995)
12 or/7-11 [Policy, Guideline or overt Theory] (533584)
13 6 or 12 [Theories search] (2821744)
14 exp medical record/ (204622)
15 information system/ or exp decision support system/ or exp hospital information system/ or medical information system/ or nursing information system/ or exp computerized provider order entry/ (94578)
16 medical informatics/ or nursing informatics/ (19599)
17 (electronic adj3 record*).tw. (47862)
18 ((health or medical or clinical) adj5 (information or record*)).tw. (333516)
19 computer system/ (24959)
20 or/14-19 [health records & systems] (534936)
21 "data interoperability"/ (30)
22 interoperab*.tw,kw. (3422)
23 21 or 22 (3434)
24 23 and 20 and 13 [Theory interoperable health systems] (218)

HMIC Health Management Information Consortium 1983 - present
1 Commentaries/ (18)
2 "Comment on".ti. (29)

For peer review only

3 editorial.ti. (265)
4 letter.ti. (328)
5 opinion*.ti. (508)
6 (view or views).ti. (3538)
7 (letter* adj3 editor*).ti. (1)
8 or/1-7 (4667)
9 (evidence* adj8 (policy or policies)).tw. (1368)
10 policy/ or health policy/ or public policy/ (8823)
11 (policy or policies or guideline* or recommendation* or position).ti. (12107)
12 exp guidelines/ (6653)
13 (theor* or concep* or logic).ti. (2774)
14 ((theor* or concep* or logic) adj (framework* or model* or analy* or evaluat*)).ab. (1825)
15 or/9-14 [Policy, Guideline or overt Theory] (27515)
16 8 or 15 [Theories] (31886)
17 exp medical records/ (3144)
18 information technology/ (4796)
19 exp information systems/ (4895)
20 exp informatics/ (308)
21 (electronic adj3 record*).tw. (1319)
22 ((health or medical or clinical) adj5 (information or record*)).tw. (9925)
23 exp computer systems/ (4522)
24 or/17-23 [health records] (21052)
25 interoperab*.tw. (87)
26 16 and 24 and 25 (4)

Ovid MEDLINE(R) 1946 to March Week 2 2018

1 (policy or policies or guideline* or recommendation* or position).ti. (157588)
2 guideline/ or practice guideline/ (29719)
3 policy/ or public policy/ or exp health policy/ (126529)
4 (theor* or concep* or logic).ti. (140426)
5 ((theor* or concep* or logic) adj (framework* or model* or analy* or evaluat*)).ab. (44221)
6 or/1-5 [Policy, Guideline or overt Theory] (445305)
7 Comment/ (665509)
8 Letter/ (927149)
9 Editorial/ (405687)
10 news/ or newspaper article/ (190369)
11 "Comment on".ti. (16291)
12 (letter* adj3 editor*).ti. (6383)
13 opinion*.ti. (11818)
14 (view or views).ti. (43602)
15 or/7-14 [Discussion papers Hidden Theory] (1711671)
16 6 or 15 [Theory Search] (2105265)
17 exp Medical Records/ (132810)
18 information systems/ or decision support systems, clinical/ or health information systems/ or exp management information systems/ (67435)

For peer review only

19 medical informatics/ or medical informatics applications/ or medical informatics computing/ or nursing informatics/ or public health informatics/ (15539)
20 exp Decision Making, Computer-Assisted/ (125028)
21 (electronic adj3 record*).tw. (20720)
22 ((health or medical or clinical) adj5 (information or record*)).tw. (190394)
23 Computer Systems/ (12552)
24 or/17-23 [health records] (484384)
25 HEALTH INFORMATION INTEROPERABILITY/ (36)
26 interoperab*.tw,kw. (2537)
27 25 or 26 (2555)
28 16 and 24 and 27 [Theory interoperability records] (163)

Ovid MEDLINE(R) In-Process & Other Non-Indexed Citations March 15, 2018
(same search strategy as Ovid MEDLINE(R) 1946 to March Week 2 2018)

Ovid MEDLINE(R) Epub Ahead of Print March 15, 2018
(same search strategy as Ovid MEDLINE(R) 1946 to March Week 2 2018)

Related Articles

Named author searches

David Bates and Interoperable systems or Health Information Exchange

Search date: 22/03/2018

Embase Classic+Embase (Ovid) 1947 to 2018 March 21
1 exp medical record/ (205201)
2 information system/ or exp decision support system/ or exp hospital information system/ or medical information system/ or nursing information system/ or exp computerized provider order entry/ (94759)
3 medical informatics/ or nursing informatics/ (19627)
4 (electronic adj3 record*).tw. (48101)
5 ((health or medical or clinical) adj5 (information or record*)).tw. (334714)
6 computer system/ (24988)
7 or/1-6 [health records & systems] (536527)
8 "data interoperability"/ (31)
9 (interoperab* or inter-operab*).tw,kw. (3489)
10 8 or 9 (3502)
11 7 and 10 [interoperable systems] (2272)
12 *medical information system/ (7111)
13 ((health information or medical information or clinical information) adj5 (exchang* or shar* or network*)).tw,kw. (2874)
14 (patient information adj5 (exchang* or shar* or network*)).tw,kw. (301)
15 or/12-14 [health info exchange] (9873)
16 11 or 15 (11761)
17 Bates, DW.au. (865)
18 (Bates adj2 David).ti,ab,kw. (9)

For peer review only

19 or/17-18 (873)
20 19 and 16 [Bates on HIE and interoperability] (59)

HMIC Health Management Information Consortium (Ovid) 1983 - present
1 exp medical records/ (3144)
2 information technology/ (4796)
3 exp information systems/ (4895)
4 exp informatics/ (308)
5 (electronic adj3 record*).tw. (1319)
6 ((health or medical or clinical) adj5 (information or record*)).tw. (9925)
7 exp computer systems/ (4522)
8 or/1-7 [health records] (21052)
9 (interoperab* or inter-operab*).tw. (95)
10 8 and 9 [interoperable systems] (78)
11 information exchange/ (345)
12 exp Medical information exchange/ (20)
13 ((health information or medical information or clinical information) adj5 (exchang* or shar* or network*)).tw. (105)
14 (patient information adj5 (exchang* or shar* or network*)).tw. (38)
15 or/11-14 [health info exchange] (485)
16 10 or 15 (551)
17 Bates, D*.au. (17)
18 Bates.ti,ab. (12)
19 17 or 18 (29)
20 19 and 16 [Bates on HIE and interoperability] (1)

Ovid MEDLINE(R) 1946 to March Week 3 2018
1 exp Medical Records/ (132904)
2 information systems/ or decision support systems, clinical/ or health information systems/ or exp management information systems/ (67498)
3 medical informatics/ or medical informatics applications/ or medical informatics computing/ or nursing informatics/ or public health informatics/ (15554)
4 exp Decision Making, Computer-Assisted/ (125253)
5 (electronic adj3 record*).tw. (20785)
6 ((health or medical or clinical) adj5 (information or record*)).tw. (190733)
7 Computer Systems/ (12558)
8 or/1-7 [health records] (485075)
9 (interoperab* or inter-operab*).tw,kw. (2582)
10 9 and 8 [interoperable systems] (1800)
11 HEALTH INFORMATION INTEROPERABILITY/ (36)
12 10 or 11 (1820)
13 Health Information Exchange/ (567)
14 ((health information or medical information or clinical information) adj5 (exchang* or shar* or network*)).tw,kw. (2018)
15 (patient information adj5 (exchang* or shar* or network*)).tw,kw. (199)
16 or/13-15 [health information exchange] (2561)
17 12 or 16 (4170)
18 Bates, DW.au. (696)
19 (Bates adj2 David).ti,ab,kw. (7)
20 or/18-19 (702)

For peer review only

21 20 and 17 [Bates on HIE and interoperability] (29)

Ovid MEDLINE(R) In-Process & Other Non-Indexed Citations March 21, 2018
(Same search strategy as Ovid MEDLINE(R) 1946 to March Week 3 2018)

Ovid MEDLINE(R) Epub Ahead of Print March 21, 2018
(Same search strategy as Ovid MEDLINE(R) 1946 to March Week 3 2018)

Web of Science Core Collection (Clarivate Analytics): Arts & Humanities Citation Index 1975-present, Book Citation Index– Social Sciences & Humanities 2005-present, Conference Proceedings Citation Index- Science 1990-present, Conference Proceedings Citation Index- Social Science & Humanities 1990-present, Sciences Citation Index 1900-present , Social Sciences Citation Index 1900-present, Emerging Sources Citation Index 2015-present

14 28 #13 AND #3 3 [1 Bates and HIE/interoperability]**
13 3,837 #12 OR #8 2 [HIE/interop]
12 2,442 #11 OR #10 OR #9
11 208 TOPIC: (("patient information" NEAR/5 (exchang* or shar* or network*)))
10 2,265 TOPIC: ((("health information" or "medical information" or "clinical information") NEAR/5 (exchang* or shar* or network*)))
9 694 TOPIC: ("health information exchange")
8 1,618 #7 AND #6
7 25,500 TS=(interoperab* or inter-operab*)
6 212,986 #5 OR #4
5 207,575 TOPIC: (((health or medical or clinical) NEAR/5 (information or record*)))
4 28,520 TOPIC: ((electronic NEAR/3 record*))
3 921 #2 OR #1 [Bates as author]**
2 27 TOPIC: (Bates NEAR/2 David)
1 894 AUTHOR: (Bates DW)

**Bates is a prolific author on HIT technologies and patient safety so we only looked at i) articles he authored on HIT (line #14) or ii) any article he authored or where he was mentioned with a usage count ≥20 (line#3) which gave 74 references from the 921 identified
Robert Wachter

Search date: 10/03/2018
Embase (Ovid) 1996 to 2018 Week 10
1 wachter rm.au. (244)
2 wachter.ti,ab,kw. (40)
3 1 or 2 (284)
4 limit 3 to yr="2015 -Current" (32)

HMIC Health Management Information Consortium (Ovid) 1983 - present
1 wachter r*.au. (12)
2 wachter.ti,ab. (3)
3 1 or 2 (14)
4 limit 3 to yr="2015 -Current" (3)

For peer review only - http://bmjopen.bmj.com/site/about/guidelines.xhtml

Ovid MEDLINE(R) 1946 to February Week 5 2018

1 wachter rm.au. (232)
2 wachter.ti,ab,kw. (45)
3 1 or 2 (277)
4 limit 3 to yr="2015 -Current" (18)

Ovid MEDLINE(R) Epub Ahead of Print March 08, 2018
(same strategy as Ovid MEDLINE(R) 1946 to February Week 5 2018)
Ovid MEDLINE(R) In-Process & Other Non-Indexed Citations March 08, 2018
(same strategy as Ovid MEDLINE(R) 1946 to February Week 5 2018)

Web of Science Core Collection (Clarivate Analytics): Arts & Humanities Citation Index 1975-present, Book Citation Index– Social Sciences & Humanities 2005-present, Conference Proceedings Citation Index- Science 1990-present, Conference Proceedings Citation Index- Social Science & Humanities 1990-present, Sciences Citation Index 1900-present , Social Sciences Citation Index 1900-present, Emerging Sources Citation Index 2015-present
Search date: 21/03/18

PUBLICATION YEARS: (2015 OR 2016 OR 2017) *Note there was no option for 2018
3 41 #1 OR #2
2 27 AUTHOR: (wachter rm)
1 14 TOPIC: (wachter)

AHRQ PSNeT Patient Safety Network (AHRQ)
Search date: 28-2-2018
Search term 'Robert Wachter' was entered in the search tab. This retrieved all publications that were authored, or co-authored by Robert Wachter.

Usage count search

Search date: 22/03/2018
Web of Science Core Collection (Clarivate Analytics): Arts & Humanities Citation Index 1975-present, Book Citation Index– Social Sciences & Humanities 2005-present, Conference Proceedings Citation Index- Science 1990-present, Conference Proceedings Citation Index- Social Science & Humanities 1990-present, Sciences Citation Index 1900-present , Social Sciences Citation Index 1900-present, Emerging Sources Citation Index 2015-present

10 3,837 #9 OR #5 [HIE/interop]**
9 2,442 #8 OR #7 OR #6
8 208 TOPIC: (("patient information" NEAR/5 (exchang* or shar* or network*)))
7 2,265 TOPIC: ((("health information" or "medical information" or "clinical information") NEAR/5 (exchang* or shar* or network*)))
6 694 TOPIC: ("health information exchange")
5 1,618 #4 AND #3
4 25,500 TS=(interoperab* or inter-operab*)

For peer review only

3 212,986 #1 OR #2
2 207,575 TOPIC: (((health or medical or clinical) NEAR/5 (information or record*)))
1 28,520 TOPIC: ((electronic NEAR/3 record*))

**we only selected records with a usage count ≥50 accesses which gave 56 records from the 3837 identified

Project name search

Search date: 16/03/2018

Embase Classic+Embase (Ovid) 1947 to 2018 March 15

1 "connectingcare".ti,ab,kw.

2 "Connecting Care".ti,ab,kw.

3 1 or 2

4 "united king*".in,ad,ti.

5 (uk or "U.K." or "UK." or "U.K" or britain or scotland or ireland).in,ad,ti.

6 (england not "new england").in,ad,ti.

7 (wales not "new south wales").in,ad,ti.

8 exp United Kingdom/

9 (london or manchester or birmingham or leeds or sheffield or liverpool or newcastle or edinburgh or glasgow or cardiff or bristol).in,ad,ti.

10 ((london adj2 ontario) or (london adj on) or new london).in,ad,ti.

11 (manchester adj3 (USA or massach*)).in,ad,ti.

12 (newcastle adj4 (australia or "new south wales" or nsw)).in,ad,ti.

13 (liverpool adj4 (australia or "new south wales" or nsw)).in,ad,ti.

14 or/10-13

15 9 not 14

16 (british or english or scottish or welsh or irish).in,ti.

17 (nhs or "national health service").in,ti.

18 or/4-8,15-17

19 3 and 18

For peer review only

Systematic reviews searches

Search dates: 05/02/2018 and 05/07/2019
Cochrane Database of Systematic Reviews (Wiley) : Issue 7 of 12, July 2019

#1 MeSH descriptor: [Safety Management] explode all trees 164

#2 MeSH descriptor: [Medical Errors] explode all trees 2865

#3 #1 or #2 3008

#4 (outpatient* or patient*):ti,ab 864355

#5 #3 and #4 1959

#6 ((patient or patients or outpatient*) N2(safety or harm* or adverse)):ti,ab,kw 343

#7 MeSH descriptor: [Risk Management] explode all trees 8736

#8 MeSH descriptor: [Patient Safety] explode all trees 515

#9 #5 or #6 or #7 or #8 11261

#10 MeSH descriptor: [Medical Informatics] this term only 71

#11 MeSH descriptor: [Medical Informatics Applications] this term only 23

#12 MeSH descriptor: [Health Information Exchange] explode all trees 5

#13 MeSH descriptor: [Medical Informatics Computing] explode all trees 0

#14 MeSH descriptor: [Nursing Informatics] explode all trees 10

#15 MeSH descriptor: [Public Health Informatics] explode all trees 1

#16 MeSH descriptor: [Information Systems] explode all trees 2180

#17 MeSH descriptor: [Decision Making, Computer-Assisted] explode all trees 5060

#18 ("Information technolog*" or computer*):ti 4965

#19 MeSH descriptor: [Technology] this term only 29

#20 MeSH descriptor: [Biomedical Technology] explode all trees 21

#21 MeSH descriptor: [Computer Systems] explode all trees 5149

#22 #10 or #11 or #12 or #13 or #14 or #15 or #16 or #17 or #18 or #19 or #20 or #21 15121

#23 #9 and #22 640

DARE (Wiley): Issue 2 of 4, April 2015 (only run on 05/02/2018)
(Same search strategy as Cochrane Database of Systematic Reviews (Wiley) : Issue 7 of 12, July 2019)

Embase Classic+Embase (Ovid) 1947 to 2019 July 03

1 *safety/ and (outpatient* or patient*).tw. (32312)

2 medical error/ and (outpatient* or patient*).tw. (8475)

3 ((patient or patients or outpatient*) adj2 (safety or harm* or adverse*)).tw. (66683)

4 exp patient safety/ (112296)

5 *risk management/ (15976)

6 exp adverse event/ (591775)

7 or/1-6 (774080)

8 exp *information system/ (65112)

9 medical information system/ (20086)

10 medical informatics/ (19591)

11 ("Information technolog*" or computer*).ti. (90275)

12 *information technology/ or *medical technology/ or *technology/ (33552)

13 *computer system/ (6527)

14 or/8-13 [IT systems] (211487)

15 7 and 14 (5785)

16 limit 15 to "reviews (maximizes specificity)" (117)

Epistemonikos (Epistmonikos Foundation)

Two searches were conducted using the advanced search interface

1) *Search title*: information technology OR *search title/abstract* health information exchange AND *Search title/abstract*: patient safety OR Medical error* OR Adverse event* (26 results)

2) *Search title*: information technology OR information exchange OR interoperability AND
   Search title/abstract: patient safety OR Medical error* OR Adverse event* (44 results)

HTA Database (Wiley): Issue 4 of 4, October 2016 (only run on 05/02/2018)

(Same search strategy as Cochrane Database of Systematic Reviews (Wiley): Issue 2 of 12, February 2018)

Health Systems Evidence (McMaster University)

The search terms "health information exchange" AND “patient safety” were entered into the basic search interface (n=12)

For peer review only

Ovid MEDLINE(R) and Epub Ahead of Print, In-Process & Other Non-Indexed Citations and Daily 1946 to July 03, 2019 (includes MEDLINE 1946 to June Week 5 2019)

1 "Safety Management"/ and (outpatient* or patient*).tw. (7369)
2 "Medical Errors"/ and (outpatient* or patient*).tw. (7856)
3 patient safety/ (17099)
4 ((patient or patients or outpatient*) adj2 (safety or harm* or adverse*)).tw. (42840)
5 *risk management/ (8922)
6 or/1-5 [Patient Safety] (67271)
7 medical informatics/ (11141)
8 health information exchange/ (717)
9 medical informatics applications/ (2417)
10 medical informatics computing/ or nursing informatics/ or public health informatics/ (3347)
11 exp *information systems/ (82805)
12 exp *decision making, computer-assisted/ (96082)
13 ("Information technolog*" or computer*).ti. (74167)
14 *technology/ or *biomedical technology/ (8748)
15 exp *Computer Systems/ (89894)
16 or/7-15 [IT systems] (319910)
17 6 and 16 (3123)
18 limit 17 to "reviews (maximizes specificity)" (82)

For peer review only

Care Co-ordination Searches

What are the problems with the co-ordination of services for frail older people?

Search date: 06/08/2019
Structured database searches:
Applied Social Sciences Index and Abstracts (ASSIA) (ProQuest) 1987- present

Reviews search
((su(frail) OR (ti((function* NEAR/1 impair*) AND (old* OR elderly OR geriatric*)) OR ab((function* NEAR/1 impair*) AND (old* OR elderly OR geriatric*))) OR (ti((cognitive NEAR/1 impair*) AND (old* OR elderly OR geriatric*)) OR ab((cognitive NEAR/1 impair*) AND (old* OR elderly OR geriatric*))) OR (ti((complex NEAR/2 needs) AND (old* OR elderly OR geriatric*)) OR ab((complex NEAR/2 needs) AND (old* OR elderly OR geriatric*))) OR (ti(frail*) OR ab(frail*)))
AND
(su(Integrated care pathways) OR (ti((co-ordinat* OR coordinat* OR integrat* OR continuity) NEAR/3 (care OR service*)) OR ab((co-ordinat* OR coordinat* OR integrat* OR continuity) NEAR/3 (care OR service*))) OR su(continuity) OR su(Joint working) OR (ti((joint NEAR/1 (working OR care OR service*))) OR ab((joint NEAR/1 (working OR care OR service*)))) OR su(transition program) OR (ti((intermediate OR transition* OR transfer* OR team*) NEAR/3 care) OR ab((intermediate OR transition* OR transfer* OR team*) NEAR/3 care)) OR su(intercessional approach) OR su(multidisciplinary care) OR su(Interdisciplinary team work) OR su(Interdisciplinary approach) OR (ti((interinstitution* OR interagenc* OR interdisciplin* OR Interprofession* OR multiprofessional* OR multidisciplin* OR multiagenc*) NEAR/6 (relations* OR collaborat* OR co-ordinat* OR coordinat* OR communicat* OR care OR service*)) OR ab((interinstitution* OR interagenc* OR interdisciplin* OR interprofession* OR multiprofessional* OR multidisciplin* OR multiagenc*) NEAR/6 (relations* OR collaborat* OR co-ordinat* OR coordinat* OR communicat* OR care OR service*)))))
AND
(su(systematic review) OR ti(review OR meta-analysis OR synthesis))
n=10

Qualitative search
((MAINSUBJECT.EXACT.EXPLODE("Surveys") OR MAINSUBJECT.EXACT.EXPLODE("Interviews") OR MAINSUBJECT.EXACT.EXPLODE("Questionnaires") ti(((purpos* NEAR/4 sampl*) OR (focus NEAR/4 group*)) .) OR ab(((purpos* NEAR/4 sampl*) OR (focus NEAR/4 group*)) .)) OR (ti((grounded NEAR/4 (theor OR study OR studies OR research OR analys?s))) OR ab((grounded NEAR/4 (theor OR study OR studies OR research OR analys?s)))) OR (ti(phenomenol*.) OR ab(phenomenol*.)) OR (ti(qualitative) OR ab(qualitative)) OR (ti(ethnolog* OR ethnograph* OR ethnonursing) OR ab(ethnolog* OR ethnograph* OR ethnonursing)) OR su(Observational research) OR su(qualitative methods) OR su(qualitative research))
AND
((su(frail) OR (ti((function* NEAR/1 impair*) AND (old* OR elderly OR geriatric*)) OR ab((function* NEAR/1 impair*) AND (old* OR elderly OR geriatric*))) OR (ti((cognitive NEAR/1 impair*) AND (old* OR elderly OR geriatric*)) OR ab((cognitive NEAR/1 impair*) AND (old* OR elderly OR geriatric*))) OR (ti((complex NEAR/2 needs) AND

For peer review only

(old* OR elderly OR geriatric*)) OR ab((complex NEAR/2 needs) AND (old* OR elderly OR geriatric*))) OR (ti(frail*) OR ab(frail*)))
AND
(su(Integrated care pathways) OR (ti((co-ordinat* OR coordinat* OR integrat* OR continuity) NEAR/3 (care OR service*)) OR ab((co-ordinat* OR coordinat* OR integrat* OR continuity) NEAR/3 (care OR service*))) OR su(continuity) OR su(Joint working) OR (ti((joint NEAR/1 (working OR care OR service*))) OR ab((joint NEAR/1 (working OR care OR service*)))) OR su(transition program) OR (ti((intermediate OR transition* OR transfer* OR team*) NEAR/3 care) OR ab((intermediate OR transition* OR transfer* OR team*) NEAR/3 care)) OR su(intercessional approach) OR su(multidisciplinary care) OR su(Interdisciplinary team work) OR su(Interdisciplinary approach) OR (ti((interinstitution* OR interagenc* OR interdisciplin* OR Interprofession* OR multiprofessional* OR multidisciplin* OR multiagenc*) NEAR/6 (relations* OR collaborat* OR co-ordinat* OR coordinat* OR communicat* OR care OR service*)) OR ab((interinstitution* OR interagenc* OR interdisciplin* OR interprofession* OR multiprofessional* OR multidisciplin* OR multiagenc*) NEAR/6 (relations* OR collaborat* OR co-ordinat* OR coordinat* OR communicat* OR care OR service*)))))

n=57

CINAHL (EBSCO) 1981- present
Note, records downloaded from S30 (reviews search) and S44 (qualitative studies search)
S44 S18 AND S42 193 Limiters - Published Date: 20000101-20181231
S43 S18 AND S42 202
S42 S40 OR S41 452,543
S41 ((MH "Interviews+") or (MH "Questionnaires+")) OR ((MH "Grounded Theory") or (MH "Research, Nursing")) OR ((MH "Ethnographic Research") or (MH "Ethnonursing Research") or (MH "Ethnological Research")) 376,306
S40 S21 OR S22 OR S23 OR S24 OR S25 OR S26 OR S27 OR S28 OR S29 OR S30 OR S31 OR S32 OR S33 OR S34 OR S35 OR S36 OR S37 OR S38 OR S39 184,864
S39 TX narrative analys?s 1,981
S38 TX ((discourse* or discurs*) N3 analys?s 3,857
S37 TX content analys?s 29,365
S36 TX observational method* 16,252
S35 TX cluster sampl* 3,625
S34 TX ((purpos* N4 sampl*) or (focus N1 group*)) 53,728
S33 TX (action research or cooperative inquir* or co operative inquir* or co-operative inquir*) 7,632
S32 TX participant observ* 8,986
S31 TX (emic or etic or hermeneutic* or heuristic* or semiotic*) 4,619
S30 TX data N1 saturat* 447
S29 TX grounded N1 (theor* or stud* or research or analys?s) 13,658
S28 TX Ethnonursing OR TX ethnograph* or TX ethnolog* 31,218
S27 (MH "Phenomenology") 2,529
S26 (MH "Field Studies")2,264
S25 (MH "Observational Methods+") 16,978
S24 (MH "Content Analysis") 24,944

S23 (MH "Discourse Analysis") 3,303
S22 (MH "Focus Groups") 28,512
S21 (MH "Qualitative Studies+") 96,870
S20 S18 AND S19 30
S19 Limiters - Published Date: 20000101-20181231; Publication Type: Systematic Review 55,215
S18 S6 AND S17 784
S17 S7 OR S8 OR S9 OR S10 OR S11 OR S12 OR S13 OR S14 OR S15 OR
S16 72,963
S16 TI (((interinstitution* or interagenc* or interdisciplin* or interprofession* or multiprofessional* or multidisciplin* or multiagenc*) N6 (relations* or collaborat* or co-ordinat* or coordinat* or communicat* or care or service*)) 3,115
S15 TI ((intermediate or transition* or transfer* or team*) N3 care) 4,346
S14 (MH "Transitional Care") 530
S13 (MH "Interprofessional Relations+") 20,004
S12 (MH "Transfer, Discharge")3,884
S11 TI (joint N1 (working or care or service*)) OR AB (joint N1 (working or care or service*)) 524
S10 (MH "Continuity of Patient Care+") 12,923
S9 (MH "Health Care Delivery, Integrated") 6,466
S8 TI ((co-ordinat* or coordinat* or integrat* or continuity) N3 (care or service*)) 5,770
S7 (MH "Multidisciplinary Care Team+") 28,652
S6 S1 OR S2 OR S3 OR S4 OR S5 15,529
S5 TI frail* OR AB frail* 6,412
S4 TI ((complex* N2 needs) AND (old* or elderly or geriatric*)) OR AB ((complex* N2 needs) AND (old* or elderly or geriatric*)) 353
S3 TI ((cognitive* N6 impair*) AND (old* or elderly or geriatric*)) OR AB ((cognitive* N6 impair*) AND (old* or elderly or geriatric*)) 5,464
S2 TI ((function* N6 impair*) AND (old* or elderly or geriatric*)) OR AB ((function* N6 impair*) AND (old* or elderly or geriatric*)) 2,556
S1 (MH "Frail Elderly") OR (MH "Frailty Syndrome") 5,380

HMIC Health Management Information Consortium (Ovid) 1983 - present
Note, records downloaded from line 46 (reviews search) and line 41 (qualitative studies search)
1 exp frail older people/ or exp Frail elderly people/ (314)
2 ((function* adj1 impair*) and (old* or elderly or geriatric*)).tw,hw. (48)
3 ((cognitive* adj1 impair*) and (old* or elderly or geriatric*)).tw,hw. (265)
4 ((complex* adj2 needs) and (old* or elderly or geriatric*)).tw,hw. (129)
5 frail*.tw. (989)
6 or/1-5 [frailty] (1444)
7 collaborative care/ or integrated care/ or interagency collaboration/ or joint working/ (5576)
8 ((co-ordinat* or coordinat* or integrat* or continuity) adj3 (care or service*)).tw,hw. (6550)
9 (joint adj (working or care or service*)).tw,hw. (2667)
10 ((intermediate or transition* or transfer* or team*) adj3 care).tw,hw. (4138)
11 patient transfer/ (348)
12 health care teams/ (137)

13 long term care/ (1778)
14 exp Intermediate care/ (518)
15 exp interorganisational relations/ (5496)
16 ((interinstitution* or interagenc* or interdisciplin* or interprofession* or multiprofessional* or multidisciplin* or multiagenc*) adj6 (relations* or collaborat* or co-ordinat* or coordinat* or communicat* or care or service*)).tw. (1881)
17 or/7-16 [care coordination] (19596)
18 6 and 17 (294)
19 limit 18 to yr="2000 -Current" (223)
20 exp qualitative techniques/ (323)
21 mixed methods research/ or qualitative research/ (1250)
22 qualitative.tw. (8732)
23 (ethnolog* or ethnograph* or ethnonursing).tw. (691)
24 phenomenol*.af. (457)
25 (grounded adj (theor$ or study or studies or research or analys?s)).af. (687)
26 (action research or cooperative inquir* or co operative inquir* or co-operative inquir*).mp. (745)
27 (emic or etic or hermeneutic* or heuristic* or semiotic*).af. or (data adj1 saturat*).tw. or participant observ*.tw. (774)
28 ((purpos* adj4 sampl*) or (focus adj group*)).af. (3723)
29 (life world or life-world or conversation analys?s or personal experience* or theoretical saturation).mp. (506)
30 cluster sampl*.mp. (40)
31 observational method*.af. (36)
32 content analysis.af. (1021)
33 ((discourse* or discurs*) adj3 analys?s).tw. (146)
34 narrative analys?s.af. (48)
35 or/20-34 [adapted qualitasitve filter] (13942)
36 qualitative research/ (1244)
37 interviews/ (952)
38 interviews.tw. (11703)
39 or/35-38 [qual & interviews] (20362)
40 18 and 39 (46)
41 limit 40 to yr="2000 -Current" (43)
42 systematic reviews/ or literature reviews/ or meta analysis/ (6475)
43 ((systematic or metaanalys* or meta-analys* or rapid or evidence or qualitative or realist) adj2 (review or synthesis)).tw. (4442)
44 42 or 43 (8119)
45 18 and 44 (15)
46 limit 45 to yr="2000 -Current" (13)

Ovid MEDLINE(R) 1996 to July Week 4 2018
Note, records downloaded from line 22 (reviews search) and line 48 (qualitative studies search)

1 Frail Elderly/ or Frailty/ (9088)
2 "Aged, 80 and over"/ and (frail* or complex).tw. (24288)
3 ((function* adj1 impair*) and (old* or elderly or geriatric*)).tw. (3174)
4 ((cognitive* adj1 impair*) and (old* or elderly or geriatric*)).tw. (13985)
5 ((complex* adj2 needs) and (old* or elderly or geriatric*)).tw. (362)

For peer review only

6 frail*.tw,kw. (12564)
7 or/1-6 [frailty] (49873)
8 ((co-ordinat* or coordinat* or integrat* or continuity) adj3 (care or service*)).ti,kw. (5599)
9 "Delivery of Health Care, Integrated"/ (10714)
10 *"Continuity of Patient Care"/ (7808)
11 (joint adj (working or care or service*)).tw,kw. (418)
12 Patient Transfer/ (6188)
13 *Patient Care Team/ (18406)
14 ((intermediate or transition* or transfer* or team*) adj3 care).ti,kw. (3712)
15 Transitional Care/ (389)
16 Intermediate Care Facilities/ (365)
17 *interprofessional relations/ or *interdisciplinary communication/ (18983)
18 ((Interinstitution* or interagenc* or interdisciplin* or interprofession* or multiprofessional* or multidisciplin* or multiagenc*) adj6 (relations* or collaborat* or co-ordinat* or coordinat* or communicat* or care or service*)).ti,kw. (3305)
19 or/8-18 [Care co-ordination] (63400)
20 7 and 19 [Care co-ordination in the frail] (837)
21 limit 20 to (meta analysis or systematic reviews) [1a Frailty care co-ordination reviews] (50)
22 limit 21 to yr="2000 -Current" (50)
23 limit 20 to (yr="2000 -Current" and "qualitative (maximizes specificity)") (82)
24 exp qualitative research/ (40247)
25 qualitative.tw,kf. (133174)
26 Nursing Methodology Research/ (14985)
27 observational study/ (50230)
28 ethnolog*.mp. (124547)
29 ethnograph*.mp. (7008)
30 ethnonursing.af. (85)
31 phenomenol*.af. (14598)
32 (grounded adj (theor$ or study or studies or research or analys?s)).af. (8093)
33 (emic or etic or hermeneutic* or heuristic* or semiotic*).af. or (data adj1 saturat*).tw. or participant observ*.tw. (14268)
34 (action research or cooperative inquir* or co operative inquir* or co-operative inquir*).mp. (2724)
35 ((purpos* adj4 sampl*) or (focus adj group*)).af. (42519)
36 (life world or life-world or conversation analys?s or personal experience* or theoretical saturation).mp. (7793)
37 cluster sampl*.mp. (4726)
38 observational method*.af. (462)
39 content analysis.af. (16341)
40 ((discourse* or discurs*) adj3 analys?s).tw. (1529)
41 narrative analys?s.af. (810)
42 or/24-41 [qualitative] (383980)
43 "Surveys and Questionnaires"/ (361279)
44 INTERVIEW/ (17657)
45 interviews.tw,kf. (112381)
46 or/42-45 [qual & interviews] (759992)
47 20 and 46 (191)
48 limit 47 to yr="2000 -Current" (185)

For peer review only

Ovid MEDLINE(R) Epub Ahead of Print August 03, 2018
(Same search strategy as Ovid MEDLINE(R) 1996 to July Week 4 2018)

Ovid MEDLINE(R) In-Process & Other Non-Indexed Citations August 03, 2018
(Same search strategy as Ovid MEDLINE(R) 1996 to July Week 4 2018)

PsycINFO (Ovid) 1806 to July Week 5 2018
Note, records downloaded from line 17 (reviews search) and line 41 (qualitative studies search)

1 health impairments/ (2299)
2 *geriatric patients/ or elder care/ (13064)
3 ((function* adj1 impair*) and (old* or elderly or geriatric*)).tw. (1987)
4 ((cognitive* adj1 impair*) and (old* or elderly or geriatric*)).tw. (12949)
5 ((complex* adj2 needs) and (old* or elderly or geriatric*)).tw. (279)
6 frail*.tw. (4263)
7 cognitive impairment/ and (old* or elderly or geriatric*).tw. (9247)
8 or/1-7 [frailty] (33360)
9 ((co-ordinat* or coordinat* or integrat* or continuity) adj3 (care or service*)).ti,id. (4237)
10 integrated services/ (3213)
11 interdisciplinary treatment approach/ (6866)
12 "continuum of care"/ (1628)
13 (joint adj (working or care or service*)).tw,id. (238)
14 ((intermediate or transition* or transfer* or team*) adj3 care).ti,id. (1981)
15 ((interinstitution* or interagenc* or interdisciplin* or interprofession* or multiprofessional* or multidisciplin* or multiagenc*) adj6 (relations* or collaborat* or co-ordinat* or coordinat* or communicat* or care or service*)).ti,id. (3090)
16 or/9-15 (17309)
17 8 and 16 [care coordination in the frail elderly] (516)
18 limit 17 to ("reviews (maximizes specificity)" and yr="2000 -Current") (19)
19 exp qualitative research/ (7890)
20 qualitative.tw,id. (144618)
21 survey*.mp. (301041)
22 ethnolog*.mp. (2683)
23 ethnograph*.mp. (26448)
24 ethnonursing.af. (138)
25 phenomenol*.af. (104146)
26 (grounded adj (theor$ or study or studies or research or analys?s)).af. (50918)
27 (emic or etic or hermeneutic* or heuristic* or semiotic*).af. or (data adj1 saturat*).tw. or participant observ*.tw. (90872)
28 (action research or cooperative inquir* or co operative inquir* or co-operative inquir*).mp. (8286)
29 ((purpos* adj4 sampl*) or (focus adj group*)).af. (57965)
30 (life world or life-world or conversation analys?s or personal experience* or theoretical saturation).mp. (13865)
31 cluster sampl*.mp. (1382)
32 observational method*.af. (1952)
33 observation methods/ (5201)

For peer review only

34 or/19-33 [qualitative terms] (670126)
35 interviews/ (8107)
36 questionnaires/ (17252)
37 interview*.tw,id. (297486)
38 questionnaire*.mp. (369227)
39 or/34-38 [qual & interviews] (1076024)
40 17 and 39 (168)
41 limit 40 to yr="2000 -Current" (154)

Complementary Searches:

Kings Fund – hand searched
The King's Fund Integrated Care topic webpage, publications list (https://www.kingsfund.org.uk/topics/integrated-care?f%5B0%5D=type%3A5842)
The King's Fund Integrated care and partnership working reading list (https://koha.kingsfund.org.uk/cgi-bin/koha/opac-shelves.pl?op=view&shelfnumber=108&sortfield=copyrightdate&direction=desc)
n=137

The Journal of Integrated Care – hand searched
Titles in all issues from 2014-present were screened for relevance and possibly included studies were downloaded.
n=72

For peer review only

What are the experiences of users of the networked HIT in facilitating the coordination of care?

Search date: 30/09/2018
Structured database searches:
ACM Digital Library (full text) 1947 – present

ACM Guide to Computing Literature – Advanced Search Mode
1 [any field – matches all] interoperab* record* AND [any field – any] health clinical medical patient AND [any field all] review = 5
2 [any field – matches all] [interoperab* AND [any field – any] metaanalysis =0
3 [any field – matches all] interoperab* AND [any field – all] Coordinat* AND [any field all] review=0
4 [any field – matches all] interoperab* AND [any field – all] communicat* AND [any field all] review =2
5 [any field – matches all] interoperab* AND [any field – al] collaborat* AND [any field all] review =0
6 [any field – matches all] interoperab* AND [any field – all] integrat* AND [any field all] review =0
7 [any field – matches all] interoperab* AND [any field – all] transition* AND [any field all] review =0
8 [any field – matches all] “health information exchange” [any field all] AND care [any field] AND [any field all] review =6
9 [any field – matches all] “health information exchange” [any field all] AND record* [any field] AND [any field all] review =9
10 [any field – matches all] “health information exchange” [any field all] AND coordinat* [any field] AND [any field all] review =0
11 [any field – matches all] “health information exchange” [any field all] AND communicat* [any field] AND [any field all] review =0
12 [any field – matches all] “health information exchange” [any field all] AND integrat* [any field] AND [any field all] review =0
13 [any field – matches all] “health information exchange” [any field all] AND transition* [any field] AND [any field all] review =0
14 [any field – matches all] “health information exchange” [any field all] AND [any field all] review =14

Combined 14 searches (n=36)

ASSIA (ProQuest) 1987 – present

(((((MAINSUBJECT.EXACT.EXPLODE("Health records") OR
MAINSUBJECT.EXACT.EXPLODE("Medical records") OR
MAINSUBJECT.EXACT("Computerized medical records")) OR
MAINSUBJECT.EXACT("Records")) OR TI,AB(electronic NEAR/3 record*) OR
TI,AB((health or medical or clinical) NEAR/5 (information or record)))
AND
TI,AB(interoperab*)) OR TI,AB("health information exchange"))
AND

(TI,AB((co-ordinat* OR coordinat* OR integrat* OR continuity) NEAR/3 (care OR service*)) OR ((MAINSUBJECT.EXACT("Care") OR MAINSUBJECT.EXACT("Integrated care pathways") OR MAINSUBJECT.EXACT("Intermediate care") OR MAINSUBJECT.EXACT("Shared care") OR MAINSUBJECT.EXACT("Continuing care") OR MAINSUBJECT.EXACT("Home care")) OR (MAINSUBJECT.EXACT("Transition programmes") OR MAINSUBJECT.EXACT("Continuity")) OR (MAINSUBJECT.EXACT("Care coordinators") OR MAINSUBJECT.EXACT("Care management") OR MAINSUBJECT.EXACT("Care delivery")) OR MAINSUBJECT.EXACT("Joint working")) OR Ti,AB(joint NEAR/1 (working OR care OR service*)) OR TI,AB((intermediate OR transition* OR transfer* OR team*) NEAR/3 care) OR (MAINSUBJECT.EXACT.EXPLODE("Interdisciplinary team work") OR MAINSUBJECT.EXACT("Interdisciplinary approach")) OR TI,AB((interinstitution* OR interagenc* OR interdisciplin* OR Interprofession* OR multiprofessional* OR multidisciplin* OR multiagenc*) NEAR/6 (relations* OR collaborat* OR co-ordinat* OR coordinat* OR communicat* OR care OR service*)))
AND
at.exact("Literature Review") 1

Embase (Ovid) 1996 to 2018 Week 40
1 ((co-ordinat* or coordinat* or integrat* or continuity) adj3 (care or service*)).tw,kw. (42958)
2 integrated health care system/ (9709)
3 transitional care/ (1675)
4 *patient care/ (49167)
5 (joint adj (working or care or service*)).tw,kw. (790)
6 patient transport/ (19866)
7 ((intermediate or transition* or transfer* or team*) adj3 care).tw,kw. (35683)
8 Intermediate Care Facilities/ (34086)
9 interdisciplinary communication/ (10573)
10 ((interinstitution* or interagenc* or interdisciplin* or interprofession* or multiprofessional* or multidisciplin* or multiagenc*) adj6 (relations* or collaborat* or co-ordinat* or coordinat* or communicat* or care or service*)).tw. (33105)
11 interpersonal communication/ (124500)
12 public relations/ (37161)
13 or/1-12 [care-coordination] (345988)
14 exp medical record/ (186463)
15 information system/ or exp decision support system/ or exp hospital information system/ or medical information system/ or nursing information system/ or exp computerized provider order entry/ (81079)
16 medical informatics/ or nursing informatics/ (19364)
17 (electronic adj3 record*).tw. (52350)
18 ((health or medical or clinical) adj5 (information or record*)).tw. (321597)
19 computer system/ (20637)
20 or/14-19 [health records & systems] (489700)
21 data interoperability/ (77)
22 interoperab*.tw,kw. (3660)
23 21 or 22 (3687)
24 20 and 23 [interoperable records - based on theory search 3 interoperability] (2396)

25 *medical information system/ (7112)
26 24 or 25 (9298)
27 13 and 26 [care coordination interoperability] (969)
28 limit 27 to yr="2000 -Current" (956)
29 limit 28 to "reviews (best balance of sensitivity and specificity)" (141)

INSPEC (EI Village) 1896 to present

((($meta-analysis OR $review OR search*) WN ALL))
AND
((((((({health information exchange}) WN ALL))) OR ((((((((({electronic health records} WN CV) OR ({medical information systems} WN CV) OR ({information systems} WN CV))))) OR ((($electronic NEAR/3 record* OR $health NEAR/3 record* OR $medical NEAR/3 record* OR $clinical NEAR/3 record* OR $health NEAR/3 $information OR $medical NEAR/3 $information OR $clinical NEAR/3 $information) WN ALL))))
AND
((((((({open systems} WN CV)))))) OR (((interoperab*) WN ALL)))))))))
AND
((((((({patient care} WN CV) OR ({health care} WN CV) OR ({professional communication} WN CV)))))) OR ((continuity NEAR/3 care OR continuity NEAR/3 service OR integrat* NEAR/3 service OR integrat* NEAR/3 care OR Coordinat* NEAR/3 care OR Co-ordinat* NEAR/3 service)) OR ((({joint working} OR {joint care} OR {joint service}) WN ALL))) OR ((($intermediate NEAR/3 $Care OR transition* NEAR/3 $Care OR $transfer NEAR/3 $Care OR $team NEAR/3 $Care OR $shared NEAR/3 $Care) WN ALL)) OR (((($interinstitution OR $interagency OR interdisciplin* OR interprofession* OR multiprofession* OR multidisciplin* OR multiagenc*)
AND
(relations* OR collaborat* OR co-ordinat* OR coordinat* OR communicat* OR $care OR service*)) WN ALL) AND (1896-2019 WN YR)))))
AND
(2000-2019 WN YR))
132 results

Ovid MEDLINE(R) 1946 to September Week 3 2018

1 ((co-ordinat* or coordinat* or integrat* or continuity) adj3 (care or service*)).tw,kw. (28296)
2 "Delivery of Health Care, Integrated"/ (11146)
3 *"Continuity of Patient Care"/ (9463)
4 (joint adj (working or care or service*)).tw,kw. (551)
5 Patient Transfer/ (7453)
6 *Patient Care Team/ (25367)
7 ((intermediate or transition* or transfer* or team*) adj3 care).tw,kw. (20298)
8 Transitional Care/ (414)
9 Intermediate Care Facilities/ (675)
10 *interprofessional relations/ or *interdisciplinary communication/ (25620)

For peer review only

11 ((Interinstitution* or interagenc* or interdisciplin* or interprofession* or multiprofessional* or multidisciplin* or multiagenc*) adj6 (relations* or collaborat* or co-ordinat* or coordinat* or communicat* or care or service*)).tw,kw. (19897)
12 or/1-11 [care co-ordination] (121977)
13 exp Medical Records/ (135875)
14 information systems/ or decision support systems, clinical/ or health information systems/ or exp management information systems/ (68814)
15 medical informatics/ or medical informatics applications/ or medical informatics computing/ or nursing informatics/ or public health informatics/ (16030)
16 exp Decision Making, Computer-Assisted/ (129591)
17 (electronic adj3 record*).tw. (22494)
18 ((health or medical or clinical) adj5 (information or record*)).tw. (199314)
19 Computer Systems/ (12730)
20 or/13-19 [health records] (501264)
21 Health Information Interoperability/ (64)
22 interoperab*.tw,kw. (2744)
23 21 or 22 (2769)
24 23 and 20 [interoperable records - based on theory search 3 interoperability] (1927)
25 Health Information Exchange/ (638)
26 24 or 25 (2499)
27 12 and 26 [coordinated care and interoperable systems] (270)
28 limit 27 to yr="2000 -Current" (267)
29 limit 28 to "reviews (best balance of sensitivity and specificity)" (17)

Ovid MEDLINE(R) In-Process & Other Non-Indexed Citations September 28, 2018
(same strategy as Ovid MEDLINE(R) 1946 to September Week 3 2018)

Ovid MEDLINE(R) Epub Ahead of Print September 25, 2018
(same strategy as Ovid MEDLINE(R) 1946 to September Week 3 2018)

Sociological Abstracts (ProQuest) 1952 – present

(((TI,AB(electronic NEAR/3 record*) OR TI,AB((health or medical or clinical) NEAR/5 (information or record))) AND TI,AB(interoperab*)) OR TI,AB("health information exchange"))
AND
(TI,AB((co-ordinat* OR coordinat* OR integrat* OR continuity) NEAR/3 (care OR service*)) OR TI,AB(joint NEAR/1 (working OR care OR service*)) OR TI,AB((intermediate OR transition* OR transfer* OR team*) NEAR/3 care) OR (MAINSUBJECT.EXACT("Cooperation") OR MAINSUBJECT.EXACT("Teamwork") OR MAINSUBJECT.EXACT("Interdisciplinary Approach")) OR TI,AB((interinstitution* OR interagenc* OR interdisciplin* OR Interprofession* OR multiprofessional* OR multidisciplin* OR multiagenc*) NEAR/6 (relations* OR collaborat* OR co-ordinat* OR coordinat* OR communicat* OR care OR service*)))
Narrowed by: Document type: Review
n=0

Web of Science Core Collection: Citation Indexes (Clarivate Analytics) 1900 – present

Indexes=SCI-EXPANDED, SSCI, A&HCI, CPCI-S, CPCI-SSH, ESCI
Timespan=2000-2018
# 17 17 #16 OR #15
# 16 9 #14 AND #13
# 15 10 #12 AND #5 Refined by: DOCUMENT TYPES: (REVIEW)
# 14 758,107 TS=(meta-analysis or search*)
# 13 297 #12 AND #5
# 12 2,350 #11 OR #10
# 11 766 TOPIC: ("health information exchange")
# 10 1,700 #9 AND #8
# 9 24,924 TOPIC: ((interoperab* or inter-operab*))
# 8 198,344 #7 OR #6
# 7 193,461 TOPIC: ((((health or medical or clinical) NEAR/5 (information or record*))))
# 6 30,013 TOPIC: (((electronic NEAR/3 record*)))
# 5 87,790 #4 OR #3 OR #2 OR #1
# 4 25,278 TOPIC: ((Interinstitution* or interagenc* or interdisciplin* or interprofession* or multiprofessional* or multidisciplin* or multiagenc*) near/6 (relations* or collaborat* or co-ordinat* or coordinat* or communicat* or care or service*))
# 3 23,166 TOPIC: ((intermediate or transition* or transfer* or team*) near/3 care)
# 2 2,884 TOPIC: (joint near/1 (working or care or service))
# 1 44,446 TOPIC: ((co-ordinat* or coordinat* or integrat* or continuity) NEAR/3 (care or service*))

Complementary searches:

Forward citation searches of Fitzpatrick G, Ellingsen G. A review of 25 years of CSCW research in healthcare: contributions, challenges and future agendas. Computer Supported Cooperative Work (CSCW). 2013 Aug 1;22(4-6):609-65. In the following sources

- ACM Digital Library (full text) 1947 to present
- Google Scholar
- Scopus (Elsevier B.V.) 1823 – present
- Web of Science Core Collection: Citation Indexes (Clarivate Analytics) 1900 – present

For peer review only

Can the use of networked IT in the care co-ordination of older people improve patient safety?

Search date: 30/09/2018 and updated 03/03/2019
Structured database searches

Applied Social Sciences Index and Abstracts (ASSIA) (ProQuest) 1987- present

((((MAINSUBJECT.EXACT("Care") OR MAINSUBJECT.EXACT("Integrated care pathways") OR MAINSUBJECT.EXACT("Intermediate care") OR MAINSUBJECT.EXACT("Shared care") OR MAINSUBJECT.EXACT("Continuing care") OR MAINSUBJECT.EXACT("Home care")) OR (MAINSUBJECT.EXACT("Transition programmes") OR MAINSUBJECT.EXACT("Continuity")) OR (MAINSUBJECT.EXACT("Care coordinators") OR MAINSUBJECT.EXACT("Care management") OR MAINSUBJECT.EXACT("Care delivery")) OR MAINSUBJECT.EXACT("Joint working")) OR TI,AB(joint NEAR/1 (working OR care OR service*)) OR TI,AB((intermediate OR transition* OR transfer* OR team*) NEAR/3 care) OR (MAINSUBJECT.EXACT.EXPLODE("Interdisciplinary team work") OR MAINSUBJECT.EXACT("Interdisciplinary approach")) OR TI,AB((interinstitution* OR interagenc* OR interdisciplin* OR Interprofession* OR multiprofessional* OR multidisciplin* OR multiagenc*) NEAR/6 (relations* OR collaborat* OR co-ordinat* OR coordinat* OR communicat* OR care OR service*)))
AND
(((((MAINSUBJECT.EXACT.EXPLODE("Health records") OR MAINSUBJECT.EXACT.EXPLODE("Medical records") OR MAINSUBJECT.EXACT("Computerized medical records")) OR MAINSUBJECT.EXACT("Records")) OR TI,AB(electronic NEAR/3 record*) OR TI,AB((health or medical or clinical) NEAR/5 (information or record))) AND TI,AB(interoperab*)) OR TI,AB("information exchang*")))
AND
((MAINSUBJECT.EXACT.EXPLODE("Evaluation") OR (MAINSUBJECT.EXACT("Clinical effectiveness") OR MAINSUBJECT.EXACT("Medical effectiveness research") OR MAINSUBJECT.EXACT("Effectiveness") OR MAINSUBJECT.EXACT("Organizational effectiveness") OR MAINSUBJECT.EXACT("Cost effectiveness")) OR (MAINSUBJECT.EXACT("Total quality management") OR MAINSUBJECT.EXACT("Impact analysis"))) OR Ti,AB(evaluat* or impact or effectiveness))
N=21

Embase (Ovid) 1996 to 2019 Week 09
1 ((co-ordinat* or coordinat* or integrat* or continuity) adj3 (care or service*)).tw,kw. (45502)
2 integrated health care system/ (9937)
3 transitional care/ (1940)
4 *patient care/ (50706)
5 (joint adj (working or care or service*)).tw,kw. (813)
6 patient transport/ (20362)
7 ((intermediate or transition* or transfer* or team*) adj3 care).tw,kw. (37753)

8 Intermediate Care Facilities/ (33832)
9 interdisciplinary communication/ (10818)
10 ((interinstitution* or interagenc* or interdisciplin* or interprofession* or multiprofessional* or multidisciplin* or multiagenc*) adj6 (relations* or collaborat* or co-ordinat* or coordinat* or communicat* or care or service*)).tw. (35024)
11 interpersonal communication/ (126578)
12 public relations/ (37525)
13 or/1-12 [care-coordination] (355713)
14 exp medical record/ (194712)
15 information system/ or exp decision support system/ or exp hospital information system/ or medical information system/ or nursing information system/ or exp computerized provider order entry/ (83582)
16 medical informatics/ or nursing informatics/ (19674)
17 (electronic adj3 record*).tw. (56327)
18 ((health or medical or clinical) adj5 (information or record*)).tw. (337393)
19 computer system/ (20773)
20 *medical information system/ (7264)
21 or/14-20 [health records & systems] (510028)
22 data interoperability/ (117)
23 interoperab*.tw,kw. (3928)
24 22 or 23 (3975)
25 21 and 24 [interoperable records - based on theory search 3 interoperability] (2515)
26 *health information exchange/ (3887)
27 information exchang*.tw,kw. (3829)
28 or/25-27 [info exchange or records interoperability] (9666)
29 13 and 28 (1558)
30 exp evaluation study/ (55500)
31 total quality management/ (52914)
32 (evaluat* or impact or effectiveness).tw,kw. (4914881)
33 *health care quality/ (55418)
34 outcome assessment/ (458167)
35 30 or 31 or 32 or 33 or 34 (5284599)
36 29 and 35 (563)
37 limit 36 to conference abstract (137)
38 36 not 37 (426)
39 limit 38 to (english language and yr="2000 -Current") (405)

INSPEC (EI Village) 1896 to present

((((((((evaluat* OR $impact OR $effectiveness) WN KY)) OR (((((({computer network performance evaluation} WN CV) OR ({performance evaluation} WN CV))))))) AND (((((((((((({electronic data interchange} WN CV))))) OR (((interoperab*) WN KY))))
AND
((((($electronic NEAR/3 record* OR $health NEAR/3 record* OR $medical NEAR/3 record* OR $clinical NEAR/3 record* OR $health NEAR/3 $information OR $medical NEAR/3 $information OR $Clinical NEAR/3 $information) WN KY)) OR ((((({electronic health records} WN CV) OR ({medical information systems} WN CV)))))))))) OR ((({health information exchange}) WN KY))))
AND

(((((\$continuity NEAR/3 \$care OR \$Continuity NEAR/3 service* OR integrat* NEAR/3 \$care OR integrat* NEAR/3 service* OR coordinat* NEAR/3 \$care OR Coordinat* NEAR/3 service*) WN KY)) OR ((((\$interinstitution OR \$interagency OR interdisciplin* OR interprofession* OR multiprofession* OR Multidisciplin* OR Multiagenc*) AND (relations* OR Collaborat* OR Coordinat* OR Coordinat* OR Communicat* OR \$care OR service*)) WN KY)) OR ((((\$intermediate NEAR/3 \$care OR transition* NEAR/3 \$care OR transfer* NEAR/3 \$care OR team* NEAR/3 \$care OR \$shared NEAR/3 \$care)) WN KY)) OR (((((({patient care} WN CV) OR ({professional communication} WN CV))))) OR ((({joint care} OR {joint working} OR {joint service*}) WN KY)
AND
(1896-2019 WN YR)))))))) NOT ((1999 OR 1998 OR 1997 OR 1996 OR 1995 OR 1994 OR 1989) WN YR))
n=258

Ovid MEDLINE(R) 1996 to February Week 4 2019
1 ((co-ordinat* or coordinat* or integrat* or continuity) adj3 (care or service*)).tw,kw. (25475)
2 "Delivery of Health Care, Integrated"/ (11164)
3 *"Continuity of Patient Care"/ (7990)
4 (joint adj (working or care or service*)).tw,kw. (437)
5 Patient Transfer/ (6382)
6 *Patient Care Team/ (18805)
7 ((intermediate or transition* or transfer* or team*) adj3 care).tw,kw. (18067)
8 Transitional Care/ (468)
9 Intermediate Care Facilities/ (360)
10 *interprofessional relations/ or *interdisciplinary communication/ (19479)
11 ((Interinstitution* or interagenc* or interdisciplin* or interprofession* or multiprofessional* or multidisciplin* or multiagenc*) adj6 (relations* or collaborat* or co-ordinat* or coordinat* or communicat* or care or service*)).tw,kw. (18762)
12 or/1-11 [care co-ordination] (101963)
13 exp Medical Records/ (86564)
14 information systems/ or decision support systems, clinical/ or health information systems/ or exp management information systems/ (50023)
15 medical informatics/ or medical informatics applications/ or medical informatics computing/ or nursing informatics/ or public health informatics/ (14946)
16 exp Decision Making, Computer-Assisted/ (120599)
17 (electronic adj3 record*).tw. (23090)
18 ((health or medical or clinical) adj5 (information or record*)).tw. (179423)
19 Computer Systems/ (8476)
20 or/13-19 [health records] (410352)
21 Health Information Interoperability/ (74)
22 interoperab*.tw,kw. (2830)
23 21 or 22 (2859)
24 23 and 20 [interoperable records - based on theory search 3 interoperability] (1963)
25 Health Information Exchange/ (671)
26 information exchang*.tw,kf. (2539)
27 or/24-26 [HIE or interoperable records] (4727)
28 12 and 27 (635)

For peer review only

29 "quality of healthcare"/ (50954)
30 Quality Improvement/ (19241)
31 exp program evaluation/ (64176)
32 evaluation studies/ or exp evaluation studies as topic/ (949715)
33 (evaluat* or impact or effectiveness).tw,kw. (2878224)
34 "Outcome Assessment (Health Care)"/ (62896)
35 "Process Assessment (Health Care)"/ (4027)
36 Quality Indicators, Health Care/ (14355)
37 or/29-36 [evaluation] (3547278)
38 28 and 37 [evaluating interoperable care coordination] (273)
39 limit 38 to (english language and yr="2000 -Current") (255)

Ovid MEDLINE(R) and Epub Ahead of Print, In-Process & Other Non-Indexed Citations and Daily 1946 to March 01, 2019
(Same strategy as Ovid MEDLINE(R) 1996 to February Week 4 2019)

Sociological Abstracts (ProQuest) 1952 – present
((MAINSUBJECT.EXACT("Quality") OR MAINSUBJECT.EXACT("Quality of Health Care")) OR (MAINSUBJECT.EXACT("Evaluation") OR MAINSUBJECT.EXACT.EXPLODE("Program Evaluation") OR MAINSUBJECT.EXACT.EXPLODE("Technology Assessment")) OR MAINSUBJECT.EXACT.EXPLODE("Effectiveness"))
AND
(TI,AB (evaluat* or impact or effectiveness))
n=34

Web of Science Core Collection: Citation Indexes (Clarivate Analytics) 1900-present
Indexes=SCI-EXPANDED, SSCI, A&HCI, CPCI-S, CPCI-SSH, ESCI
Timespan=1900-2019
#18 247 #16 AND #13 SCI-EXPANDED, SSCI, A&HCI, CPCI-S, CPCI-SSH, ESCI Timespan=2000-2019
17 251 #16 AND #13
16 7,450,991 #15 OR #14
15 96,954 TOPIC: (quality NEAR/3 (improvement* or healthcare or "health care"))
14 7,392,745 TOPIC: ((evaluat* or impact or effectiveness))
13 678 #12 AND #5
12 14,578 #11 OR #10
11 12,854 TS=(" information exchang*")
10 1,937 #9 AND #8
9 27,738 TOPIC: ((interoperab* or inter-operab*))
8 237,037 #7 OR #6
7 231,092 TOPIC: ((((health or medical or clinical) NEAR/5 (information or record*))))
6 33,877 TOPIC: (((electronic NEAR/3 record*)))
5 103,553 #4 OR #3 OR #2 OR #1
4 29,498 TOPIC: ((Interinstitution* or interagenc* or interdisciplin* or interprofession* or multiprofessional* or multidisciplin* or multiagenc*) near/6

For peer review only - http://bmjopen.bmj.com/site/about/guidelines.xhtml

(relations* or collaborat* or co-ordinat* or coordinat* or communicat* or care or service*))
3 27,214 TOPIC: ((intermediate or transition* or transfer* or team*) near/3 care)
2 3,593 TOPIC: (joint near/1 (working or care or service))
1 52,368 TOPIC: ((co-ordinat* or coordinat* or integrat* or continuity) NEAR/3 (care or service*))

Complementary Searches
Search date: 10/04/2019
Google Scholar forward citation searches of:
Vimarlund V, Olve NG, Scandurra I, Koch S. Organizational effects of information and communication technology (ICT) in elderly homecare: a case study. Health Informatics Journal. 2008;14(3):195-210.

Waterson P, Soares MM, Jacobs K, Eason K, Tutt D, Dent M. Using HIT to deliver integrated care for the frail elderly in the UK: current barriers and future challenges. Work. 2012;41:4490-3.

King G, O'Donnell C, Boddy D, Smith F, Heaney D, Mair FS. Boundaries and e-health implementation in health and social care. BMC Med Inf Decis Mak. 2012;12:100

For peer review only

Medication Reconciliation Searches

What is the nature of medicine reconciliation problem in frail older people population?

Search date: 14/12/2018

Structured database searches

Embase (Ovid) 1996 to 2018 Week 50

Note: records downloaded from line 38(reviews search) and line 42 (qualitative studies search)

1 exp qualitative research/ (58974)

2 exp questionnaire/ (582556)

3 exp interview/ (224600)

4 interview*.tw,kw. (355209)

5 qualitative.tw,kw. (215788)

6 nursing methodology research/ (13443)

7 questionnaire*.mp. (766883)

8 survey*.mp. (963944)

9 ethnolog*.mp. (60189)

10 ethnograph*.mp. (10319)

11 ethnonursing.af. (90)

12 phenomenol*.af. (25685)

13 (grounded adj (theor$ or study or studies or research or analys?s)).af. (12366)

14 (emic or etic or hermeneutic* or heuristic* or semiotic*).af. or (data adj1 saturat*).tw. or participant observ*.tw. (21081)

15 (action research or cooperative inquir* or co operative inquir* or co-operative inquir*).mp. (4349)

16 ((purpos* adj4 sampl*) or (focus adj group*)).af. (61811)

17 (life world or life-world or conversation analys?s or personal experience* or theoretical saturation).mp. (40560)

18 cluster sampl*.mp. (7667)

19 observational method*.af. (2041)

20 content analysis.af. (27040)

21 ((discourse* or discurs*) adj3 analys?s).tw. (2084)

For peer review only

22 narrative analys?s.af. (1180)

23 or/1-22 [Qualitative Filter] (2047405)

24 *medication therapy management/ (3526)

25 exp *medication error/ (7536)

26 ((drug* or medicine* or medication*) adj3 (error* or discrepanc* or reconciliation* or safety)).ti,kw. (12898)

27 ((prescrib* or prescription*) adj3 (safety or error* or discrepanc* or reconciliation)).ti,kw. (1089)

28 (inappropriate prescribing or inappropriate prescription*).ti,kw. (977)

29 exp *inappropriate prescribing/ (1670)

30 ((medicine* or medication) adj3 (review or management)).ti,kw. (5754)

31 "drug utilization review"/ (304)

32 or/24-31 [meds rec] (25664)

33 exp *aged/ (27394)

34 (elderly or geriatric*).tw. (284835)

35 (old* adj1 (age or adult* or person* or patient)).tw. (194590)

36 or/33-35 [focussed older pt] (452120)

37 32 and 36 (2462)

38 limit 37 to (english language and "systematic review" and yr="2000 -Current") (109)

39 23 and 32 and 36 (554)

40 limit 39 to (english language and yr="2000 -Current") (519)

41 limit 40 to conference abstracts (147)

42 40 not 41 (372)

Ovid MEDLINE(R) and Epub Ahead of Print, In-Process & Other Non-Indexed Citations and Daily 1946 to December 12, 2018

Note: records downloaded from line 37 (reviews search) and line 39 (qualitative studies search)

1 exp qualitative research/ (42909)

2 qualitative.tw,kf. (197303)

3 Nursing Methodology Research/ (16190)

4 observational study/ (55507)

5 ethnolog*.mp. (153408)

For peer review only

6 ethnograph*.mp. (9570)

7 ethnonursing.af. (108)

8 phenomenol*.af. (23694)

9 (grounded adj (theor$ or study or studies or research or analys?s)).af. (10307)

10 (emic or etic or hermeneutic* or heuristic* or semiotic*).af. or (data adj1 saturat*).tw. or participant observ*.tw. (20663)

11 (action research or cooperative inquir* or co operative inquir* or co-operative inquir*).mp. (3761)

12 ((purpos* adj4 sampl*) or (focus adj group*)).af. (54815)

13 (life world or life-world or conversation analys?s or personal experience* or theoretical saturation).mp. (14207)

14 cluster sampl*.mp. (6469)

15 observational method*.af. (675)

16 content analysis.af. (22875)

17 ((discourse* or discurs*) adj3 analys?s).tw. (1956)

18 narrative analys?s.af. (1061)

19 or/1-18 [qualitative] (515468)

20 "Surveys and Questionnaires"/ (413145)

21 INTERVIEW/ (28252)

22 interviews.tw,kf. (150544)

23 or/19-22 [qual & interviews] (970120)

24 exp *Medication Errors/ (10626)

25 ((drug* or medicine* or medication*) adj3 (error* or discrepanc* or reconciliation* or safety)).ti,kw. (6769)

26 ((prescrib* or prescription*) adj3 (safety or error* or discrepanc* or reconciliation)).ti,kw. (670)

27 ((medicine* or medication) adj3 (review or management)).ti,kw. (3580)

28 "Drug Utilization Review"/ (3575)

29 (inappropriate prescribing or inappropriate prescription*).ti,kw. (606)

30 *Inappropriate Prescribing/ (1676)

31 or/24-30 [tight meds rec] (21474)

32 exp *aged/ (24847)

33 (elderly or geriatric*).tw. (252221)

34 (old* adj1 (age or adult* or person* or patient)).tw. (166191)

35 or/32-34 (403927)

36 31 and 35 (1803)

37 limit 36 to (english language and yr="2000 -Current" and systematic reviews) (140)

38 23 and 31 and 35 (252)

39 limit 38 to (english language and yr="2000 -Current") (223)

PsycINFO (Ovid) 1806 to December Week 1 2018

Note: records downloaded from line 33 (reviews search) and line 35 (qualitative studies search)

1 exp qualitative research/ (8015)

2 questionnaires/ (17465)

3 interviews/ (8164)

4 interview*.tw,id. (302732)

5 qualitative.tw,id. (148364)

6 questionnaire*.mp. (375718)

7 survey*.mp. (306730)

8 ethnolog*.mp. (2690)

9 ethnograph*.mp. (26946)

10 ethnonursing.af. (138)

11 phenomenol*.af. (106332)

12 (grounded adj (theor$ or study or studies or research or analys?s)).af. (52076)

13 (emic or etic or hermeneutic* or heuristic* or semiotic*).af. or (data adj1 saturat*).tw. or participant observ*.tw. (92483)

14 (action research or cooperative inquir* or co operative inquir* or co-operative inquir*).mp. (8457)

15 ((purpos* adj4 sampl*) or (focus adj group*)).af. (59543)

16 (life world or life-world or conversation analys?s or personal experience* or theoretical saturation).mp. (14066)

17 cluster sampl*.mp. (1423)

18 observational method*.af. (1994)

19 content analysis.af. (51663)

20 ((discourse* or discurs*) adj3 analys?s).tw. (8505)

21 narrative analys?s.af. (6468)

22 or/1-21 [Qualitative Filter] (1118848)

23 ((drug* or medicine* or medication*) adj3 (error* or discrepanc* or reconciliation* or safety)).tw. (3188)

24 ((prescrib* or prescription*) adj3 (safety or error* or discrepanc* or reconciliation)).tw. (270)

25 ((medicine* or medication) adj3 (review or management)).tw. (3003)

26 (inappropriate prescribing or inappropriate prescription*).tw. (245)

27 or/23-26 [med Rec] (6414)

28 geriatric patients/ (12889)

29 (elderly or geriatric*).tw. (71023)

30 (old* adj1 (age or adult* or person* or patient)).tw. (72103)

31 or/28-30 (130504)

32 27 and 31 (732)

33 limit 32 to (english language and "reviews (maximizes specificity)" and yr="2000 -Current") (52)

34 22 and 27 and 31 (209)

35 limit 34 to (english language and yr="2000 -Current") (193)

Web of Science Core Collection: Citation Indexes (Clarivate Analytics) 1900 – present

Indexes=SCI-EXPANDED, SSCI, A&HCI, CPCI-S, CPCI-SSH, ESCI
Timespan=2000-2018
Note: records downloaded from line 40 (reviews search) and line 41(qualitative studies search)

41 414 (#39 AND #36 AND #31) AND LANGUAGE: (English)Timespan=2000-2018

40 274 (#39 AND #36) AND LANGUAGE: (English) AND DOCUMENT TYPES: (Review) Timespan=2000-2018

39 556,063 #38 OR #37

38 310,703 TOPIC: ((old* NEAR/1 (age or adult* or person* or patient)).)

37 307,597 TOPIC: (elderly or geriatric*)

36 17,068 #35 OR #34 OR #33 OR #32

35 4,894 TI=(((medicine* or medication) NEAR/3 (review or management)))

34 1,581 TS=(("inappropriate prescribing" or "inappropriate prescription*"))

33 3,536 TS=((prescrib* or prescription*) NEAR/3 (safety or error* or discrepanc* or reconciliation))

For peer review only

32 7,839 TI=(((drug* or medicine* or medication*) NEAR/3 (error* or discrepanc* or reconciliation* or safety)))

31 5,935,626 #30 OR #29 OR #28 OR #27 OR #26 OR #25 OR #24 OR #23 OR #22 OR #21 OR #20 OR #19 OR #18 OR #17 OR #16 OR #15 OR #14 OR #13 OR #12 OR #11 OR #10 OR #9 OR #8 OR #7 OR #6 OR #5 OR #4 OR #3 OR #2 OR #1

30 29,138 TS=(narrative analys?s)

29 47,161 TS=(discourse analys?s) or TS =(discurs* analys?s)

28 13,874 TS=(constant comparative)

27 429,822 TS=(content analysis)

26 96,128 TS=observational method*

25 110,512 TS=(cluster sampl*)

24 227,063 TS=(lived experience*) OR TS=(life experience*)

23 99,750 TS=(life world) OR TS=(conversation analys?s) OR TS=(theoretical saturation)

22 693,982 TS=(open-ended account*) OR TS=(unstructured account) OR TS=(narrative*) OR TS=(text*)

21 8,614 TS=(purposive sampl*)

20 72,461 TS=(theoretical sampl*)

19 1,660 TS=(biographical method*)

18 134,004 TS=(human science)

17 1,361,559 TS=(field stud*) OR TS=(field research)

16 295,094 TS=(humanistic) OR TS=(existential) OR TS=(experiential) OR TS=(paradigm*)

15 111,334 TS=(action research) OR TS=(co-operative inquir*)

14 1,047,850 TS=(social construct*) OR TS=(postmodern*) OR TS=(post structural*) OR TS=(feminis*) OR TS=(interpret*)

13 325,003 TS=(emic) OR TS=(etic) OR TS=(hermeneutic) OR TS=(heuristic) OR TS=(semiotic) OR TS=(data saturat*) OR TS=(participant observ*)

12 77,757 TS=(life stor*) OR TS=(women's stor*)

11 392,859 TS=(grounded theor*) OR TS=(grounded stud*) OR TS=(grounded research) OR TS=(grounded analys?s)

10 97,714 TS=(phenomenol*)

For peer review only

# 9	329	TS=(ethnological research)
# 8	61	TS=(ethnonursing)
# 7	47,723	TS=(ethnograph*)
# 6	541,312	TS=(questionnaire)
# 5	3,099	TS=(nursing research methodology)
# 4	396,431	TS=(qualitative)
# 3	30,065	TS=(thematic analysis)
# 2	143,321	TS=(theme*)
# 1	457,600	TS=(interview*)

Complementary searches:

Two Google searches were run, first using the phrase 'Nature of Medicine Reconciliation problem' and secondly using the terms 'medicine reconciliation, barriers, ethnography, qualitative'. The first 50 records were screened from each search. A total of 17 references were potentially relevant and included for further screening.

9.2 What are the cognitive thought processes associated with the interpretation of medication information independent of the technology?

Date: 29/11/2018

Complementary searches

We searched Google Scholar in December 2018 using the terms 'medicine reconciliation' 'clinicians cognition' to identify key papers exploring the clinician's rationalisation and making sense of the fragmented disjoint medication information. The first 50 records were screened. This led us to identify a book chapter ; Cook, R., Medicine reconciliation is a window into ordinary work, in Cognitive system engineering P. Smith, Editor. 2017, CRC press: New York.

We hand searched the reference list in Cook, R which led us to identify two further studies.

- Vashitz, G., et al., Making sense of diseases in medication reconciliation. Cognition, Technology & Work, 2011. 13(2): p. 151-158.
- Vashitz, G., et al., How do clinicians reconcile conditions and medications? The cognitive context of medication reconciliation. Cognition, Technology & Work, 2013. 15(1): p. 109-116.

For peer review only

We conducted forward and backward citation searches in Web of Science for the above studies.

Structured database searches

These specific searches were designed to retrieve the Vashitz papers and those with similar concepts

Ovid MEDLINE(R) and Epub Ahead of Print, In-Process & Other Non-Indexed Citations and Daily 1946 to November 27, 2018

1 exp Medication Errors/ (15326)

2 ((drug* or medicine* or medication*) adj3 (error* or discrepanc* or reconciliation* or safety)).ti,kw. (6762)

3 (inappropriate prescribing or inappropriate prescription*).tw,kw. (1562)

4 Inappropriate Prescribing/ (2395)

5 ((prescrib* or prescription*) adj3 (safety or error* or discrepanc* or reconciliation)).ti,kw. (670)

6 ((medicine* or medication) adj3 (review or management)).ti,kw. (3567)

7 *"Drug Utilization Review"/ (2012)

8 or/1-7 [medicines reconciliation] (25073)

9 *Clinical Decision-Making/ (1962)

10 Heuristics/ (297)

11 *decision making/ (37627)

12 (cognit* adj3 (step* or task* or reason* or process* or analys* or error* or mistake*)).tw. (35271)

13 thinking/ (15151)

14 (diagnostic adj2 (knowledge or judgement or reasoning)).tw. (1002)

15 (clinical adj2 (knowledge or judgement or reasoning)).ti,kw. (1897)

16 heuristic*.tw. (10382)

17 ((prescrib* or prescription*) adj2 (choice* or decision* or process*)).ti,kw. (228)

18 or/9-17 [cognitive tasks] (100525)

19 8 and 18 (170)

20 limit 19 to english language (164)

For peer review only

PsycINFO (Ovid) 1806 to November Week 4 2018

1 ((drug* or medicine* or medication*) adj3 (error* or discrepanc* or reconciliation* or safety)).tw. (3185)

2 ((prescrib* or prescription*) adj3 (safety or error* or discrepanc* or reconciliation)).tw. (270)

3 (inappropriate prescribing or inappropriate prescription*).tw. (244)

4 ((medicine* or medication) adj3 (review* or management)).tw. (3409)

5 or/1-4 [medicines reconciliation] (6805)

6 (cognit* adj3 (step* or task* or reason* or process* or analys* or error* or mistake*)).tw. (60429)

7 *Decision Making/ (54907)

8 *"Cognitions"/ (6786)

9 heuristics/ (3326)

10 (diagnostic adj2 (knowledge or judgement or reasoning)).tw. (434)

11 (clinical adj2 (knowledge or judgement or reasoning)).ti. (735)

12 heuristic*.tw. (14130)

13 ((prescrib* or prescription*) adj2 (choice* or decision* or process*)).ti. (56)

14 exp reasoning/ (24798)

15 cognitive processes/ (70647)

16 or/6-15 [cognitive tasks] (200582)

17 5 and 16 (148)

18 limit 17 to english language (144)

Web of Science Core Collection: Citation Indexes (Clarivate Analytics) 1900 – present

Indexes=SCI-EXPANDED, SSCI, A&HCI, CPCI-S, CPCI-SSH, ESCI
Timespan=2000-2018

13 78 (#11 AND #5) AND LANGUAGE: (English)

12 80 #11 AND #5

11 184,189 #10 OR #9 OR #8 OR #7 OR #6

10 350 TI=(((prescrib* or prescription*) NEAR/2 (choice* or decision* or process*)))

9 114,793 TOPIC: (heuristic*)

8 3,348 TI=((clinical NEAR/2 (knowledge or judgement or reasoning)))

7 2,879 TOPIC: ((diagnostic NEAR/2 (knowledge or judgement or reasoning)))

# 6	63,853	TOPIC: ((cognit* NEAR/3 (step* or task* or reason* or process* or analys* or error* or mistake*)))

# 5	17,010	#4 OR #3 OR #2 OR #1

# 4	4,879	TI=(((medicine* or medication) NEAR/3 (review or management)))

# 3	1,565	TOPIC: (("inappropriate prescribing" or "inappropriate prescription*"))

# 2	3,528	TOPIC: ((prescrib* or prescription*) NEAR/3 (safety or error* or discrepanc* or reconciliation))

# 1	7,818	TI=(((drug* or medicine* or medication*) NEAR/3 (error* or discrepanc* or reconciliation* or safety)))

9.3 The clinicians' experiences of using networked HIT to facilitate medicine reconciliation and empirical evidence of the role of networked HIT in medicine reconciliation?

Date: 29 December 2018

Structured database searches

CINAHL (EBSCO) 1981- present

S23	S13 AND S21 Limiters - English Language; Published Date: 20000101-20190131	115

S22	S13 AND S21	118

S21	S14 OR S15 OR S16 OR S17 OR S18 OR S19 OR S20	29,326

S20	TI (inappropriate prescribing or inappropriate prescription*) OR AB (inappropriate prescribing or inappropriate prescription*)	1,077

S19	(MH "Inappropriate Prescribing")	1,790

S18	TI ((medicine* or medication) N3 (review or management)) OR AB ((medicine* or medication) N3 (review or management))	8,238

S17	TI ((prescrib* or prescription*) N3 (safety or error* or discrepanc* or reconciliation)) OR ((prescrib* or prescription*) N3 (safety or error* or discrepanc* or reconciliation))	1,364

S16	TI ((drug* or medicine* or medication*) N3 (error* or discrepanc* or reconciliation* or safety)) OR AB ((drug* or medicine* or medication*) N3 (error* or discrepanc* or reconciliation* or safety))	11,690

S15	(MH "Medication Reconciliation")	1,336

S14	(MH "Medication Errors+")	14,014

For peer review only

S13 S10 OR S11 OR S12 6,167

S12 TI information exchang* OR AB information exchang* 2,695

S11 (MH "Electronic Data Interchange+") OR (MH "Health Care Information Exchange (Iowa NIC)") 3,130

S10 S8 AND S9 1,070

S9 TI (interoperab* or inter-operab*) OR AB (interoperab* or inter-operab*) 1,594

S8 S1 OR S2 OR S3 OR S4 OR S5 OR S6 OR S7 229,710

S7 (MH "Computer Systems") 1,802

S6 TI ((health or medical or clinical) N5 (information or record*)) OR AB ((health or medical or clinical) N5 (information or record*)) 89,751

S5 TI (electronic N3 record*) OR AB (electronic N3 record*) 16,641

S4 (MH "Decision Making, Computer Assisted+") 36,843

S3 (MH "Health Informatics+") 12,213

S2 (MH "Information Systems") OR (MH "Health Information Systems+") 51,203

S1 (MH "Medical Records+") 88,785

Embase (Ovid) 1996 to 2018 Week 53

1 exp medical record/ (192230)

2 information system/ or exp decision support system/ or exp hospital information system/ or medical information system/ or nursing information system/ or exp computerized provider order entry/ (82903)

3 medical informatics/ or nursing informatics.mp. [mp=title, abstract, heading word, drug trade name, original title, device manufacturer, drug manufacturer, device trade name, keyword, floating subheading word, candidate term word] (19823)

4 (electronic adj3 record*).tw. (54793)

5 ((health or medical or clinical) adj5 (information or record*)).tw. (332154)

6 computer system/ (20765)

7 *medical information system/ (7209)

8 or/1-7 [health records and systems] (503935)

9 data interoperability/ (102)

10 interoperab*.tw,kw. (3839)

11 9 or 10 (3879)

For peer review only

12 8 and 11 (2479)

13 information exchange.tw,kw. (3484)

14 or/12-13 (5771)

15 medication therapy management/ (9126)

16 exp medication error/ (16437)

17 ((drug* or medicine* or medication*) adj3 (error* or discrepanc* or reconciliation* or safety)).tw,kw. (33640)

18 ((prescrib* or prescription*) adj3 (safety or error* or discrepanc* or reconciliation)).tw,kw. (3812)

19 (inappropriate prescribing or inappropriate prescription*).tw,kw. (2523)

20 ((medicine* or medication) adj3 (review or management)).tw,kw. (17135)

21 "drug utilization review"/ (314)

22 or/15-21 [medicines reconciliation] (64138)

23 14 and 22 (188)

24 limit 23 to (english language and yr="2000 -Current") (183)

Ovid MEDLINE(R) and Epub Ahead of Print, In-Process & Other Non-Indexed Citations and Daily 1946 to December 26, 2018

1 exp Medical Records/ (137059)

2 information systems/ or decision support systems, clinical/ or health information systems/ or exp management information systems/ (69366)

3 medical informatics/ or medical informatics applications/ or medical informatics computing/ or nursing informatics/ or public health informatics/ (16168)

4 exp Decision Making, Computer-Assisted/ (131543)

5 (electronic adj3 record*).tw. (29521)

6 ((health or medical or clinical) adj5 (information or record*)).tw. (237174)

7 Computer Systems/ (12789)

8 or/1-7 [medical records] (543132)

9 Health Information Interoperability/ (65)

10 interoperab*.tw,kw. (3561)

11 or/9-10 (3586)

12 8 and 11 (2208)

13 Health Information Exchange/ (661)

14 information exchang*.tw,kw. (3343)

For peer review only

15 or/12-14 [HIE or interoperability] (5754)

16 exp Medication Errors/ (15389)

17 ((drug* or medicine* or medication*) adj3 (error* or discrepanc* or reconciliation* or safety)).tw,kw. (20422)

18 (inappropriate prescribing or inappropriate prescription*).tw,kw. (1568)

19 ((prescrib* or prescription*) adj3 (safety or error* or discrepanc* or reconciliation)).tw,kw. (2219)

20 ((medicine* or medication) adj3 (review or management)).tw,kw. (10587)

21 "Drug Utilization Review"/ (3582)

22 or/16-21 [medicines reconciliation] (45032)

23 15 and 22 (130)

24 limit 23 to (english language and yr="2000 -Current") (128)

PsycINFO (Ovid)1806 to December Week 4 2018

1 exp medical records/ (3506)

2 exp information systems/ or decision support systems/ (36629)

3 (electronic adj3 record*).tw. (3681)

4 ((health or medical or clinical) adj5 (information or record*)).tw. (108603)

5 or/1-4 [records] (144480)

6 interoperab*.tw. (513)

7 5 and 6 [interoperable records] (202)

8 information exchang*.tw. (1778)

9 7 or 8 [HIE or interoperability] (1960)

10 ((drug* or medicine* or medication*) adj3 (error* or discrepanc* or reconciliation* or safety)).tw. (3192)

11 ((prescrib* or prescription*) adj3 (safety or error* or discrepanc* or reconciliation)).tw. (271)

12 (inappropriate prescribing or inappropriate prescription*).tw. (246)

13 ((medicine* or medication) adj3 (review or management)).tw. (3008)

14 or/10-13 [medicines reconciliation] (6424)

15 9 and 14 (18)

16 limit 15 to (english language and yr="2000 -Current") (17)

For peer review only

Web of Science Core Collection: Citation Indexes (Clarivate Analytics) 1900 – present
Indexes=SCI-EXPANDED, SSCI, A&HCI, CPCI-S, CPCI-SSH, ESCI
Timespan=2000-2018

14 135 #13 AND #8

13 39,463 #12 OR #11 OR #10 OR #9

12 1,471 TOPIC: ("inappropriate prescribing" or "inappropriate prescription*")

11 14,452 TOPIC: ((medicine* or medication) NEAR/3 (review or management))

10 3,277 TS=((prescrib* or prescription*) NEAR/3 (safety or error* or discrepanc* or reconciliation))

9 23,025 TOPIC: (((drug* or medicine* or medication*) NEAR/3 (error* or discrepanc* or reconciliation* or safety)).)

8 15,833 #7 OR #6

7 11,377 TS=("information exchang*")

6 4,806 #5 AND #4

5 25,504 TOPIC: (interoperab* or inter-operab*)

4 560,712 #3 OR #2 OR #1

3 382,181 TOPIC: ((information or computer* or clinical or health) NEAR/5 system*)

2 199,473 TOPIC: ((health or medical or clinical) NEAR/5 (information or record*))

1 31,329 TOPIC: (electronic NEAR/3 record*)

For peer review only

PRISMA 2009 Checklist

Section/topic	#	Checklist item	Reported on page #
**TITLE**			
Title	1	Identify the report as a systematic review, meta-analysis, or both.	1
**ABSTRACT**			
Structured summary	2	Provide a structured summary including, as applicable: background; objectives; data sources; study eligibility criteria, participants, and interventions; study appraisal and synthesis methods; results; limitations; conclusions and implications of key findings; systematic review registration number.	1
**INTRODUCTION**			
Rationale	3	Describe the rationale for the review in the context of what is already known.	4
Objectives	4	Provide an explicit statement of questions being addressed with reference to participants, interventions, comparisons, outcomes, and study design (PICOS).	5-7
**METHODS**			
Protocol and registration	5	Indicate if a review protocol exists, if and where it can be accessed (e.g., Web address), and, if available, provide registration information including registration number.	1, 5
Eligibility criteria	6	Specify study characteristics (e.g., PICOS, length of follow-up) and report characteristics (e.g., years considered, language, publication status) used as criteria for eligibility, giving rationale.	5-7
Information sources	7	Describe all information sources (e.g., databases with dates of coverage, contact with study authors to identify additional studies) in the search and date last searched.	5-7
Search	8	Present full electronic search strategy for at least one database, including any limits used, such that it could be repeated.	Additional file provided
Study selection	9	State the process for selecting studies (i.e., screening, eligibility, included in systematic review, and, if applicable, included in the meta-analysis).	5-7
Data collection process	10	Describe method of data extraction from reports (e.g., piloted forms, independently, in duplicate) and any processes for obtaining and confirming data from investigators.	7
Data items	11	List and define all variables for which data were sought (e.g., PICOS, funding sources) and any assumptions and simplifications made.	Separate file
Risk of bias in individual studies	12	Describe methods used for assessing risk of bias of individual studies (including specification of whether this was done at the study or outcome level), and how this information is to be used in any data synthesis.	7
Summary measures	13	State the principal summary measures (e.g., risk ratio, difference in means).	N/A

For peer review only

PRISMA 2009 Checklist

Synthesis of results	14	Describe the methods of handling data and combining results of studies, if done, including measures of consistency (e.g., I^2) for each meta-analysis.	7 for synthesis

Section/topic	#	Checklist item	Reported on page #
Risk of bias across studies	15	Specify any assessment of risk of bias that may affect the cumulative evidence (e.g., publication bias, selective reporting within studies).	N/A (but limitations stated on p. 11)
Additional analyses	16	Describe methods of additional analyses (e.g., sensitivity or subgroup analyses, meta-regression), if done, indicating which were pre-specified.	N/A
**RESULTS**			
Study selection	17	Give numbers of studies screened, assessed for eligibility, and included in the review, with reasons for exclusions at each stage, ideally with a flow diagram.	Separate files
Study characteristics	18	For each study, present characteristics for which data were extracted (e.g., study size, PICOS, follow-up period) and provide the citations.	N/A
Risk of bias within studies	19	Present data on risk of bias of each study and, if available, any outcome level assessment (see item 12).	N/A (but limitations stated)
Results of individual studies	20	For all outcomes considered (benefits or harms), present, for each study: (a) simple summary data for each intervention group (b) effect estimates and confidence intervals, ideally with a forest plot.	Separate files
Synthesis of results	21	Present results of each meta-analysis done, including confidence intervals and measures of consistency.	N/A
Risk of bias across studies	22	Present results of any assessment of risk of bias across studies (see Item 15).	N/A
Additional analysis	23	Give results of additional analyses, if done (e.g., sensitivity or subgroup analyses, meta-regression [see Item 16]).	N/A
**DISCUSSION**			
Summary of evidence	24	Summarize the main findings including the strength of evidence for each main outcome; consider their relevance to key groups (e.g., healthcare providers, users, and policy makers).	11
Limitations	25	Discuss limitations at study and outcome level (e.g., risk of bias), and at review-level (e.g., incomplete retrieval of identified research, reporting bias).	11
Conclusions	26	Provide a general interpretation of the results in the context of other evidence, and implications for future research.	12
**FUNDING**			

For peer review only
For peer review only - http://bmjopen.bmj.com/site/about/guidelines.xhtml

PRISMA 2009 Checklist

Funding	27	Describe sources of funding for the systematic review and other support (e.g., supply of data); role of funders for the systematic review.	3

From: Moher D, Liberati A, Tetzlaff J, Altman DG, The PRISMA Group (2009). Preferred Reporting Items for Systematic Reviews and Meta-Analyses: The PRISMA Statement. PLoS Med 6(7): e1000097. doi:10.1371/journal.pmed1000097

For more information, visit: **www.prisma-statement.org**.

For peer review only

For peer review only - http://bmjopen.bmj.com/site/about/guidelines.xhtml

BMJ Open

The effects of inter-organisational information technology networks on patient safety: a realist synthesis

Journal:	*BMJ Open*
Manuscript ID	bmjopen-2019-036608.R1
Article Type:	Original research
Date Submitted by the Author:	06-Jul-2020

Complete List of Authors:	Keen, Justin; University of Leeds, Leeds Institute of Health Sciences<br>Abdulwahid, Maysam; University of Leeds<br>King, Natalie; University of Leeds, Leeds Institute of Health Sciences<br>Wright, Judy; University of Leeds, Leeds Institute of Health Sciences<br>Randell, Rebecca; University of Bradford, Faculty of Health Studies<br>Gardner, Peter<br>Waring, Justin ; University of Birmingham<br>Longo, Roberta; University of Leeds, Leeds Institute of Health Sciences<br>Nikolova, Silviya; Leeds Institute of Health Sciences<br>Sloan, Claire; University of Leeds, Leeds Institute of Health Sciences<br>Greenhalgh, Joanne; University of Leeds, Sociology and Social Policy

**Primary Subject Heading**:	Health informatics
Secondary Subject Heading:	Health policy
Keywords:	Information technology < BIOTECHNOLOGY & BIOINFORMATICS, Health policy < HEALTH SERVICES ADMINISTRATION & MANAGEMENT, Quality in health care < HEALTH SERVICES ADMINISTRATION & MANAGEMENT, Risk management < HEALTH SERVICES ADMINISTRATION & MANAGEMENT

SCHOLARONE™
Manuscripts

I, the Submitting Author has the right to grant and does grant on behalf of all authors of the Work (as defined in the below author licence), an exclusive licence and/or a non-exclusive licence for contributions from authors who are: i) UK Crown employees; ii) where BMJ has agreed a CC-BY licence shall apply, and/or iii) in accordance with the terms applicable for US Federal Government officers or employees acting as part of their official duties; on a worldwide, perpetual, irrevocable, royalty-free basis to BMJ Publishing Group Ltd ("BMJ") its licensees and where the relevant Journal is co-owned by BMJ to the co-owners of the Journal, to publish the Work in this journal and any other BMJ products and to exploit all rights, as set out in our licence.

The Submitting Author accepts and understands that any supply made under these terms is made by BMJ to the Submitting Author unless you are acting as an employee on behalf of your employer or a postgraduate student of an affiliated institution which is paying any applicable article publishing charge ("APC") for Open Access articles. Where the Submitting Author wishes to make the Work available on an Open Access basis (and intends to pay the relevant APC), the terms of reuse of such Open Access shall be governed by a Creative Commons licence – details of these licences and which Creative Commons licence will apply to this Work are set out in our licence referred to above.

Other than as permitted in any relevant BMJ Author's Self Archiving Policies, I confirm this Work has not been accepted for publication elsewhere, is not being considered for publication elsewhere and does not duplicate material already published. I confirm all authors consent to publication of this Work and authorise the granting of this licence.

For peer review only

The effects of inter-organisational information technology networks on patient safety: a realist synthesis

Justin Keen
Leeds Institute of Health Sciences
University of Leeds
Worsley Building
Clarendon Way
Leeds LS2 9NL
England

j.keen@leeds.ac.uk
0113 343 0939
(No fax number available)

Maysam Abdulwahid
Leeds Institute of Health Sciences
University of Leeds
Leeds
England

Natalie King
Leeds Institute of Health Sciences
University of Leeds
Leeds
England

Judy Wright
Leeds Institute of Health Sciences
University of Leeds

For peer review only

Leeds
England

Rebecca Randell
Faculty of Health Studies
University of Bradford
Leeds
England

Peter Gardner
School of Pharmacy and Medical Sciences
University of Bradford
Leeds
England

Justin Waring
Health Services Management Centre
University of Birmingham
Birmingham
England

Roberta Longo
Leeds Institute of Health Sciences
University of Leeds
Leeds
England

Silviya Nikolova
Leeds Institute of Health Sciences
University of Leeds

Claire Sloan
Leeds Institute of Health Sciences
University of Leeds

For peer review only

Leeds
England

Joanne Greenhalgh
School of Sociology and Social Policy
University of Leeds
Leeds
England

For peer review only

Key words

information technology, health information exchange, patient safety, systematic review, realist synthesis

Word count

3600

For peer review only - http://bmjopen.bmj.com/site/about/guidelines.xhtml

Abstract

Objective

Health services in many countries are investing in inter-organisational networks, linking patients' records held in different organisations across a city or region. The aim of the systematic review was to establish how, why, and in what circumstances these networks improve patient safety, fail to do so, or increase safety risks, for people living at home.

Design

Realist synthesis, drawing on both quantitative and qualitative evidence, and including consultation with stakeholders in nominal groups and semi-structured interviews.

Eligibility criteria

The co-ordination of services for older people living at home, and medicine reconciliation for older patients returning home from hospital.

Information sources

17 sources including Medline, Embase, CINAHL, Cochrane Library, Web of Science, ACM Digital Library and Applied Social Sciences Index and Abstracts (ASSIA).

Outcomes

Changes in patients' clinical risks.

Results

We did not find any detailed accounts of the sequences of events that policy makers and others believe will lead from the deployment of interoperable networks to improved patient safety. We were, though, able to identify a substantial number of theory fragments, and these were used to develop programme theories.

For peer review only

There is good evidence that there are problems with the co-ordination of services in general, and the reconciliation of medication lists in particular, and it indicates that most problems are social and organisational in nature. There is also good evidence that doctors and other professionals find interoperable networks difficult to use. There was limited high quality evidence about safety-related outcomes associated with the deployment of interoperable networks.

Conclusions

Empirical evidence does not currently justify claims about the beneficial effects of interoperable networks on patient safety. There appears to be a mismatch between technology-driven assumptions about the effects of networks and the socio-technical nature of co-ordination problems.

Review registration

PROSPERO CRD42017073004

For peer review only

Strengths and limitations of this study

- This is the first systematic review that seeks to explain the effects of extra-hospital information technology networks on patient safety
- The review investigates the distance between policy aspirations and realities in clinical settings
- We were only able to test a limited set of possible explanations for the effects of networks
- Aspects of quality appraisal rely on research team judgements: other teams might make different judgements
- Breadth of coverage was maximised, and to some extent traded off against the overall quality of included articles

Funding

National Institute for Health Research - Health Services and Delivery Research programme, project 16/53/03

Competing interests

None declared

For peer review only

Background

Many people who live in their own homes, and who have a number of health problems, need support from a range of professionals. There is good evidence that treatment and care is often fragmented, and increases patients' safety risks.[1-3] Policy makers and opinion leaders have argued that interoperable networks, which give clinicians access to patient records held in health care organisations across cities and regions, can help to overcome the fragmentation.[3, 4] The networks should, therefore, support safer treatment and care.[5]

Health services have long had many discrete information technology (IT) systems, developed by different suppliers, so that general practitioners (GPs), community nurses, pharmacists and others use different systems. From the 1980's onwards hospital departments also had their own systems for pathology, radiology, operating theatres and so on.[6-8] That is, the IT systems have also been fragmented, and in the views of policy makers also need to be integrated.[9, 10] In practice, the technological task is to link the different systems together in an interoperable network that spans a geographical area, such as a city or county. The networks can be designed in different ways. At one end of a continuum, a network provides clinicians with 'seamless' access to systems across a locality, so that they appear as a single patient record and are easy to navigate. At the other, clinicians can access the various different systems, with their own formats, and have to learn how to navigate each one to locate the information they need. It is not clear what types of network are currently available to clinicians. Systematic review evidence about the use of, and effects of, these networks is relatively limited and mixed.[11, 12] Policy thinking therefore rests largely on assumptions about the value of interoperable networks.

This article reports on the findings of a systematic review, using the realist synthesis method, of the effects of interoperable networks on patient safety, which we defined as quantified changes in patients' clinical risks. The review included all configurations of networks, and effects could be attributable to networks or to a combination of networks and users. There were two strands to the review, focused on the co-ordination of services, and on the reconciliation of medication information, for older people living at home. The review identified policy makers' and other

For peer review only - http://bmjopen.bmj.com/site/about/guidelines.xhtml
For peer review only

stakeholders' assumptions about the ways in which interoperable networks influence clinical processes and outcomes and then evaluated the extent to which evidence about actual deployments support the assumptions (or fail to do so).[13-16]

The realist synthesis included structured database searches of a wide range of databases, supplementary searches and stakeholder consultation. A range of quantitative and qualitative evidence was included. The synthesis is reported according to the Preferred Reporting Items for Systematic Reviews and Meta-Analysis guidelines, and is consistent with RAMESES guidelines.[17, 18] A protocol was developed and submitted to the PROSPERO registry of systematic reviews prior to commencing the review (CRD42017073004).

Design

The realist synthesis was undertaken in two discrete stages, theory development and empirical assessment. Theory development involved the development of programme theories, these being representations of the way(s) in which an intervention is intended to work.[19-21] Structured database and complementary searches were undertaken to identify published theories, or fragments of theories, i.e. theories which covered a part of the sequences of decisions and actions that lead from the intervention to a safety-related outcome. Stakeholder consultation is usual in realist syntheses, and in this study nominal group consultation was used to refine the initial programme theories, and to help to identify appropriate populations and settings for assessment. Assessment was undertaken through structured database and complementary searches, designed to identify empirical evidence to establish whether programme theories worked in the ways that were intended - or did not work, or worked in some other way.

Theory development

Five theory development searches were undertaken, including structured subject searches, a review of England and USA government policies and official reports, named author searches (for Professors David Bates and Robert Wachter), systematic review and a Web of Science 'usage count' (articles with a high

For peer review only - http://bmjopen.bmj.com/site/about/guidelines.xhtml

level of interest) searches (see Figure 1). We were looking for statements that set out authors' reasoning about the effects of interoperable networks, which might be found in a wide range of texts, including editorials and interviews as well as journal articles and book chapters. They could be described using different terms, so we used very broad inclusion criteria and read substantial numbers of full texts. Passages where theories and fragments were described were copied into Word files, or hyperlinks created to long passages. The passages were synthesised into initial programme theories.[22]

Figure 1 about here

We recruited two nominal groups, of national policy makers and of information technology leads responsible for interoperable networks in two regions of England. Consultation with senior managers at NHS Digital led us to identify six national policy makers from three different organisations (NHS Digital, NHS England, Public Health England), to give us a range of perspectives. Five information technology leads, for different localities in the south of England, were identified purposively as leaders in implementing interoperable networks, by NHS colleagues in the north of England. We had no prior links with them. The initial theories, in the form of diagrams and supporting explanatory text, were presented to the nominal groups, and also to a patient and public involvement (PPI) panel. Their members commented on the face validity of the programme theories, suggested ways in which they might be refined, and indicated their priorities for evidence searches. Their comments fed into the identification and development of a main programme theory, and to inform the design of detailed evidence searches.

The nominal groups indicated that, rather than fine-grained analyses of programme theories, they would value evidence about effects on patient safety, and quality of treatment and care more generally. They observed there was little incentive to provide anything beyond functional – basic – interoperability, as many clinicians only used networks 'tactically', when they really needed information. As a result, evidence about functional interoperability would be valuable to them. They also indicated that their priorities for evidence searches, leading us to settle on a single programme theory for assessment, and exploration of two functions of interoperable

For peer review only - http://bmjopen.bmj.com/site/about/guidelines.xhtml

networks – in co-ordinating care across professional and organisational boundaries, and in medicine reconciliation (see Figure 2). The patient and public involvement panel supported these priorities.

Figure 2 about here

Patient and public involvement

The PPI panel met three times in the course of the review and contributed in the following ways:

- Their comments helped us to set priorities for the populations and settings used in the searches;
- They contributed to the interpretation of the initial findings of the evidence searches;
- They commented on our overall interpretation of our findings at the end of the review.

This being a literature review, patients were not recruited to this study. Summaries and full reports of the review have been sent to the nominal and PPI groups.

Evidence searches: data sources and searches

Searches were designed to interrogate the programme theory for two different functions of interoperable networks – the co-ordination of services for older people living in their own homes who were in receipt of two or more services from different organisations and, more specifically, the co-ordination of services for older people living in their own homes who had been prescribed medication from two or more organisations. (We focused on medicine reconciliation involving two or more lists of medications, and lists that were reconciled with a patient assessment undertaken by a clinician.) We undertook three searches for each function, focusing on the nature and extent of co-ordination problems (that interoperable networks might in principle help to address), on users' experiences of using interoperable networks, and on outcomes (defined as changes in patients' risks of harm).

For peer review only - http://bmjopen.bmj.com/site/about/guidelines.xhtml

The designs of the searches for each function were similar, allowing us to compare and contrast findings. All searches included the Medline database bundle 'Ovid MEDLINE® and Epub Ahead of Print, In-Process & Other Non-Indexed Citations and Daily 1946-present'.[18] (Details of additional databases used in each search, and PRISMA flow diagrams are provided in separate files.) Search terms and synonyms were identified by the project team on the basis of a programme theory selected for assessment (see Figure 2). All of the searches were performed and peer-reviewed by information specialists (NK, JW). Structured search strategies were developing using free text words, synonyms and subject index terms organised into search concepts. Further complementary searches, including forward and backward citation searches, were also undertaken.

Study selection

Inclusion and exclusion criteria were designed for each search, with the following inclusion criteria common to all searches:

- Written in the English language;
- Published in 2000 or later.[23]

Articles were assessed for relevance and rigour by three reviewers (MA, JG, JK). Relevance was assessed pragmatically using a 'target', akin to an archery target. Articles that met all inclusion criteria were placed in the 'bullseye'. Those that met only partially met the population (e.g. frail older people) criteria, but met other criteria, were placed in the middle ring. Articles that did not strictly meet either population or intervention criteria, but where it was judged that they might nevertheless shed light on the mechanisms involved, were placed in the outer ring.

Data extraction and quality assessment

For included articles, study identifiers (author, publication year, country), information about the intervention and study methods (methods used, numbers and types of participants), and the evidence itself were recorded in customised spreadsheets. In

For peer review only - http://bmjopen.bmj.com/site/about/guidelines.xhtml

relation to rigour, critical appraisal skills programme (CASP) quality assessment checklists were completed by one member of the team (MA) and reviewed by colleagues (JK or JG) to appraise systematic reviews, narrative and cohort studies.

Synthesis

The empirical evidence for each function was used for two comparisons, namely the functions with one another, and each function with the programme theory.[24] The comparison with the programme theory allowed us to identify the assumptions which were and were not supported by the evidence.[25, 26]

Results

Following study selection and assessment 46 studies were included. Many general statements were found which stated that interoperable networks would improve patient safety. For example, a 2016 report for the US Office of the National Coordinator for Health Information Technology stated that interoperable networks:

> "can improve ... safety by improving the timelines and completeness of important patient health information."[27]

Similarly, a 2016 report for the National Health Service (NHS) in England recommended that the NHS should:

> "…ensure interoperability as a core characteristic of NHS Digital ecosystem - to support clinical care and to promote innovation and research."[28]

The policy argued that the objectives for interoperability included enabling integrated workflow, service redesign, and clinical decision support. We did not find any detailed accounts that described or explained how they would produce safer diagnosis, treatment and care. A substantial number of theory fragments were, though, identified.[12, 29-32] For example, it was argued that interoperable networks would make information available to clinicians wherever and whenever it was needed, enabling integrated workflow and clinical decision support.[28] There were also statements about challenges and risks, associated with a lack of common

For peer review only - http://bmjopen.bmj.com/site/about/guidelines.xhtml

data standards, problems with interfaces and concerns about privacy.[22] These and other fragments were combined to create an initial programme theory.

Evidence searches: care co-ordination

Five reviews were included on the nature of co-ordination problems in services for older people (Table 1). The hand search of King's Fund reports produced two further reviews. The reviews were conducted in different academic traditions, and used different review methods, but produced broadly similar findings. There was good evidence that co-ordination problems were social and organisational in nature. Leadership, organisation cultures and trust influenced the effectiveness of co-ordination. Communication problems were also highlighted. These were typically characterised as a combination of task failures – such as failures to pass key information about a patient to a clinical colleague – and problems with conveying messages to colleagues with different professional backgrounds. Information technologies were rarely mentioned as having a role in either creating or solving problems.

For peer review only

Table 1

The nature of co-ordination and of medicine reconciliation problems

Author and year	*Country*	*Method*	*Topic*	*Data type*	*Rigour*	*Relevance*	*Processes*	*Outcomes/errors*
Co-ordination								
Auschra (2018)**[33]**	-	Systematic review	Barriers to integrated care	Qualitative	**1**	1	Range of inter-personal and institutional issues	-
Threapleton et al (2017)**[2]**	-	Scoping review	Barriers & facilitators to co-ordination	Quantitative (E&O) and qualitative	2	1	Organisational and cultural features of co-ordination	-
Allen et al (2017)**[34]**	-	Narrative review	Transition from hospital to home	Qualitative	1	1	Negotiation and navigation of service user independence	-
Kirst et al (2017)**[35]**	-	Realist review	Conditions for effective team integration	Quantitative (E&O) and qualitative	1	1	Service use, patient and provider experience	Patient health status
Hudson et al (2014)**[36]**	-	Narrative review	Transition from hospital to home	Quantitative (O) and qualitative	2	3	Range of organisational and inter-professional issues	Re-admission rates, user satisfaction with transition
King's Fund (2014)**[37]**	7 countries	Synthesis of case study evidence	Models of integrated care	Qualitative	2	1	Range including flexibility of team working, effective communication, focus on users' needs	Effective integration associated with improved user satisfaction
King's Fund (2013)**[38]**	England	Multi-site case study	Evaluation of care co-ordination programmes	Qualitative	2	1	Range including organisational models, team cultures, engagement	-
Medicine reconciliation								
Godfrey et al (2013)**[39]**	-	Scoping review	Medication management	Quantitative (E&O) and qualitative	2	1	Include time costs of & responsibility for reconciliation, communication problems	Polypharmacy, potentially inappropriate prescribing
Tommelein et al (2015)**[40]**	Europe	Systematic prevalence survey	Potentially inappropriate prescribing	Quantitative (O)	2	3	-	Polypharmacy, patient characteristics including advanced age
Hernandez (2017)**[41]**	USA	Interviews (commu	Co-ordination of services	Qualitative	1	1	Inter-professional co-ordination, communication problems	Polypharmacy, medication errors, adverse events

For peer review only - http://bmjopen.bmj.com/site/about/guidelines.xhtml

		nity nurses)						
Kennelty et al (2015)**[42]**	USA	Interviews (pharmacists)	Reconciliation post hospital discharge	Qualitative	1	1	Resources, communication, inter-professional relationships	-

For peer review only - http://bmjopen.bmj.com/site/about/guidelines.xhtml
For peer review only

Table 2

Co-ordination of services: users' experiences of interoperable networks

Authors/year	*Country*	*Methods*	*Topic*	*Rigour*	*Rel*
Eden (2016)**[43]**	-	Systematic review	HIE barriers and faciliators	1	3
Azarm-Daigle et al (2015) **[44]**	-	Systematic review	Cross-organisational data sharing	1	2
Hoerbst and Schwitzer 2015**[45]**	-	Systematic review	Critical success factors for clinical information systems in integrated care	1	2
Wu et al (2015)**[46]**	USA	Systematic review	HIE barriers and facilitators	1	3
Nicolaisen and Berg (2015) **[47]**	Norway	Primary qualitative: interviews	Perceptions of messaging system	1	2
McMurray et al (2013)**[48]**	Canada	Primary qualitative: ethnographic study	Impact of partial interoperability	1	1

For peer review only

For peer review only - http://bmjopen.bmj.com/site/about/guidelines.xhtml

We included four reviews and two primary studies from the evidence searches for user experiences of interoperable networks in the adult and older population (rather than older people specifically, Table 2). A forward citation search from a 2013 review of the computer-supported cooperative work literature did not yield any further articles.[16] Most evidence was based on interviews, and therefore on subjective reports of behaviour rather than direct observational evidence of users' experiences. Key details were missing from most reports, including information about interface characteristics and other features of the interoperable networks studied. This said, there was a consistent theme, indicating that interoperable networks were difficult to use, with problems reported in accessing networks, searching for and finding relevant information, and hence perceptions that the time costs of these activities were excessive.

Three articles were included from the searches for evidence about patient outcomes. All three were based on interviews with small samples of interviewees, reporting on experiences with networks with limited functionality. There was a common picture of difficulties encountered in accessing and using them, which led to broadly negative perceptions of their value.[49-51] Five review articles were identified as relevant in a subsequent broader search, for evidence about adult patients in general (Table 3). They included quantitative evidence, of variable quality, about intermediate measures including adverse event and hospital re-admission rates. The results were, broadly, positive in the sense that population rates were reported to reduce, implying the possibility of reductions in patients' risks. However, there were also some negative results, and none of the reviews included any quantitative evidence about the effects of interoperable networks on patient outcomes.

For peer review only - http://bmjopen.bmj.com/site/about/guidelines.xhtml

Table 3

Co-ordination of services: service and patient outcomes

Authors/year	*Country*	*Methods*	*Topic*	*Rigour*	*Relevance*
King et al (2012)**[49]**	Scotland	Interviews	Electronic shared assessment tool	1	3
Waterson et al (2012)**[50]**	England	Interviews, observation of meetings	E-health supported care pathway	2	3
Vimarlund et al (2008)**[51]**	Sweden	Interviews	Virtual health record tool	3	3
Health Quality Ontario (2013)**[52]**	-	Systematic review	e-tools, HIE and care co-ordination	1	2
Sadough et al (2018)**[53]**	-	Systematic review	HIE, quality of care	1	2
Hersh et al (2015)**[31]**	-	Systematic review	Effectiveness of HIE	1	2
Reis et al (2017)**[54]**	-	Review of systematic reviews	Cost-benefit of records, HIE, inter-operability	1	2
Menachemi et al (2018)**[55]**	-	Systematic review	HIE and service changes	1	2

Evidence searches: medicine reconciliation

For the nature of medicine reconciliation problems question, one systematic review, one other review and two observational studies were included (Table 1). The overall quality of empirical evidence was reasonable. It indicated that the challenges were social and organisational in nature, the main one being that responsibility for reconciliation is not clear on the ground, particularly following discharge from hospital to home. As a result, responsibility fell between professionals, principally pharmacists, doctors and nurses. Reconciliation could also be viewed as an administrative task (rather than a safety-promoting one), and was not deemed to be important by some professionals. Communication problems emerged clearly as a theme.

Nine articles were identified that shed light on user experiences of medicine reconciliation.[42, 56-63] These were a mix of scenario-based and field-based observational studies. The search for evidence about patient outcomes did not identify any quantitative evidence about changes in patients' risks. Results about proxy outcomes – changes in reconciliation error rates - were mixed. Some articles

For peer review only - http://bmjopen.bmj.com/site/about/guidelines.xhtml

indicated that use of an interoperable network was associated with a measurable reduction in reconciliation errors. Others reported problems with using systems, resulting in no effects on error rates.

Synthesis

We did not find any detailed published accounts that described the ways in which interoperable networks might improve patient safety (or increase patients' risks). For both functions, clinicians found that it was difficult to access networks, and to find and use relevant patient information. The evidence about outcomes, for both functions, was limited.

The programme theory assumed that information would be easy to find and use, and that patients' clinical risks would be reduced: the evidence did not support these elements of the theory. More generally, the programme theory was technology-driven, assuming that the introduction of interoperable networks would lead to improved processes and outcomes.

Discussion

We did not find evidence that policy makers and opinion leaders have thought through the logic of their assumptions about interoperable networks. A key assumption, seemingly widely shared, is that clinicians need access to comprehensive data on their patients, wherever it is held. This has led to the policy prescription of interoperable networks in many countries. The prescription is intuitively reasonable, but our findings indicate that the underlying reasoning is flawed.

There were four main limitations to the study. First, we did not test alternative programme theories about the effects of interoperable networks on patient safety: it is not possible to rule out plausible explanations that have not been considered by policy makers and others, and hence by us. We might, for example, have drawn on complexity or other theories, instead of the views of policy makers and opinion leaders, to develop alternative programme theories. Second, realist synthesis is still

For peer review only - http://bmjopen.bmj.com/site/about/guidelines.xhtml

developing as a systematic review method. The elements of our method have all been reported by other teams, but in slightly different combinations in different studies. Third, aspects of the method involved team judgements. Other research teams might, for example, adopt different criteria for assessing rigour and relevance. It seems reasonable to hope that their results would be broadly similar: our findings are, for example, consistent with reports of poor user experiences with IT systems in other settings.[64, 65] But we cannot be sure of this. A fourth limitation is that we did not find studies of fully integrated networks, and so were not able to explore contributions of different network configurations to the observed process and outcome changes.

Technology-driven reasoning, based on the belief that the introduction of new networks will improve clinical processes and outcomes, appears to be widespread.[66, 67] Our findings point to two problems with the reasoning. First, interoperable networks are usefully thought of as socio-technical systems, wherein any effects result from combinations of technologies and users, rather than the technologies alone.[68, 69] Second, policy thinking implicitly assumes that networks will be easy to access and use. Our evidence indicates that this is not the case in practice, with many reports of difficulties. As far as we are able to tell, given sometimes limited information about interventions, all of the reported studies were conducted on functional networks. Clinicians were able to access other organisations' record systems, but had to navigate the different configurations in each one. We suggest that reports of difficulties accessing and using networks are not, therefore, surprising.

We conclude that policy makers and other stakeholders, including clinicians and suppliers, should examine the mechanistic assumptions that underpin current thinking. They should consider focusing on the everyday organisational realities of working across professional and organisational boundaries, and ways in which networks might make it easier for busy clinicians to co-ordinate their work with one another. Looking ahead, these findings highlight a puzzle about the quality of interoperable networks available to clinicians: there is a need to understand why this continues to be the case. To this end, there is merit in developing policies which set out socio-technical cases for investments in detail. In addition we, in common with

For peer review only - http://bmjopen.bmj.com/site/about/guidelines.xhtml
For peer review only

other authors, believe that primary field studies of users' experiences of interoperable networks, to identify solutions more acceptable to clinicians, are needed.[16]

For peer review only

For peer review only - http://bmjopen.bmj.com/site/about/guidelines.xhtml

Authors' contributions

Peter Gardner, Joanne Greenhalgh, Justin Keen, Roberta Longo, Rebecca Randell, Justin Waring and Judy Wright developed the proposal for the study. Natalie King and Judy Wright designed and undertook structured database searches. Maysam Abdulwahid, Joanne Greenhalgh and Justin Keen undertook screening and data extraction. Peter Gardner and Justin Waring provided specialist input to the design and interpretation of specific searches. All authors, including Silviya Nikolova and Claire Sloan, contributed to the detailed study design and to the interpretation of overall findings. All authors either drafted, or commented on drafts of, this article.

Acknowledgements

We are grateful to the members of the nominal groups, and the PPI panel, for their contributions to the design and conduct of the review.

Data sharing statement

The majority of material in this article is derived from already-published articles and reports. Beyond this, other methods were qualitative and the data generated is not suitable for sharing. Further information can be obtained from the corresponding author.

Checklist

PRISMA checklist uploaded with this article.

Patient consent form

No patients were recruited to this study.

Supplementary data

Separate files are provided – a PRISMA checklist, one file for each Figure, and file with full details of searches undertaken and PRISMA flow diagrams.

For peer review only

For peer review only - http://bmjopen.bmj.com/site/about/guidelines.xhtml

For peer review only

For peer review only - http://bmjopen.bmj.com/site/about/guidelines.xhtml

References

1. Stocker, R., et al., *Care home services at the vanguard: a qualitative study exploring stakeholder views on the development and evaluation of novel, integrated approaches to enhancing healthcare in care homes.* BMJ Open, 2018. **8**(3): p. e017419.
2. Threapleton, D.E., et al., *Integrated care for older populations and its implementation facilitators and barriers: A rapid scoping review.* International Journal for Quality in Health Care, 2017. **29**(3): p. 327-334.
3. Institute of Medicine, *Health IT and patient safety: Building Safer Systems for Better Care*. 2011, Washington (DC): National Academies Press (US).
4. Oliver D, F.C., Humphries R, *Making our health care systems fit for an ageing population*. 2014, The King's Fund: London.
5. World Health Organisation, *Medication without harm*. 2017.
6. Devine, E.B., et al., *Health Information Exchange Use (1990-2015): A Systematic Review.* EGEMS (Wash DC), 2017. **5**(1): p. 27.
7. Provonost P, J.M., Palmer S, Bono R et al (Eds), *Procuring interoperability: achieving high-quality, connected and person-centred care*. 2018, National Academy of Medicine: Washington DC.
8. M, B., *Health care in the age of interoperability part 5: the personal health record.* IEEE Pulse 2019(10): p. 19-23.
9. Everson, J., *The implications and impact of 3 approaches to health information exchange: community, enterprise, and vendor-mediated health information exchange.* Learning Health Systems, 2017. **1**(2): p. e10021.
10. J, A.-M., *How to safely make interoperable Health Information Exchange a Reality.* DePaul Law Review, 2019. **68**(2): p. 197-208.
11. Bowden, T. and E. Coiera, *The role and benefits of accessing primary care patient records during unscheduled care: a systematic review.* BMC Medical Informatics & Decision Making, 2017. **17**(1): p. 138.
12. Rudin, R.S., et al., *Usage and effect of health information exchange: a systematic review.* Ann Intern Med, 2014. **161**(11): p. 803-11.
13. Andreassen, H.K., L.E. Kjekshus, and A. Tjora, *Survival of the project: A case study of ICT innovation in health care.* Social Science & Medicine, 2015. **132**: p. 62-69.
14. Greenhalgh T, W.J., Papoutsi C, Lynch J, Hughes G, A'Court C, Hinder S, Fahy N, Procter R, Shaw S, *Beyond Adoption: A New Framework for Theorizing and Evaluating Nonadoption, Abandonment, and Challenges to the Scale-Up, Spread, and Sustainability of Health and Care Technologies.* JMIR. **19**: p. e367.
15. Williams, R., *Why is it difficult to achieve e-health systems at scale?* Information, Communication & Society, 2016. **19**(4): p. 540-550.
16. Fitzpatrick, G. and G. Ellingsen, *A Review of 25 Years of CSCW Research in Healthcare: Contributions, Challenges and Future Agendas.* Computer Supported Cooperative Work (CSCW), 2013. **22**(4-6): p. 609-665.
17. Geoff Wong, et al., *RAMESES publication standards:meta-narrative reviews.* BMC medicine, 2013. **11**(20): p. 1-15.
18. EUNETHTA, *Guideline: Process of information retrieval for systematic reviews and health technology assessments on clinical effectiveness. Version 1.2.* , E.N.F.H.T. ASSESSMENT, Editor. 2017

For peer review only - http://bmjopen.bmj.com/site/about/guidelines.xhtml

, Institute for Quality and Efficiency in Health Care Germany.
19. Pawson, R., *Evidence-based policy*. 2006, London: SAGE Publications.
20. Bunn, F., et al., *Supporting shared decision making for older people with multiple health and social care needs: a realist synthesis.* BMC Geriatrics, 2018. **18**(1): p. 165.
21. Greenhalgh, T., et al., *Protocol - realist and meta-narrative evidence synthesis: Evolving Standards (RAMESES).* Bmc Medical Research Methodology, 2011. **11**.
22. Shearn, K., et al., *Building Realist Program Theory for Large Complex and Messy Interventions.* International Journal of Qualitative Methods, 2017. **16**(1): p. 1609406917741796.
23. *To Err is Human : Building safer healthcare system* in *Quality of Health Care in America*. 1999, Institue of Medicine USA.
24. Best, A., et al., *Large-System Transformation in Health Care: A Realist Review.* The Milbank Quarterly, 2012. **90**(3): p. 421-456.
25. Bunn, F., et al., *Managing diabetes in people with dementia: a realist review.* Health Technology Assessment (Winchester, England), 2017. **21**(75): p. 1-140.
26. Pollock M, W.R., *Software and Organisations*. 2008, London: Routledge.
27. Graber ML, J.D., Bailey R; for US Department of Health and Human Services Office of the National Coordinator for Health Information Technology. Research Triangle Park, NC: RTI International., *Report of the evidence on health IT safety and interventions and goals and priorities for health care organizations to improve safety using health IT. Final report.* 2016, RTI Internatonal: Research Triangle Park, NC.
28. Wachter, R., *Making IT work : harnessing the power of health information technology to improve care in England : report of the National Advisory Group on Health Information Technology in England.* 2016: London.
29. Marien, S., B. Krug, and A. Spinewine, *Electronic tools to support medication reconciliation: a systematic review.* J Am Med Inform Assoc, 2017. **24**(1): p. 227-240.
30. Bassi, J., F. Lau, and S. Bardal, *Use of information technology in medication reconciliation: a scoping review.* Annals of Pharmacotherapy, 2010. **44**(5): p. 885-97.
31. Hersh, W.R., et al., *Outcomes From Health Information Exchange: Systematic Review and Future Research Needs.* JMIR Med Inform, 2015. **3**(4): p. e39.
32. Zheng, K., et al., *A Survey of the Literature on Unintended Consequences Associated with Health Information Technology: 2014-2015.* Yearbook of Medical Informatics, 2016(1): p. 13-29.
33. Auschra, C., *Barriers to the Integration of Care in Inter-Organisational Settings: A Literature Review.* Int J Integr Care, 2018. **18**(1): p. 5.
34. Allen, J., et al., *User experience and care integration in transitional care for older people from hospital to home: A meta-synthesis.* Qualitative Health Research, 2017. **27**(1): p. 24-36.
35. Kirst, M., et al., *What works in implementation of integrated care programs for older adults with complex needs? A realist review.* International Journal for Quality in Health Care, 2017. **29**(5): p. 612-624.
36. Hudson, R., L. Comer, and R. Whichello, *Transitions in a wicked environment.* Journal of Nursing Management, 2014. **22**(2): p. 201-210.

For peer review only - http://bmjopen.bmj.com/site/about/guidelines.xhtml

37. Goodwin, N., et al., *Providing integrated care for older people with complex needs Lessons from seven international case studies*. 2014.
38. Goodwin, N., et al., *Co-ordinated care for people with complex chronic conditions.* The Kings Fund, 2013.
39. Godfrey, C.M., et al., *Homecare safety and medication management with older adults: A scoping review of the quantitative and qualitative evidence.* JBI Database of Systematic Reviews and Implementation Reports, 2013. **11**(7): p. 82-130.
40. Tommelein, E., et al., *Potentially inappropriate prescribing in community-dwelling older people across Europe: a systematic literature review.* European Journal of Clinical Pharmacology, 2015. **71**(12): p. 1415-27.
41. Hernandez, J., *Medication management in the older adult: A narrative exploration.* Journal of the American Association of Nurse Practitioners, 2017. **29**(4): p. 186-194.
42. Kennelty, K.A., et al., *Barriers and facilitators of medication reconciliation processes for recently discharged patients from community pharmacists' perspectives.* Research in Social & Administrative Pharmacy, 2015. **11**(4): p. 517-530.
43. 39. Eden, K.B., et al., *Barriers and facilitators to exchanging health information: a systematic review.* International journal of medical informatics, 2016. **88**: p. 44-51.
44. Azarm-Daigle, M., C. Kuziemsky, and L. Peyton, *A Review of Cross Organizational Healthcare Data Sharing.* Procedia Computer Science, 2015. **63**: p. 425-32.
45. Hoerbst, A. and M.L. Schweizer, *A Systematic Investigation on Barriers and Critical Success Factors for Clinical Information Systems (CIS) in Integrated Care Settings.* Yearb Med Inform, 2015. **10**(1): p. 79-89.
46. Wu, H. and E. Larue, *Barriers and Facilitators of Health Information Exchange (HIE) Adoption in the United States*, in *Proceedings of the 2015 48th Hawaii International Conference on System Sciences*. 2015, IEEE Computer Society.
47. Nicolaisen, K. and K. Berg, *Electronic communication across organizational borders in healthcare: an empirical study*. 2015, UiT Norges arktiske universitet.
48. McMurray, R., et al., *Managing controversy through consultation: A qualitative study of communication and trust around MMR vaccination decisions.* British Journal of General Practice, 2004. **54**(504): p. 520-525.
49. King, G., et al., *Boundaries and e-health implementation in health and social care.* BMC Medical Informatics & Decision Making, 2012. **12**: p. 100.
50. Waterson, P., et al., *Using HIT to deliver integrated care for the frail elderly in the UK: current barriers and future challenges.* Work, 2012. **41**: p. 4490-4493.
51. Vimarlund, V., et al., *Organizational effects of information and communication technology (ICT) in elderly homecare: a case study.* Health Informatics Journal, 2008. **14**(3): p. 195-210.
52. Health Quality Ontario, *Electronic tools for health information exchange: an evidence-based analysis.* Ont Health Technol Assess Ser, 2013. **13**(11): p. 1-76.
53. Sadoughi, F., S. Nasiri, and H. Ahmadi, *The impact of health information exchange on healthcare quality and cost-effectiveness: A systematic literature review.* Computer Methods and Programs in Biomedicine, 2018. **161**: p. 209-32.

For peer review only - http://bmjopen.bmj.com/site/about/guidelines.xhtml

54. Reis, Z.S.N., et al., *Is There Evidence of Cost Benefits of Electronic Medical Records, Standards, or Interoperability in Hospital Information Systems? Overview of Systematic Reviews.* JMIR Medical Informatics, 2017. **5**(3): p. e26.
55. Menachemi, N., et al., *The benefits of health information exchange: an updated systematic review.* J Am Med Inform Assoc, 2018.
56. Horsky, J., E.A. Drucker, and H.Z. Ramelson, *Higher accuracy of complex medication reconciliation through improved design of electronic tools.* Journal of the American Medical Informatics Association, 2018. **25**(5): p. 465-475.
57. Bitan, Y., et al., *The Cognitive Task of Medication Reconciliation - Clinicians' Approaches to the Arrangement of Medical Condition and Medication History Information.* Proceedings of the Human Factors and Ergonomics Society Annual Meeting, 2016. **60**(1): p. 538-540.
58. van Stiphout, F., et al., *Task analysis of information technology-mediated medication management in outpatient care.* British Journal of Clinical Pharmacology, 2015. **80**(3): p. 415-24.
59. Vashitz, G., et al., *How do clinicians reconcile conditions and medications? The cognitive context of medication reconciliation.* Cognition, Technology & Work, 2013. **15**(1): p. 109-116.
60. Vashitz, G., et al., *Making sense of diseases in medication reconciliation.* Cognition, Technology & Work, 2011. **13**(2): p. 151-158.
61. Foged, S., et al., *Nurses' perspectives on how an e-message system supports cross-sectoral communication in relation to medication administration: A qualitative study.* Journal of Clinical Nursing, 2018. **27**(3-4): p. 795-806.
62. Fanizza, F.A., et al., *Integrating a health information exchange into a community pharmacy transitions of care service.* J Am Pharm Assoc (2003), 2018. **58**(4): p. 442-449.
63. Hohmeier, K.C., et al., *Implementation of a health information exchange into community pharmacy workflow.* J Am Pharm Assoc (2003), 2017. **57**(5): p. 608-615.
64. *What It Will Take To Achieve The As-Yet-Unfulfilled Promises Of Health Information Technology.* Health Affairs, 2013. **32**(1): p. 63-68.
65. GOLD, M. and C. McLAUGHLIN, *Assessing HITECH Implementation and Lessons: 5 Years Later.* The Milbank Quarterly, 2016. **94**(3): p. 654-687.
66. Wachter, R., *The digital doctor : hope, hype, and harm at the dawn of medicine's computer age*. 2015, New York: McGraw Hill Education. xv, 1, 330.
67. Gawande, A., *Why doctors hate their computers*, in *New Yorker*. 2018: New York.
68. Mannion, R. and H. Davies, *Understanding organisational culture for healthcare quality improvement.* BMJ, 2018. **363**: p. k4907.
69. Exworthy, M., M. Powell, and J. Glasby, *The governance of integrated health and social care in England since 2010: great expectations not met once again?* Health Policy, 2017. **121**(11): p. 1124-1130.

For peer review only

Figure 1
Theory Development PRISMA diagram

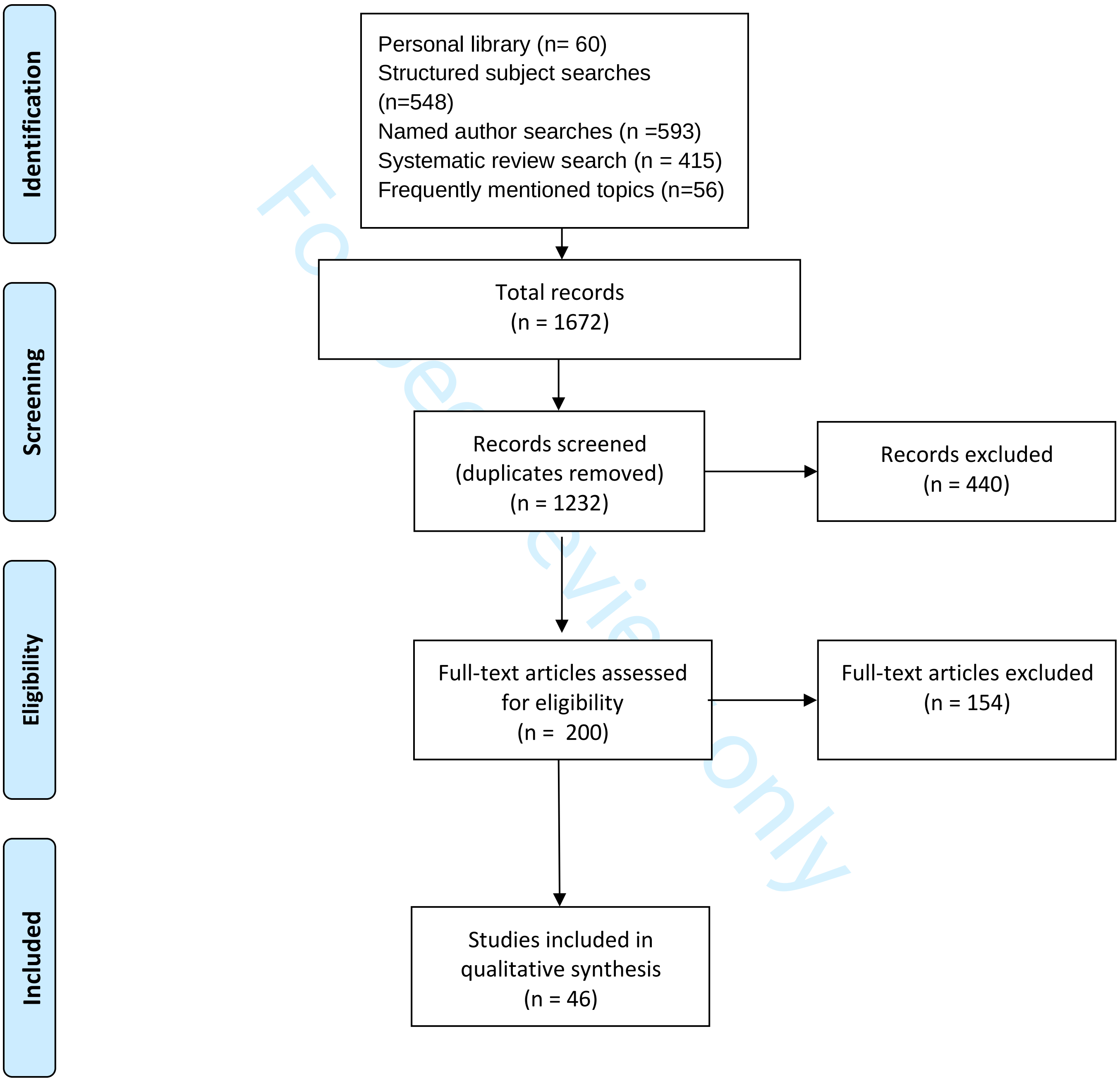

Figure 2: Main programme theory

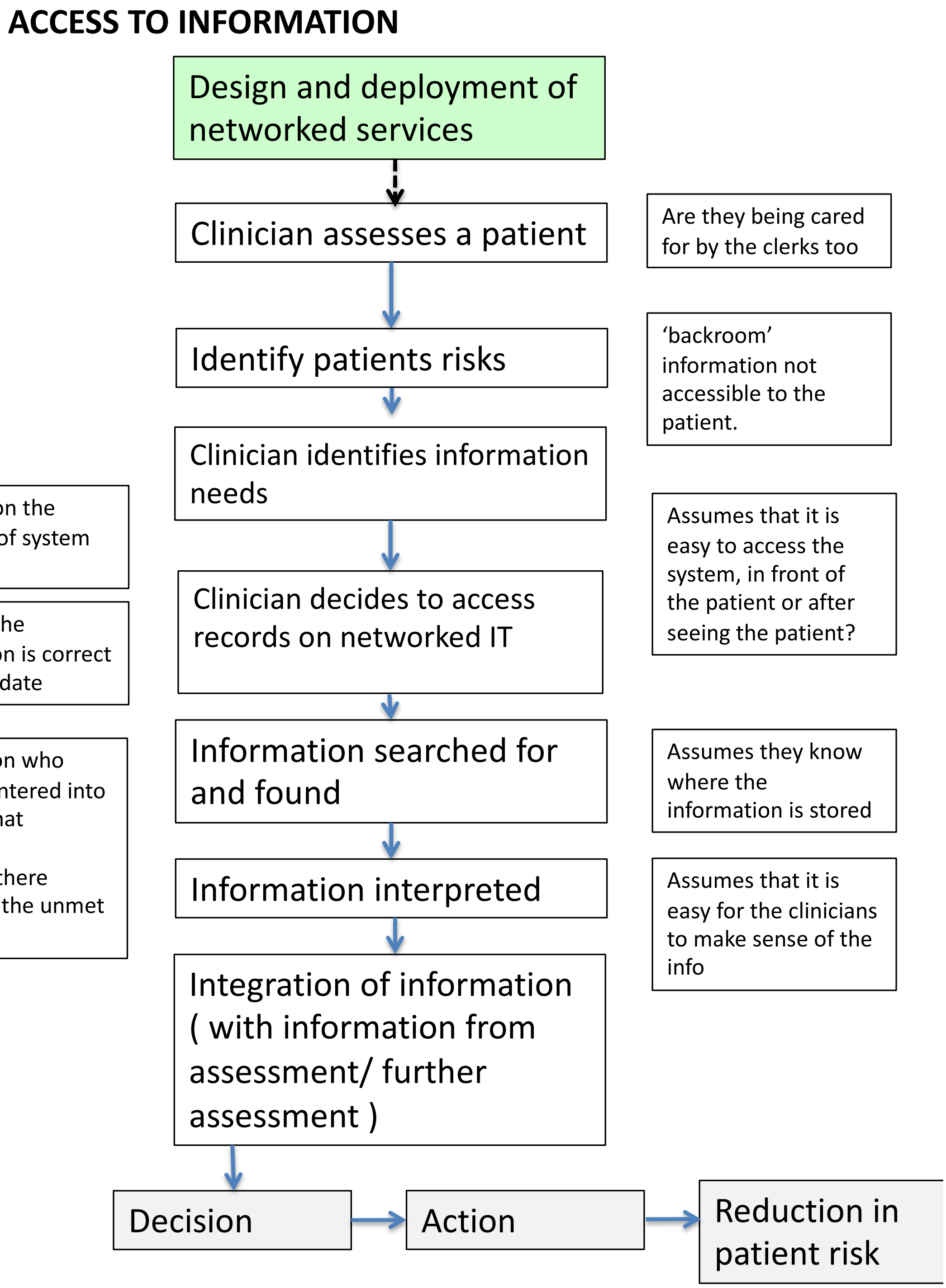

PRISMA 2009 Checklist

Section/topic	#	Checklist item	Reported on page #
**TITLE**			
Title	1	Identify the report as a systematic review, meta-analysis, or both.	1
**ABSTRACT**			
Structured summary	2	Provide a structured summary including, as applicable: background; objectives; data sources; study eligibility criteria, participants, and interventions; study appraisal and synthesis methods; results; limitations; conclusions and implications of key findings; systematic review registration number.	1
**INTRODUCTION**			
Rationale	3	Describe the rationale for the review in the context of what is already known.	4
Objectives	4	Provide an explicit statement of questions being addressed with reference to participants, interventions, comparisons, outcomes, and study design (PICOS).	5-7
**METHODS**			
Protocol and registration	5	Indicate if a review protocol exists, if and where it can be accessed (e.g., Web address), and, if available, provide registration information including registration number.	1, 5
Eligibility criteria	6	Specify study characteristics (e.g., PICOS, length of follow-up) and report characteristics (e.g., years considered, language, publication status) used as criteria for eligibility, giving rationale.	5-7
Information sources	7	Describe all information sources (e.g., databases with dates of coverage, contact with study authors to identify additional studies) in the search and date last searched.	5-7
Search	8	Present full electronic search strategy for at least one database, including any limits used, such that it could be repeated.	Additional file provided
Study selection	9	State the process for selecting studies (i.e., screening, eligibility, included in systematic review, and, if applicable, included in the meta-analysis).	5-7
Data collection process	10	Describe method of data extraction from reports (e.g., piloted forms, independently, in duplicate) and any processes for obtaining and confirming data from investigators.	7
Data items	11	List and define all variables for which data were sought (e.g., PICOS, funding sources) and any assumptions and simplifications made.	Separate file
Risk of bias in individual studies	12	Describe methods used for assessing risk of bias of individual studies (including specification of whether this was done at the study or outcome level), and how this information is to be used in any data synthesis.	7
Summary measures	13	State the principal summary measures (e.g., risk ratio, difference in means).	N/A

PRISMA 2009 Checklist

Synthesis of results	14	Describe the methods of handling data and combining results of studies, if done, including measures of consistency (e.g., I^2) for each meta-analysis.	7 for synthesis

Section/topic	#	Checklist item	Reported on page #
Risk of bias across studies	15	Specify any assessment of risk of bias that may affect the cumulative evidence (e.g., publication bias, selective reporting within studies).	N/A (but limitations stated on p. 11)
Additional analyses	16	Describe methods of additional analyses (e.g., sensitivity or subgroup analyses, meta-regression), if done, indicating which were pre-specified.	N/A
**RESULTS**			
Study selection	17	Give numbers of studies screened, assessed for eligibility, and included in the review, with reasons for exclusions at each stage, ideally with a flow diagram.	Separate files
Study characteristics	18	For each study, present characteristics for which data were extracted (e.g., study size, PICOS, follow-up period) and provide the citations.	N/A
Risk of bias within studies	19	Present data on risk of bias of each study and, if available, any outcome level assessment (see item 12).	N/A (but limitations stated)
Results of individual studies	20	For all outcomes considered (benefits or harms), present, for each study: (a) simple summary data for each intervention group (b) effect estimates and confidence intervals, ideally with a forest plot.	Separate files
Synthesis of results	21	Present results of each meta-analysis done, including confidence intervals and measures of consistency.	N/A
Risk of bias across studies	22	Present results of any assessment of risk of bias across studies (see Item 15).	N/A
Additional analysis	23	Give results of additional analyses, if done (e.g., sensitivity or subgroup analyses, meta-regression [see Item 16]).	N/A
**DISCUSSION**			
Summary of evidence	24	Summarize the main findings including the strength of evidence for each main outcome; consider their relevance to key groups (e.g., healthcare providers, users, and policy makers).	11
Limitations	25	Discuss limitations at study and outcome level (e.g., risk of bias), and at review-level (e.g., incomplete retrieval of identified research, reporting bias).	11
Conclusions	26	Provide a general interpretation of the results in the context of other evidence, and implications for future research.	12
**FUNDING**			

For peer review only

For peer review only - http://bmjopen.bmj.com/site/about/guidelines.xhtml

PRISMA 2009 Checklist

Funding	27	Describe sources of funding for the systematic review and other support (e.g., supply of data); role of funders for the systematic review.	3

From: Moher D, Liberati A, Tetzlaff J, Altman DG, The PRISMA Group (2009). Preferred Reporting Items for Systematic Reviews and Meta-Analyses: The PRISMA Statement. PLoS Med 6(7): e1000097. doi:10.1371/journal.pmed1000097

For more information, visit: www.prisma-statement.org.

For peer review only

For peer review only - http://bmjopen.bmj.com/site/about/guidelines.xhtml
